# Nanostructure-manipulated filtration performance in nanocomposite membranes: A comprehensive investigation for water and wastewater treatment

**DOI:** 10.1016/j.heliyon.2024.e36874

**Published:** 2024-08-26

**Authors:** Fateme Tahmasebi Sefiddashti, Maryam Homayoonfal

**Affiliations:** Department of Chemical Engineering, College of Engineering, University of Isfahan, P.O. Box 81746-73441, Isfahan, Iran

**Keywords:** Nanocomposite membranes, Functionalized nanostructure, Coated nanostructure, Composite nanostructure, Wastewater treatment

## Abstract

The main objective of this article is to examine one of the most important challenges facing researchers in the field of nanocomposite membranes: what is the most suitable arrangement (unmodified, functionalized, coated, or composite) and the most suitable loading site for the nanostructure? In the review articles published on nanocomposite membranes in recent years, the focus has been either on a specific application area (such as nanofiltration or desalination), or on a specific type of polymeric materials (such as polyamide), or on a specific feature of the membrane (such as antibacterial, antimicrobial, or antifouling). However, none of them have targeted the aforementioned objectives on the efficacy of improving filtration performance (IFP). Through IFP calculation, the results will be repeatable and generalizable in this field. The novelty of the current research lies in examining and assessing the impact of the loading site and the type of nanostructure modification on enhancing IFP. Based on the performed review results, for the researchers who tend to use nanocomposite membranes for treatment of organic, textile, brine and pharmaceutical wastewaters as well as membrane bioreactors, the ${\text{PES}}^{{\text{NH}}_{2}‐\text{PDA}‐\text{Fe}3O4}M$, ${\text{PAN}}^{\text{Fe}3O4/\text{ZrO}2}M$, ${\text{PVDF}}^{\text{CMC}‐\text{ZnO}}M$, $\frac{{\text{AA}}^{\text{AA}‐\text{CuS}}}{\text{PSf}}M$ and ${\text{PVDF}}^{\text{OCMCS}/\text{Fe}3O4}M$ with IFP equal to 132.27, 15, 423.6, 16.025 and 5, were proposed, respectively.

## Highlights

• The best site for nanoparticles is both sub and top layer, simultaneously.
• TiO_2_ is the most useable nanoparticle in fabrication of nanocomposite membranes.
• Functionalization is the most useable method in nanoparticles modification of.
• The highest IFP value is for membranes achieved by modified nanoparticles.

## Graphical abstract

**Figure:**
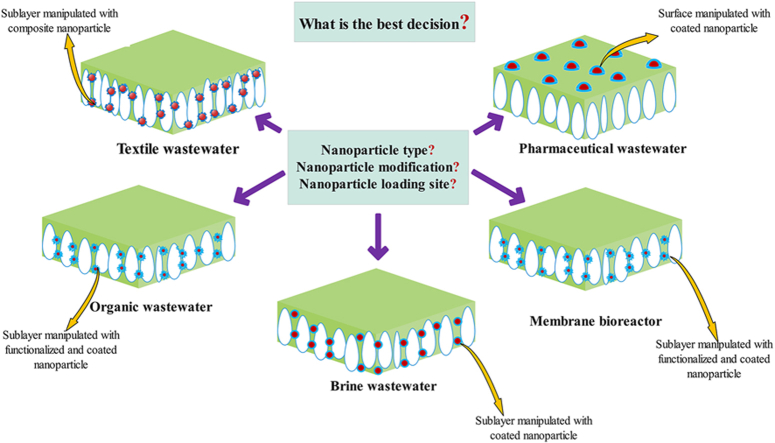

**Table:** Abbreviations

Acrylic acid	AA
Atomic force microscopy	AFM
Cellulose acetate	CA
Cyanuric chloride	CC
Carboxymethylchitosan	CMC
Carbon nanosphere	CNS
Carbon nanotube	CNT
Cephalexin	CEX
Ceftriaxone	CTX
Emulsion poly vinyl chloride	EPVC
Flux recovery ratio	FRR
Graphene oxide	GO
High-density polyethylene	HDPE
1, 6-hexamethylene diisocyanate	HMDA
Halloysite nanotube	HNT
Hydraulic retention time	HRT
Improvement in the filtration performance	IFP
Melamine-based dendrimer amine	MDA
Mixed liquor suspended solid	MLSS
Mix matrix membrane	MMM
Multiwalled carbon nanotube	MWCNT
Nanodiamond	ND
Nanolayered double hydroxide	NLDH
Naringin	Nar
O-carboxymethyl chitosan	OCMCS
Perfluorosulfonic acid	PFSA
Polyamide	PA
Polyacrylonitrile	PAN
Polyaniline	PANI
Polydopamine	PDA
Poly ethylene glycol	PEG
Polyethyleneimine	PEI
Poly ether sulfone	PES
Polyamide	PI
Poly m-phenylene isophthalamide	PMIA
Porous nano-hydroxyapatites	PNHAs
Polystyrene	PS
Polysulfone	PSF
Polyvinyl alcohol	PVA
Poly vinyl chloride	PVC
Polyvinylidene fluoride	PVDF
Polyvinylpyrrolidone	PVP
Polypyrrole	PPY
Reduced graphene oxide	rGO
Sodium alginate	SA
Scanning electron microscopy	SEM
Silicon carbide	SiC
Sulfonated polysulfone	SPSF
Solid retention time	SRT
Transmission electron microscopy	TEM
Vibrating sample magnetometer	VSM

## 1 Introduction

Since deficit of clean water has changed into a progressive global crisis, treatment and recovery of wastewater have turned into a proper alternative for resolving this problem [1]. In recent years, membrane technology has attracted massive attention because of high separation performance, easy operation, and being economical [2]. Improving the flux alongside enhancing the separation efficiency has been the aim of many studies on membrane processes in recent years [3,4]. Since the filtration behavior of the membrane is a function of its surface and structural properties, thus much focus of the mentioned studies has been directed to surface modification [5] or improvement of the structural properties [6] of membranes. From among surface and structural methods, preparation of nanocomposite structures for improving the efficiency of membranes in filtration processes has attracted a great deal of attention in recent years [7].

Nanocomposites are multiphasic compounds, where at least one of the constituent materials of phases has a dimension smaller than 100 nm [8]. The advantage of nanocomposites is related to their surface's multiple activities and the possibility of creating an extraordinary combination of properties, which are impossible in conventional cases [9]. Nanocomposite membranes result from combining nanostructures such as metal nanoparticles [7,10] and metal oxide, clay [11], carbon nanotubes [12], multi-walled carbon nanotubes [13], nanosheets such as graphene oxide [14,15], and graphitic carbon nitride [16], zeolites [17,18], and nanofiber [19,20] with the membrane polymer. From among the different types of the mentioned nanostructures, usage of metal nanoparticles and metal oxide has witnessed greater developments in the fabrication of nanocomposite membranes thanks to their features including simple synthesis, inexpensiveness, diversity of properties, and blending with polymer structures [21]. The metal oxide nanoparticles widely used in the fabrication of nanocomposite membranes include TiO_2_ [22,23], CuS [24], SiO_2_ [25,26], Fe_3_O_4_ [27,28], ZnO [29,30], and ZrO_2_ [31] nanoparticles.

Studies suggest that although the presence of metal oxide nanoparticles has a considerable impact on the membrane filtration parameters such as improving separation, enhancing flux, and reducing fouling. However, events such as aggregation (especially at high loading values [32]), unsuitable distribution within the membrane structure (especially in dense polymeric structures [33]), and separation from the membrane structure during filtration (especially in surface deposition methods [34]), would limit the effectiveness of nanoparticles. Investigations reveal that the extent of effectiveness of nanoparticles on the membrane structure and performance, rather than being related to the intrinsic properties of these particles, is a function of the manner of distribution and their site of loading within the membrane structure [35]. Considering the site of loading in the membrane structure, nanoparticles can be incorporated either as coating on the membrane surface or blended with the polymer matrix [36]. Presence of nanoparticles across the membrane surface has a greater impact on the filtration properties of the membrane. Also, there is possibility of eliminating the embedded nanoparticles during filtration [34]. On the other hand, although blending of nanoparticles with the membrane matrix has less influence on the filtration behavior of the membrane, nanoparticles show greater durability within its structure [37]. Rajaeian et al. [38] employed TiO_2_ nanoparticles for modifying the top layer of the $\frac{{\text{PVA}}^{\text{TiO}2}}{\text{PVDF}}$ M. The obtained results indicated that the extent of separation of the $\frac{{\text{PVA}}^{\text{TiO}2}}{\text{PVDF}}M$ increased by 40 %, while showing 62.5 % decline in the flux. Dong et al. [39] utilized zeolite nanostructures for modifying the $\frac{\text{PA}}{{\text{PSf}}^{\text{Zeolite}}}$ M sublayer. They found that the flux rose by 195 %, while showing 3 % reduction in separation. Thus, it seems that some strategies should be investigated to concurrently benefit from the advantages of both loading methods (surface and structural) and resolving the defects of these methods.

Considering the manner of distribution of nanostructures in the membrane, studies reveal that the manner of distribution is mostly a function of the superficial properties of these particles and the way they interact with the polymer matrix [35]. Thus, adopting strategies that can disperse nanoparticles in the best way across the membrane structure seems necessary, which can offer the best interaction with the polymer matrix and hence the maximum efficiency in improving the membrane performance. One of the most important solutions for suitable distribution of nanoparticles within the membrane structure is modifying the structure of nanoparticles via methods such as donating suitable functional group [40], creating an appropriate coating [41], or combining nanoparticles with desired nanoparticles [42] in order to achieve the best effectiveness in the membrane performance. Ayyaru and Ahn [43], by donating sulfate functional group to the TiO_2_ nanoparticles, synthesized ${\text{PES}}^{S‐\text{TiO}2}$ M, which presented 102.4 % greater flux compared to raw PES membrane, while ${\text{PES}}^{\text{TiO}2}$ M indicated only 62.6 % increase of flux in comparison to the raw PES membrane. Abadikhah et al. [44] by donating amine functional group to the SiO_2_ nanoparticles, synthesized the TFN membrane named $\frac{{\text{PA}}^{\text{NH}2‐\text{SiO}2}}{{\text{PES}/\text{Si}}_{3}N_{4}}M$, which revealed 119 % greater flux compared to $\frac{\text{PA}}{{\text{PES}/\text{Si}}_{3}N_{4}}M$, while $\frac{{\text{PA}}^{\text{SiO}2}}{{\text{PES}/\text{Si}}_{3}N_{4}}M$ indicated only 57 % increase of flux as compared to $\frac{\text{PA}}{{\text{PES}/\text{Si}}_{3}N_{4}}M$. Elsewhere, Puerari et al. [45] synthesized the TFN membrane named $\frac{{\text{PDA}/\text{PEI}}^{\text{NH}2‐\text{SiO}2}}{\text{PSF}}M$ for salt separation which indicated 28 % greater MgCl_2_ rejection compared to $\frac{\text{PDA}/\text{PEI}}{\text{PSF}}M$, while $\frac{{\text{PDA}/\text{PEI}}^{\text{SiO}2}}{\text{PSF}}M$ showed only 7 % MgCl_2_ rejection enhancement compared to $\frac{\text{PDA}/\text{PEI}}{\text{PSF}}M$. In another research, by coating polyvinyl pyrrolidone (PVP) to Fe_3_O_4_ nanoparticles, Hosseini et al. [46] synthesized ${\text{PES}}^{\text{PVP}‐\text{Fe}3O4}$ M, which indicated 106.25 % greater flux compared to the raw PES membrane, while ${\text{PES}}^{\text{Fe}3O4}$ M showed only 34.37 % flux augmentation compared to the raw PES membrane. Kallem et al. [47] synthesized the TFN membrane named $\frac{\text{PA}}{{\text{PES}}^{\text{PDA}‐\text{TiO}2}}M$, which indicated 97 % greater flux compared to $\frac{\text{PA}}{\text{PES}}M$, while $\frac{\text{PA}}{{\text{PES}}^{\text{TiO}2}}M$ showed only 36 % elevation of flux compared to $\frac{\text{PA}}{\text{PES}}M$. By coating tannic acid to TiO_2_ nanoparticles, Li et al. [48] synthesized the TFN membrane named $\frac{{\text{Polyester}}^{\text{TA}‐\text{TiO}2}}{\text{PES}}M$ for salt separation, where the Na_2_SO_4_ rejection increased by 14.6 % compared to $\frac{\text{Polyester}}{\text{PES}}M$. Noormohamadi et al. [49] by compositing Fe_3_O_4_ and ZrO_2_ nanoparticles synthesized ${\text{PAN}}^{\text{ZrO}2/\text{Fe}3O4}$ M, which indicated 34.29 % larger flux compared to ${\text{PAN}}^{\text{ZrO}2}$ M. Safarnia et al. [50] by compositing TiO_2_ and SiO_2_ synthesized the TFN membrane named $\frac{{\text{PDA}}^{\text{SiO}2/\text{TiO}2}}{\text{PVDF}}M$ which indicated 133 % larger flux compared to $\frac{{\text{PDA}}^{\text{SiO}2}}{\text{PVDF}}M$.

Note that in the interpretation and quantitative analysis of studies conducted in this field, issues such as the site of loading (top layer or sublayer) and the structure of nanostructures (composite, coated, and functionalized) have not been involved. In the review articles published in the field of nanocomposite membranes in recent years, the focus of studies has been either on a specific application area (such as nanofiltration [51], desalination [52], or the removal of toxic substances [53]), or on a specific type of polymeric materials (such as polyamide [54]), or on a specific feature of the membrane (such as antibacterial [55], antimicrobial [56], or antifouling [7]). The aforementioned studies have been limited to presenting reports and comparisons among the published studies in that particular area, and none of them have quantitatively compared the filtration performance of all nanocomposite membranes in that area with each other. The novelty of this research is quantitative examination of the effect of the loading site and the method of nanostructure modification (composite, coated, and functionalized) on the performance of membrane filtration (in the form of the IFP parameter). Obviously, when various studies conducted in different area of filtration are examined under a unique parameter (especially IFP in this research), a more accurate judgment will be obtained about the impact of nanostructures on filtration performance, results will be replicable, and the research path for future researchers will be clarified.

Accordingly, in the present review study, first with the aim of resolving the challenge of site of incorporation of nanostructures within the membranous structure and in response to the question of whether to load nanostructures on the membrane surface or matrix, studies were collected, reviewed, and categorized in three fields of nanocomposite membranes with a nanostructure in the surface (P_1_^N1^M and $\frac{{P1}^{N1}}{P2}M$), in the matrix ($\frac{P1}{{P2}^{N2}}M$), and in both surface and matrix layers ($\frac{{P1}^{N1}}{{P2}^{N2}}M$). Then, the best suggestion will be presented for incorporating nanostructures in the membrane matrix. In the second step, in order to resolve the challenge of the manner of distribution of nanoparticles within the membrane structure and in response to the question of the solution for suitable distribution of nanostructures within the membrane structure, practical strategies by the researchers of this field will be studied. In this regard, the most important strategies including functionalizing nanoparticles (F-N), coating nanoparticles (C-N), and compositing nanoparticles (N_1_-N_2_) will be scrutinized and compared. Then, the extent of effectiveness of each of these methods will be compared and presented quantitatively. Finally, the most suitable solutions for the proper distribution of nanostructures within the membranous structure will be described. In the third step, to examine the effectiveness of each of the abovementioned solutions in enhancing the membrane performance in different membrane filtration processes, the performance of nanocomposite membranes will be examined in separation of organic pollutants from wastewater, filtration of textile wastewater, separation of pharmaceutical contaminants from wastewater, separation of salts from wastewater, and treatment of wastewater with membrane bioreactors and compared further. The results are presented in the form of performance comparison of raw, nanocomposite ($\frac{P1}{{P2}^{N2}}M,\frac{{P1}^{N1}}{P2}M,\frac{{P1}^{N1}}{{P2}^{N2}}M$), and modified nanocomposite ($\frac{P1}{{P2}^{m‐N2}}M,\frac{{P1}^{m‐N1}}{P2}M,\frac{{P1}^{m‐N1}}{{P2}^{m‐N2}}M$) membranes. Indeed, the extent of effectiveness of each of the modification strategies in every practical field will be calculated and presented, and the most suitable strategy for each practical field will be explained and suggested. Ultimately, the challenges ahead of each of the strategies are noted and some solutions will be presented for improving these challenges. Therefore, the reason for writing a review article in this field and the most important distinguishing feature of this review article from similar ones published in recent years [7,51,53] is the effort to answer these three questions: 1) Where is the best site for loading nanostructures in the membrane? 2) What is the most suitable nanostructure and the best method for its modification? 3)Which nanocomposite membrane is the most appropriate (considering the type of nanostructure, the method of modifying the loading site) for each of the application areas of the membrane (filtration of organic materials, pharmaceuticals, textiles, desalination, and membrane bioreactor)? Obviously, answering each of the aforementioned questions, while providing new research opportunities for future researchers in this field and providing insight for future development, prepares them to tackle more significant challenges.

## 2 Methodology

In this research, firstly for examining the best site for incorporating nanoparticles within the membrane structure (top layer or sublayer), numerous studies (n = 22) were reviewed. The results of review of the studies have been presented in the form of advantages and disadvantages of loading nanostructures in each layer and introducing the most effective site of loading. In the second part, for exploring the effect of type of nanostructures on improving the membrane performance, extensive studies (n = 256) were examined. The results of investigations and comparisons will cover the pros and cons as well as the extent of effectiveness of each nanostructure on the membrane performance. In the third section, applications of modified nanoparticles including functionalized nanoparticles (N-F), coated nanoparticles (N-C), and composited nanoparticles (N_1_/N_2_) to improve the effectiveness of nanoparticles have been discussed (n = 14 studies). The results of this section have been presented in the form of the effect of modification applied on the surface and structural properties of the membrane as well as comparing the performance of membrane containing modified nanostructure with a membrane containing a raw nanostructure. In the fourth section, the effect of presence of different nanostructures (including both modified and unmodified) at different layers of the membrane (including the top layer and sublayer) was inspected on the filtration performance of the resulting nanocomposite membrane. This investigation has dealt with different fields of applying membrane processes including separation of organic pollutants from wastewater (n = 36 studies), filtration of textile wastewater (n = 16), separation of pharmaceutical pollutants from wastewater (n = 16), separation of salts from wastewater (n = 13), and improving the membrane performance in membrane bioreactors (n = 13). The extent of effectiveness of presence of nanostructures in each field has been calculated precisely and presented further.

Note that in this research, $\frac{{P1}^{N1}}{{P2}^{N2}}M$ symbol has been used for introducing thin-film nanocomposite membranes. Where, P_2_ represents the sublayer polymer, P_1_ shows the top layer polymer, N_1_ indicates the nanostructure employed in the top layer, and N_2_ denotes the nanostructure employed in the sublayer. For introducing the thin-film nanocomposite membranes containing nanostructure only in the top layer or sublayer, $\frac{{P1}^{N1}}{P2}M$ (for presence of nanostructure in the top layer) and $\frac{P1}{{P2}^{N2}}M$ (for presence of nanostructure in the sublayer) symbols have been used. Also, P_1_^N1^M symbol has been utilized for introducing membranes modified via blending method. The results of different studies were concluded from five different fields of separation of organic pollutants, textile wastewater, improving the membrane performance in membrane bioreactors, brine wastewater, and pharmaceutical pollutants. To compare the effect of the presence of nanostructure in different layers, parameters including rejection enhancement and flux enhancement were calculated by Relations 1 and 2 (the calculated data are presented in Table 1). In order to conclude the results presented in each research, depending on the effective parameters in that research field, including nanoparticle concentration (optimal concentration reported by the researcher in that reference), pressure, flux, and pollutant concentration; the parameter of improvement in filtration performance (IFP) was calculated. Use of this comprehensive parameter (instead of flux or rejection) leads to reproducibility of the results and their generalization to research in this field. Considering that the modified membrane flux can be lower than the raw membrane flux, the IFP parameter can also be negative. Relation 3 was presented to explore the effect of the presence of raw and modified nanostructures on the membrane performance (calculated data are presented in Table 2) and organic pollutant separation (with data presented in Table 3). Relations 4, 5, 6, and 7 were presented for dye separation (with data presented in Table 4), MBR (with data presented in Table 5), salt separation (with data presented in Table 6), and drug separation (with data presented in Table 7) respectively.

(1) Rejectionenhancement=Modifiedmembranerejection‐Rawmembranerejection

(2) $$\text{Flux}\;\text{enhancement}=\frac{\text{Modified}\;\text{membrane}\;\text{flux}‐\text{Raw}\;\text{membrane}\;\text{flux}}{\text{Raw}\;\text{membrane}\;\text{flux}\;}\times 100$$

(3) $$\text{Improvement}\;\text{in}\;\text{Filtration}\;\text{Performance}\;\left(\text{IFP}\right)=\frac{\;\text{Flux}\;\text{enhancement}\;}{\;\text{Consumed}\;\text{nanoparticle}}\times 100$$

(4) $$\text{IFP}\;\text{for}\;\text{dye}\;\text{separation}=\frac{\text{Flux}\times \text{Dye}\;\text{Conc}}{\Delta P\times \text{Consumed}\;\text{Nanoparticle}}$$

(5) $$\text{IFP}\;\text{for}\;\text{Application}\;\text{in}\;\text{MBR}=\frac{\text{Flux}\times \text{MLSS}}{\Delta P\times \text{Consumed}\;\text{Nanoparticle}}$$

(6) $$\text{IFP}\;\text{for}\;\text{salt}\;\text{separation}=\frac{\text{Flux}\times \text{Salt}\;\text{Conc}}{\Delta P\times \text{Consumed}\;\text{Nanoparticle}}$$

(7) $$\text{IFP}\;\text{for}\;\text{drug}\;\text{separation}=\frac{\text{Flux}\times \text{Drug}\;\text{Conc}}{\Delta P\times \text{Consumed}\;\text{Nanoparticle}}$$

Where in Equations (1), (2), (3), (4), (5), (6), (7)), the employed parameters are: flux (in LMH), ΔP (in bar), consumed nanoparticle (in wt%), dye concentration (in g/L), MLSS (in kg/L), salt concentration (in g/L), and drug concentration (in g/L).

## 3 Results and discussion

In this research, in order to clarify the questions that will be propounded further, studies conducted on nanocomposite membranes were collected, categorized, reviewed, and concluded.

- Where is the best site for loading nanostructure within the membrane structure?
- What is the most practical type of nanostructure and the best method of modification of nanostructures?
- What is the extent of improvement of the membrane performance resulting from presence of nanostructure in separating pollutants from organic, textile, biological, brine, and pharmaceutical wastewater?

### 3.1 Locating nanostructure within the membrane structure: where is the best site for loading nanostructure within the membrane structure?

**Fig. 1:**
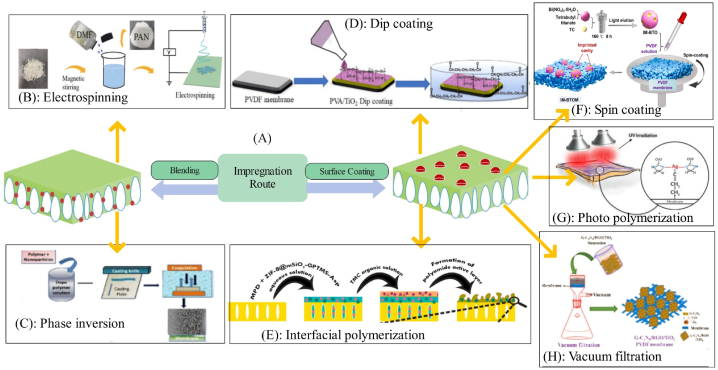
A: Methods of incorporating nanostructure within the membrane; B: Electrospinning (adapted from Ref. [167]), C: Phase inversion (adapted from Ref. [168]), D: Dip coating (adapted from Ref. [165]), E: Interfacial polymerization (adapted from Ref. [163]), F: Spin coating (adapted from Ref. [166]), G: Photo polymerization (adapted from Ref. [164]), H: and Vacuum filtration (adapted from Ref. [162]).

Studies suggest that in addition to the type of nanostructure used, the site of loading of nanostructure within the membrane matrix also considerably affects the filtration performance and structural properties of the membrane [62]. Nanostructures can be blended to the polymer matrix of the sublayer or added to the top layer. As observed in Fig. 1, for loading of nanostructures within the top layer, vacuum filtration [162], interfacial polymerization [163], photo polymerization [164], dip coating [165], and spin coating [166] methods are used. Also, for placing the nanostructure within the sublayer, electrospinning [167] and phase inversion [168] methods can be employed.

**Fig. 2:**
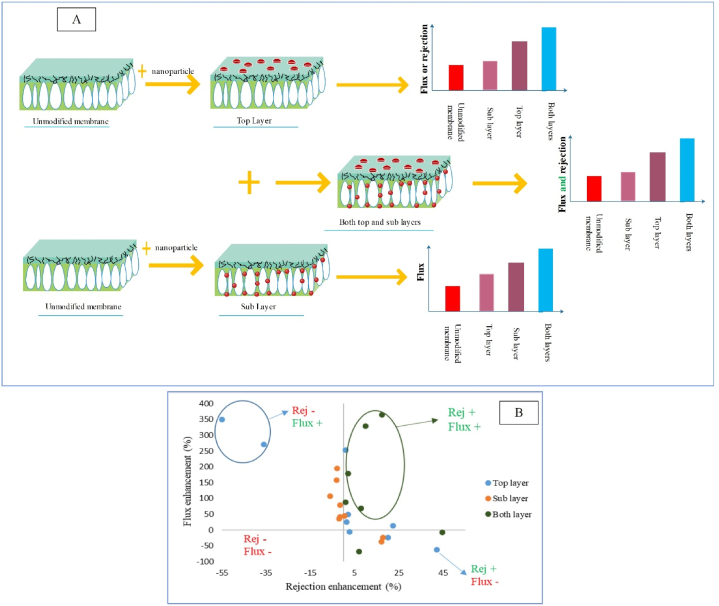
Placement of nanostructure in the sublayer, top layer, and both layers as well as the effect of their presence on the membrane performance, and B: Investigating the effect of site of placement of nanostructure on the flux and rejection enhancement of nanocomposite membranes (More details about the presented data, have been provided in Table 1).

Fig. 2 (A) displays the nanostructure placement within the sublayer, top layer, and both layers as well as its impact on the membrane filtration performance. Based on studies [6,38], and as indicated schematically in Fig. 2 (A), modification of the sublayer results in improvement of the membrane flux, while modification of the top layer would cause enhanced membrane rejection or flux. It seems that modification of the sublayer through adjusting the porosity [62] and enhancing hydrophilicity [39] results in flux enhancement. Modification of the top layer through reducing the size of surface pores [62] has led to augmented rejection [38] or through enhanced surface hydrophilicity [5] it can contribute to elevation of flux [57]. The main advantage of incorporating nanostructure within the sublayer and top layer is uniform distribution of nanoparticles and less consumption of nanostructure respectively. Non-considerable impact on rejection is one of the downsides of loading nanoparticles in the sublayer, while the minimal impact on the general structure is the main disadvantage of loading nanostructure in the top layer. According to studies [70], incorporation of nanostructure in both the top layer and sublayer, in addition to boosting flux through adjustment of porosity would also lead to increased rejection via surface modification (see Fig. 2 (A)). The detailed data of the reviewed studies (about flux and rejection enhancement) are outlined in Fig. 2 (B). Also, in Table 1 and Fig. 2 (B) a comparison has been made between the placement of nanostructure in the top layer, sublayer, and both layers of membrane.

**Table 1:** Investigating the effect of site of placement of nanostructure on the performance of nanocomposite membranes.

Category	Row	Nanocomposite membrane composition		Filtration condition		Filtration performance			Improvement in filtration performance		Reference
		Membrane name	NS Conc. (wt.%)	Type Pollutant	Pollutant Conc. (ppm)	Flux (LMH)	Flux Recovery (%)	Rejection	Flux Enhancement (%)	Rejection Enhancement (%)	
**1:Top-layer embedding**	1	$\frac{{\text{PA}}^{\text{Zeolite}}}{\text{PES}}M$	0.2	NaCl	2000	29.76	–	60	**271**	**−36**	[57]
	2	$\frac{{\text{PVA}}^{\text{TiO}2}}{\text{PVDF}}M$	1	MgSO_4_	1000	75	94	76.44	**−62.5**	**42.3**	[38]
	3	$\frac{{\text{PA}}^{\text{Fe}\left(0\right)}}{\text{PSf}}M$	2	NaCl	2000	17.01	–	90.12	**26**	**1.3**	[5]
	4	$\frac{{\text{PA}}^{\text{Ag}}}{\text{PSf}}M$	0.5	NaCl	2000	22.5	–	10	**350**	**−55**	[58]
	5	$\frac{{\text{PA}}^{\text{rGO}\_\text{TiO}2}}{\text{PSf}}M$^a^	0.02	NaCl	2000	51.3	–	99.45	**49.5**	**2**	[59]
	6	$\frac{{\text{PA}}^{\text{NH}2‐\text{TiO}2}}{\text{PES}}M$	0.3	Na_2_SO_4_	1000	52	93.4	90	**253**	**1**	[60]
	7	$\frac{{\text{PA}}^{\text{Fluorinated}\;\text{silica}}}{\text{PES}}M$	0.12	NaCl	2000	38.75	–	98.6	**−6**	**2.7**	[61]
	8	$\frac{{\text{PA}}^{\text{Fe}3O4/\text{ZrO}2}}{\text{PAN}}M$	0.15	Cephalexin	50	60.35	75.3	75.6	**12.8**	**22.4**	[62]
	9	$\frac{{\text{PA}}^{\text{Ag}/\text{ZnO}}}{\text{PES}}M$	0.3	NaCl	1000	27.4	94.4	59.49	**−23.7**	**20.02**	[63]
**2:Sub-layer embedding**	10	$\frac{\text{PA}}{{\text{PSf}}^{\text{Zeolite}}}$ M	1	MgSO_4_	500	65	–	91	**195**	**−3**	[39]
	11	$\frac{\text{PA}}{{\text{PAN}}^{\text{Fe}3O4}}$ M	1.5	cephalexin	50	33.46	84.4	41.2	**−37.4**	**17.1**	[62]
	12	$\frac{\text{PA}}{{\text{PAN}}^{\text{ZrO}2}}M$	1.5	cephalexin	50	40.62	82.1	41.5	**−24**	**17.8**	
	13	$\frac{\text{PA}}{{\text{PES}}^{\text{TiO}2}}M$	1	NaCl	58440	6.63	–	93.1	**36**	**−2.1**	[47]
	14	$\frac{\text{PA}}{{\text{PSf}}^{\text{PNHAs}}}M$	0.75	NaCl	58440	18.5	–	93.6	**158.4**	**−3.07**	[64]
	15	$\frac{\text{PA}}{{\text{PES}}^{\text{ZnO}}}M$	1	NaCl	1000	32	–	77.94	**107.8**	**−6.17**	[65]
	16	$\frac{\text{PA}}{{\text{PSf}}^{\text{HNT}}}M$	0.5	NaCl	584.4	5	–	93.5	**78.6**	**−1.5**	[66]
	17	$\frac{\text{PA}}{{\text{PSf}}^{\text{Fe}3O4}}M$	0.5	Cadmium	10	2.37	–	98.74	**45.4**	**0.48**	[67]
	18	$\frac{\text{PA}}{{\text{PES}}^{\text{Zeolite}}}M$	0.4	NaCl	1168.8	2.74	–	94.7	**42**	**−1.5**	[68]
**3: Top&Sub-layer embedding**	19	$\frac{{\text{PA}}^{\text{GO}}}{{\text{PSf}}^{\text{GO}}}M$^b^	$\frac{0.76}{1}$	NaCl	2000	15.9	–	98.8	**−69**	**7**	[69]
	20	$\frac{{\text{PA}}^{\text{Zeolite}}}{{\text{PSf}}^{\text{Zeolite}}}M$	$\frac{0.2}{0.2}$	NaCl	584.4	–	–	93	**68**	**8**	[70]
	21	$\frac{{\text{PA}}^{\text{Fe}3O4/\text{ZnO}}}{{\text{PES}}^{\text{Fe}3O4/\text{ZnO}}}M$	$\frac{0.02}{0.2}$	NaCl	584.4	27.2	–	97.93	**87.6**	**1**	[71]
	22	$\frac{{\text{PA}}^{\text{Fe}3O4/\text{ZrO}2}}{{\text{PAN}}^{ZrO2}}$ M	$\frac{0.15}{0.15}$	cephalexin	50	49.9	99. 7	95.8	**−6.6**	**44.6**	[62]
	23	$\frac{{\text{PA}}^{g-C3N4}}{{\text{PSf}}^{\text{HNT}}}M$	$\frac{0.05}{0.5}$	NaCl	1168.8	5.425	98	93	**178.2**	**2**	[72]
	24	$\frac{{\text{PA}}^{\text{ZnO}}}{{\text{PES}}^{\text{TiO}2}}M$	$\frac{0.3}{2}$	NaCl	2000	91.7	–	74.48	**365**	**17.48**	[73]
	25	$\frac{{\text{PA}}^{\text{ZIF}-8}}{{\text{PAN}}^{\text{ZIF}-67}}M$	$\frac{0.13}{0.18}$	doxorubicin	25	21.89	97	99	**329**	**10**	[74]

a rGO: Reduced graphene oxide.

b GO: Graphene oxide.

Investigation of the details of studies listed in Table 1 indicated that addition of nanoparticles to the top layer generally leads to enhanced surface hydrophilicity [5] and thus boosted flux [57], or higher rejection [38] through reducing the size of surface pores [62]. Rajaeian et al. [38] synthesized $\frac{{\text{PVA}}^{\text{TiO}2}}{\text{PVDF}}M$ and although they reported 42.3 % increase in separation, they observed 62.5 % reduction in flux compared to the $\frac{\text{PVA}}{\text{PVDF}}M$ membrane. Mollahosseini and Rahimpour [58] synthesized $\frac{{\text{PA}}^{\text{Ag}}}{\text{PSf}}M$ and in spite of reporting 350 % increase in flux, they witnessed 55 % reduction in rejection compared to the $\frac{\text{PA}}{\text{PSf}}M$ membrane. However, in some studies, the increase of both parameters has been observed [62], though the increase in flux [62] or rejection [60] has been very small. In the research by Karimi et al. [62], around 12 % increase in flux and 22 % growth in rejection were observed for the $\frac{{\text{PA}}^{\text{Fe}3O4/\text{ZrO}2}}{\text{PAN}}M$. The main reason behind this behavior was lack of aggregation of nanoparticles and thus use of modified nanoparticles (composited) in the membrane top layer. Meanwhile, addition of nanoparticles to the sublayer usually causes enhanced hydrophilicity and porosity. Thus, it would considerably augment the membrane flux, though it results in partial increase [6] or even reduction of membrane separation [39]. For example, Dong et al. [39] synthesized $\frac{\text{PA}}{{\text{PSf}}^{\text{Zeolite}}}M$, and found that the flux grew by 195 %, but it showed 3 % reduction of separation compared with the $\frac{\text{PA}}{\text{PSf}}M$. It seems that to concurrently boost the flux and rejection, modified nanostructures in both layers can mostly provide better results as reported in the studies [74]. Thus, it seems that presence of unmodified nanoparticles in a single layer of membrane cannot guarantee considerable increase of flux and separation concurrently (see Fig. 2 (B)).

Nevertheless, simultaneous use of nanostructures in both top layer and sublayer can establish a synergetic effect on improving the structure, surface properties, and filtration behavior of membranes. Although some studies in this regard have still not reported concurrent increase of flux and rejection [62,69], in some other studies, simultaneous enhancement of these two parameters in the presence of unmodified nanostructures has been reported. For example, $\frac{{\text{PA}}^{\text{Zeolite}}\;}{{\text{PSF}}^{\text{Zeolite}}}M$ found 68 % increase in flux and 8 % growth in salt separation compared to the $\frac{\text{PA}}{\text{PSF}}M$ [70]. Thus, since various aims are considered for modifying the top layer and sublayer, it seems that use of nanostructure in both membrane layers can be more effective on achieving a desirable membrane structure, which contributes to elevated flux and rejection concurrently. Accordingly, in case of applying unmodified nanostructure, concurrent presence of nanostructures in both membrane layers is the only way to guaranteeing simultaneous increase of flux and rejection. Ultimately, to explore the effect of presence of nanostructure in the top layer, sublayer, and both layers, the mean increase in rejection was calculated in all three groups, which were obtained as 0.08, −2.23, and 12.87, respectively. Based on the calculations, presence of nanostructure in both top layer and sublayer as well as top layer would lead to a considerable growth in rejection.

### 3.2 Selection of nanostructure: what is the most practical type of nanostructure and best method of nanostructure modification?

**Fig. 3:**
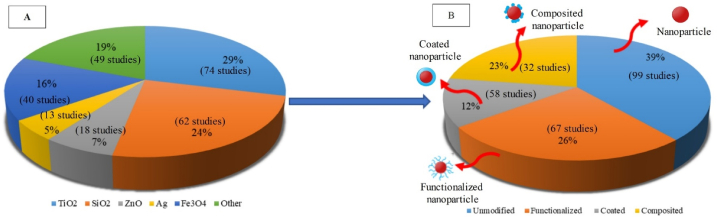
A: Different types of nanostructures used in the membrane structure and B: different types of structural modifications done on nanostructures based on studies published during the years 2010–2023.

Review of 256 studies published during the years 2010–2023 (see further details in Table S1), in which different nanostructures have been used for improving the membrane performance, indicated that TiO_2_ with 29 % (includes 74 studies), SiO_2_ with 24 % (62 studies), and Fe_3_O_4_ nanoparticles with 16 % (40 studies), had the greatest application in fabrication of nanocomposite membranes (see Fig. 3 (A)). Also, use of unmodified and modified nanostructures (through functionalization, coating, and compositing methods) (see more detailed information in Table S2) was examined in membrane fabrication. The results indicated that around 61 % (including 157 studies) of nanostructures has been employed as modified within the membrane structure, whereby functionalization has attracted more attention compared to the other two methods.

**Fig. 4:**
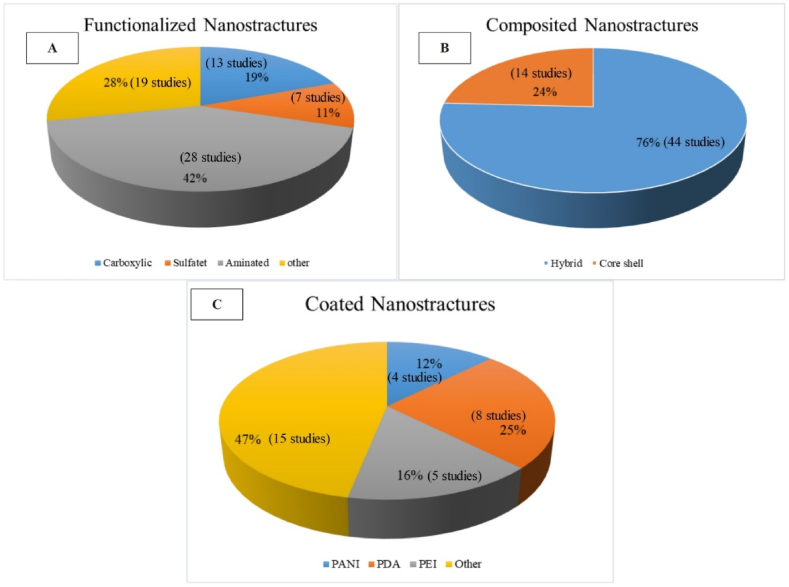
A: Different types of functional groups, B: composite structures, and C: different coatings of nanoparticles based on studies published during the years 2010–2023.

Investigations also revealed that regarding the different types of functional groups used for modification of nanoparticles, based on studies published during the years 2010–2023 (see more information in Table S3), the majority included carboxylic [169,79], sulfate [43], and amine [106,170] functional groups (see Fig. 4(A)). Also, the most widely used coatings included polymer coatings such as polyaniline (PANI) [80], PVP [46], and polydopamine (PDA) [114] (see Fig. 4 (B) and more information in Table S4). Different types of composite structures of nanoparticles also included core shell composites of nanoparticles such as TiO_2_/Fe_3_O_4_ [171] and Fe_3_O_4_/ZrO_2_ [49] or hybrid composites such as TiO_2_/Ag [84],TiO_2_/Al_2_O_3_ [83] (see more information in Table S5). Finally, the statistical data related to the share of application of each of the modifiers has been presented in Fig. 4 (C).

As observed in Figs. 4 and 42 % (including 28 studies) of reported literature has used amine functional groups followed by 19 % (13 studies) applying carboxyl groups for functionalizing nanoparticles. Also, for compositing nanoparticles, 76 % (44 studies) have used hybrid and 24 % (14 studies) have employed core shell method for synthesizing composite nanoparticles. Regarding coating of nanoparticles, usage of PDA with 25 % (8 studies) and PEI with 16 % (5 studies) has been the highest (see more information in Table S4).

To explore the effect of modification of nanoparticles in the membrane performance, Table 2 compares use of unmodified and modified nanoparticles. Considering the diversity of studies regarding the type and concentration of nanoparticles, no definite judgment can be presented regarding membrane performance. Accordingly, the parameter of improvement in filtration performance has been defined by Relation (3). Higher values of this parameter indicate better membrane efficiency and greater impact of modification. In the functionalized nanoparticles group, in a study by Koulivand et al. [75] the IFP value for ${\text{PES}}^{\text{Fe}3O4}M$ membrane was calculated 15. On the other hand, using ${\text{PES}}^{\text{MDA}‐\text{Fe}3O4}M$, IFP was found 62.5. In another study by Monsef et al. [78] using ${\text{PSf}}^{\text{ZrO}2}M$, the IFP was calculated 9.5, and through functionalization with carboxylic acid and sulfate functional groups, the values of this parameter for ${\text{PSf}}^{\text{COOH}‐\text{ZrO}2}M$ and ${\text{PSf}}^{\text{SO}4‐\text{ZrO}2}M$ were achieved as 40 and 40.5, respectively. In the group of coating nanoparticles for ${\text{PES}}^{\text{Fe}3O4}M$ synthesized by Daraei et al. [80] the IFP was calculated −8.3, while by coating nanoparticles in the ${\text{PES}}^{\text{PANI}‐\text{Fe}3O4}M$, this parameter reached 45.45. In another research for ${\text{PSf}}^{\text{SiO}2}M$ and ${\text{PSf}}^{\text{Ce}‐\text{SiO}2}M$, the IFP was calculated as 8 and 13 respectively [81]. In the group of compositing nanoparticles, Noormohamadi et al. [49] using ${\text{PAN}}^{\text{Fe}3O4}M$ reached an IFP of 17.1. On the other hand, employing ${\text{PAN}}^{\text{Fe}3O4/\text{ZrO}2}M$ , the IFP was obtained 42.8. In another research, the IFP for ${\text{CA}}^{\text{TiO}2}M$ and ${\text{CA}}^{\text{TiO}2/\text{Al}2O3}M$ was calculated 5.1 and 8.3 respectively [83]. Further details about the comparison of modified and unmodified nanoparticles are presented in Table 2.

**Table 2:** Comparison of performance of nanocomposite membranes in the presence of raw and modified nanoparticles.

Category	Row	Nanocomposite membrane composition			Filtration condition		Filtration performance		Flux Enhancement/Nanoparticle Loading	Reference
		Membrane name	Modifier	NS/Polymer Conc. (wt.%)	Type Pollutant	Pollutant Conc. (ppm)	Flux (LMH)	Effectiveness of NP modification		
**Functionalized Nanoparticles (F**–**N)**	1	${\text{PES}}^{\text{MDA}-\text{Fe}3O4}M$	MDA	2.38	dye	200	185.7	-CA +FRR +Flow	**62.5**	[75]
		${\text{PES}}^{\text{Fe}3O4}M$	˟				100.9		**15**	
	2	${\text{PES}}^{S-\text{TiO}2}M$	sulfonated	6.67	BSA	500	650	+Porosity +Rej -CA	**16**	[43]
		${\text{PES}}^{\text{TiO}2}M$	˟				520		**9.52**	
	3	${\text{CA}}^{\text{NH}2-\text{ND}}$ M^a^	amino	2.85	COD	6750	52	-CA +Flux +FRR	**86**	[76]
		${\text{CA}}^{\text{ND}}$ M	˟				32		**35**	
	4	${\text{PES}}^{\text{SiO}2-\text{TiO}2}M$	sulfated	11.1	BSA	1000	107	-CA +Flux -Rough +Rej	**7**	[77]
		${\text{PES}}^{\text{TiO}2}M$	˟				70		**1.6**	
	5	${\text{PSf}}^{\text{COOH}-\text{ZrO}2}M$	Carboxylic Acid	2	Dye	100	1015	-CA +Rej	**40**	[78]
		${\text{PSf}}^{\text{SO}4-\text{ZrO}2}M$	Sulfate				1085		**40.5**	
		${\text{PSf}}^{\text{ZrO}2}M$	˟				714		**9.5**	
**Coated Nanoparticles (C**–**N)**	6	${\text{GO}}^{\text{PAA}-\text{NH}2-\text{UiO}-66}M$	PAA	1	Congo red	20	150	+Rej +Rough -CA	**1158**	[79]
		${\text{GO}}^{\text{NH}2-\text{UiO}-66}M$	˟				79		**680**	
	7	${\text{PES}}^{\text{PANI}-\text{Fe}3o4}M$	PANI	0.55	–	–	45	+Flux -CA +FRR	**45.45**	[80]
		${\text{PES}}^{\text{Fe}3o4}M$	˟				33		**−8.3**	
	8	${\text{PES}}^{\text{PVP}-\text{Fe}3o4}M$	PVP	0.55	Na_2_SO_4_	1000	6.6	+Rej +FRR	**193**	[46]
		${\text{PES}}^{\text{Fe}3o4}M$	˟				4.3		**62**	
	9	${\text{PSf}}^{\text{Ce}\_\text{SiO}2}M$	Ce	10	Oil	65	80	-CA +Rej +Flux	**13**	[81]
		${\text{PSf}}^{\text{SiO}2}M$	˟				60		**8**	
**Composited Nanoparticles(N**_**1**_**/N**_**2**_**)**	10	${\text{PVDF}}^{\text{YxFeyZr}1-x-\text{yO}2/\text{TiO}2}M$	YxFeyZr1-x-yO2	2	oil	23.06	400	-CA +Flux	**21.6**	[82]
		${\text{PVDF}}^{\text{TiO}2}M$	˟				335		**9.1**	
	11	${\text{PAN}}^{\text{Fe}3O4/\text{ZrO}2}M$	ZrO_2_	1	dye	100	499	+ Flux -CA + Rej. -Fouling	**42.8**	[49]
		${\text{PAN}}^{\text{Fe}3O4}M$	˟				409		**17.1**	
	12	${\text{CA}}^{\text{TiO}2/\text{Al}2o3}M$	Al_2_O_3_	6.1	NaCl	58440	24.3	-CA +Porosity +Rej +Flux	**8.3**	[83]
		${\text{CA}}^{\text{TiO}2}M$	˟				21.17		**5.1**	
	13	$\frac{\text{PA}\;}{{\text{PSf}}^{\text{TiO}2/\text{Ag}}}M$	Ag	1.15	NaCl	58440	78	+Flux -CA +Rej.	**24.63**	[84]
		$\frac{\text{PA}\;}{{\text{PSf}}^{\text{TiO}2/\text{Ag}}}M$	˟				65		**17.78**	
	14	${\text{PVDF}}^{\text{SnO}2/\text{GO}}M$	GO	6.25	BSA	1000	363.2	+FRR +Flux +Rej -CA	**12.9**	[85]
		${\text{PVDF}}^{\text{SnO}2}M$	˟				300		**7.85**	

a ND: Nanodiamond.

Overall, it can be stated that the average value of IFP for the three groups of functionalized, coated, and composite nanoparticles in Table 2 were equal to 197, 90, and 93, respectively. Based on the obtained results, the efficiency of various nanoparticle modification methods contributes to observation of maximum efficiency and alteration in the membrane structure by applying the minimum concentration of a nanoparticle.

#### 3.2.1 Role of functionalized nanostructure in enhancing membrane performance

**Fig. 5:**
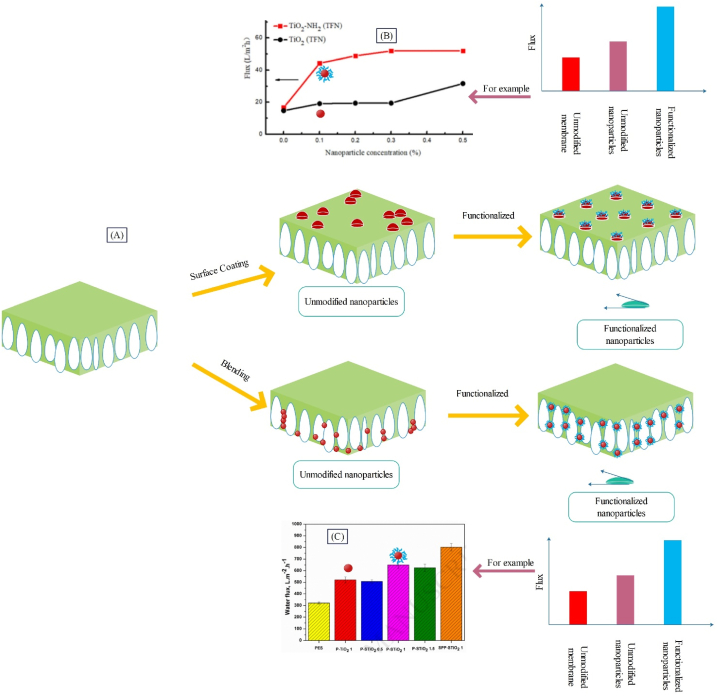
A: Modification of the surface and matrix of membrane with raw and functionalized nanoparticles as well as the effect of this modification on the membrane flux, B: Changing the flux of TFN membrane by adding functionalized nanoparticles to the surface layer (adapted from Ref. [60]), C: Changing the flux of MMM by adding functionalized nanoparticles to the membrane matrix (adapted from Ref. [43]).

When nanoparticles are separated in the membrane matrix, with partial presence on the membrane surface, they somehow affect the surface properties of the membrane, and by manipulating the phase inversion process rate, they influence its structural properties. Usage of a method that provides the possibility of presence of nanoparticles across the membrane surface can boost the effect of nanoparticles on improving the membrane surface properties. Alteration of surface properties of nanoparticles along enhancing hydrophilicity can lead to higher tendency of nanoparticles to be present in coagulation bath along phase inversion and their entrapment within the top layers of the membrane. Depositing hydrophilic functional groups on the surface of nanoparticles to boost their presence in the top layer of membrane can both preserve the effect of nanoparticles on altering the membrane structure and highlight their role as a membrane surface modifier. Now, if the modification created on the surface of nanoparticles, in addition to increasing hydrophilicity, is also associated with augmented antibacterial properties and reduced fouling, this can favor improvements in the anti-fouling properties of the membrane. Fig. 5 (A) displays membrane modification with raw nanoparticles and modified nanoparticles via functionalization method along with the effect of this modification on flux.

As indicated in Fig. 5 (A), nanoparticles can be added to the membrane surface or matrix. Functionalization of nanoparticles leads to uniform distribution of these particles across the membrane surface or matrix. Also, by improving the intrinsic properties of nanoparticles such as hydrophilicity, it contributes to enhancing the membrane flux. The flux passing across membranes containing functionalized nanoparticles is greater than in membranes with unmodified and raw nanoparticles [43,75,78,172]. Using TiO_2_ functionalized nanoparticles, Wei et al. [60] were able to boost water flux in $\frac{{\text{PA}}^{\text{NH}2‐\text{TiO}2}}{\text{PES}}M$ by 253 % and 167 % compared to $\frac{\text{PA}}{\text{PES}}M$ and $\frac{{\text{PA}}^{\text{TiO}2}}{\text{PES}}M$, respectively (see Fig. 5 (B)), while the rejection of membrane $\frac{{\text{PA}}^{\text{NH}2‐\text{TiO}2}}{\text{PES}}M$ increased by only 1 % compared to the raw membrane and reached 95 %. Ayyaru and Ahn [43] synthesized PES^Sulfonated−TiO2^M for separating BSA. According to the results, the flux of PES^Sulfonated−TiO2^M, PES^TiO2^M, and PES membrane was reported 650, 522, and 321 LMH respectively (Fig. 5 (C)). Rajaeian et al. [173] synthesized $\frac{{\text{PA}}^{\;\text{AAPTS}‐\text{TiO}2\;}}{\text{PES}}M$ for separating NaCl. The results indicated that functionalization of nanoparticles led to 142 % increase in water flux for $\frac{{\text{PA}}^{\;\text{AAPTS}‐\text{TiO}2\;}}{\text{PES}}M$ compared to the $\frac{{\text{PA}}^{\;\text{TiO}2\;}}{\text{PES}}M$. The flux of $\frac{\text{PA}}{\text{PES}}M$, $\frac{{\text{PA}}^{\text{TiO}2\;}}{\text{PES}}M$, and $\frac{{\text{PA}}^{\;\text{AAPTS}‐\text{TiO}2\;}}{\text{PES}}M$ was reported 10.7, 11.2, and 27.1 LMH respectively.

**Fig. 6:**
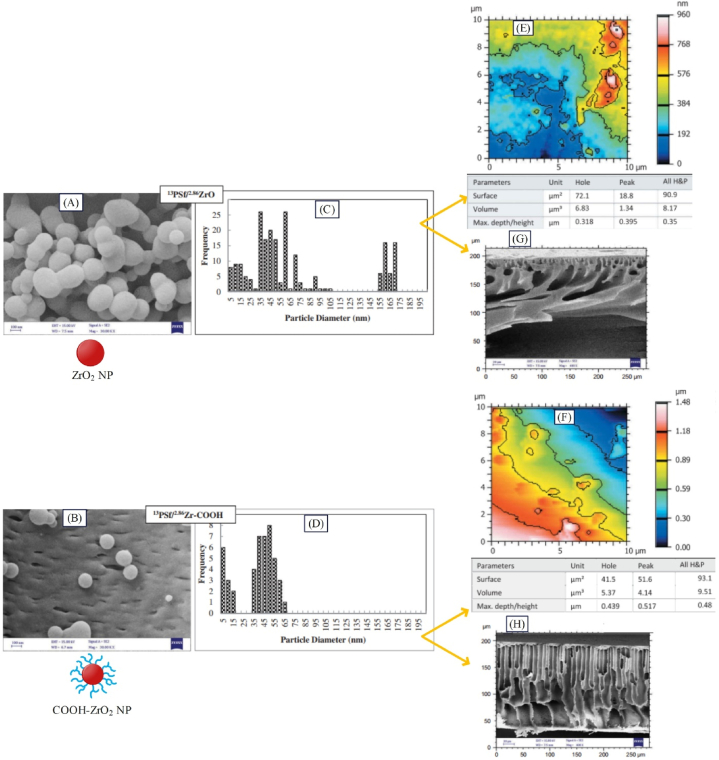
Results of the presence of functionalized nanostructures (COOH-ZrO_2_) on the morphological and topological characteristics of the ${\text{PSf}}^{\text{COOH}-ZrO2}M$: A: the distribution of ZrO_2_, and B: COOH-ZrO_2_ in the polysulfone matrix (SEM image), C: size distribution of ZrO_2_, and D: COOH-ZrO_2_ in the membrane matrix, as well as AFM images of E: ${\text{PSf}}^{\text{ZrO}2}M$, F: ${\text{PSf}}^{\text{COOH}-ZrO2}M$, SEM cross sectional images of G: ${\text{PSf}}^{\text{ZrO}2}M$, and H: ${\text{PSf}}^{\text{COOH}-ZrO2}M$ (adapted from Ref. [78]).

Monsef et al. [78] employed ZrO_2_ nanoparticles for reducing the fouling of polysulfone ultrafiltration membrane in treating textile wastewater. For boosting the role of ZrO_2_ nanoparticles in improving the superficial properties of the membrane, they deposited carboxylic acid functional groups on the surface of ZrO_2_ nanoparticles. As observed in Fig. 6(C and D), the functionalized zirconia nanoparticles after dispersion in the polymer matrix have a narrower size distribution and smaller size compared to the unmodified nanoparticles. It seems that the presence of a functional group on the surface of nanoparticles would prevent accumulation of nanoparticles in the membrane due to the interactions created with the polymer matrix (according to Fig. 6(A and B)) and enhances their effectiveness on the membrane structure properties. Further, based on AFM images (Fig. 6(E and F)), ${\text{PSf}}^{\text{COOH}‐\text{ZrO}2}M$ has greater roughness due to the higher tendency of nanoparticles with functional groups to move towards the water bath, which is expected to elevate the water flux. Based on the SEM images (Fig. 6(G and H)), ${\text{PSf}}^{\text{ZrO}2}M$ has a shorter finger pore length and thus the lowest porosity. In contrast, ${\text{PSf}}^{\text{COOH}‐\text{ZrO}2}M$ has the maximum length of finger pores and the lowest amount of spongy structure as well as maximum porosity due to the hydrophilic nature of the carboxylic acid group, which augments the rate of exchange between water and solvent in the non-solvent bath. Their filtration results indicated that surface functionalization led to 16 % increase in dye separation along with 10 % increment in water flux. The fluxes of ${\text{PSf}}^{\text{ZrO}2}M$ and ${\text{PSf}}^{\text{COOH}‐\text{ZrO}2}M$ were found 500 and 550 LMH respectively [72].

#### 3.2.2 Role of coated nanostructure in improving the membrane performance

**Fig. 7:**
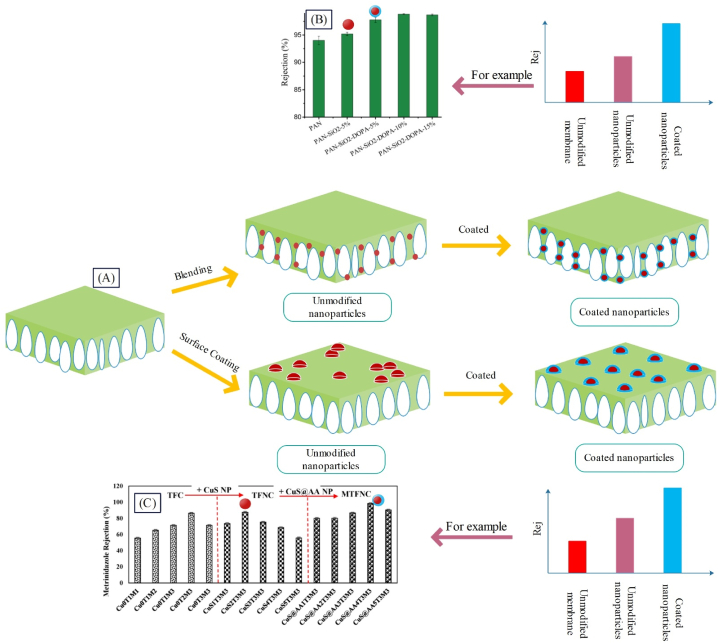
A: Modification of surface and matrix of membrane with raw and coated nanoparticles along with the impact of this modification on the membrane rejection, B: Alteration of the rejection of MMM upon adding coated nanoparticles to the membrane matrix (adapted from Ref. [174]), and C: Changes in the rejection of TFN membrane by adding coated nanoparticles to the surface layer (adapted from Ref. [33]).

Coated nanoparticles (mostly with polymers) are synthesized for more suitable distribution of nanoparticles within the matrix (and layers close to the surface) or in the top layer of membrane. As observed in Fig. 7 (A), coated nanoparticles can be added to the membrane matrix or top layer. Coating of nanoparticles leads to uniform distribution of these particles across the membrane surface or matrix, thereby improving the properties of nanoparticles. The outcome of this more uniform distribution will be enhanced membrane performance as well as increased rejection and flux [33,114,174].

**Fig. 8:**
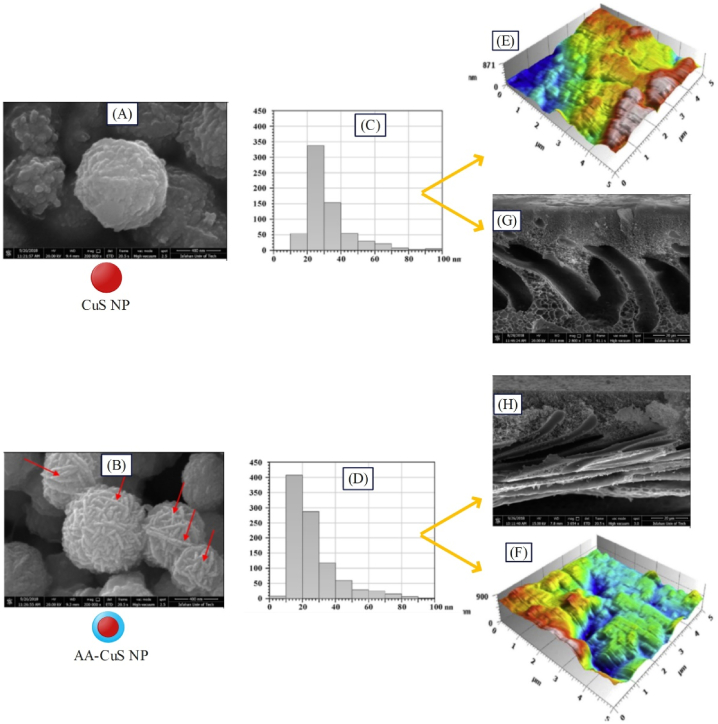
Results of the presence of coated nanostructures (AA-CuS) on the morphological and topological characteristics of $\frac{{\text{AA}}^{\;\text{AA}-\text{CuS}}}{\text{PSf}}M$: the SEM image of A: CuS, and B: AA-CuS nanoparticles, diagram of size distribution of C: CuS, and D: AA-CuS nanoparticles, AFM analysis of E: $\frac{{\text{AA}}^{\;\mathrm{CuS}}}{\text{PSf}}M$, and F: $\frac{{\text{AA}}^{\;\mathrm{AA}-\mathrm{CuS}}}{\text{PSf}}M$, SEM analysis of G: $\frac{{\text{AA}}^{\;\mathrm{CuS}}}{\text{PSf}}M$, and H: $\frac{{\mathrm{AA}}^{\;\mathrm{AA}-\mathrm{CuS}}}{\text{PSf}}M$ (adapted from Ref. [33]).

Sokhandan et al. [175] synthesized PAN^SA−ZnO^M to be used in membrane bioreactors. The employed nanoparticles had antibacterial properties, and SA coating led to enhanced membrane hydrophilicity. They observed that the COD removal percentage for PAN^ZnO^M and PAN^SA−ZnO^M was 88 and 90 % respectively. Tripathi et al. [174] synthesized PAN^PDA−TiO2^M through coating PDA on TiO_2_ nanoparticles for BSA separation, where the rejections of PAN^PDA−TiO2^M, PAN^TiO2^M, and PAN membrane were reported, 98 %, 95 %, and 93.75 %, respectively (see Fig. 7 (B)). Zabihi and Homayoonfal [33] synthesized $\frac{{\text{AA}}^{\;\text{AA}‐\text{CuS}}}{\text{PSf}}$ M for separating metronidazole. The results indicated that the extent of separation for $\frac{\text{AA}}{\text{PSf}}M$, $\frac{{\text{AA}}^{\;\text{CuS}}}{\text{PSf}}M$, and $\frac{{\text{AA}}^{\;\text{AA}‐\text{CuS}}}{\text{PSf}}M$ was 86.3, 87.5, and 98.3 % respectively, confirming the effect of presence of coated nanoparticles on enhancing metronidazole separation (see Fig. 7 (C)). In this membrane, acrylic acid coating was deposited on the surface of nanoparticles for better distribution of nanoparticles in the top layer. Fig. 8 (A, B) indicates the raw and coated CuS nanoparticles in which the chains on the surface of the coated nanoparticles (indicated by red arrows) prove the presence of acrylic acid. As observed in Fig. 8(C and D), AA-CuS nanoparticles are smaller and have a narrower size distribution compared to CuS nanoparticles. The smaller size of AA-CuS nanoparticles and the presence of acrylic acid coating on their surface leads to better dispersion in the monomer solution and better dispersion on the surface of the acrylic layer. As a result, as displayed in Fig. 8(G and H), the number of AA-CuS particles is greater compared to CuS nanoparticles on the surface of the membrane. Accordingly, it can be expected that AA-CuS nanoparticles will be more effective on improving the surface properties of the membrane due to their greater number on the surface. On the other hand, according to Fig. 8(E and F), the roughness of $\frac{{\text{AA}}^{\text{AA}-\text{CuS}}}{\text{PSf}}M$ drops due to the smaller size and better distribution of AA-CuS nanoparticles, which is expected to reduce the surface roughness, leading to a decline in membrane fouling.

#### 3.2.3 Role of composited nanostructure in improving the membrane performance

**Fig. 9:**
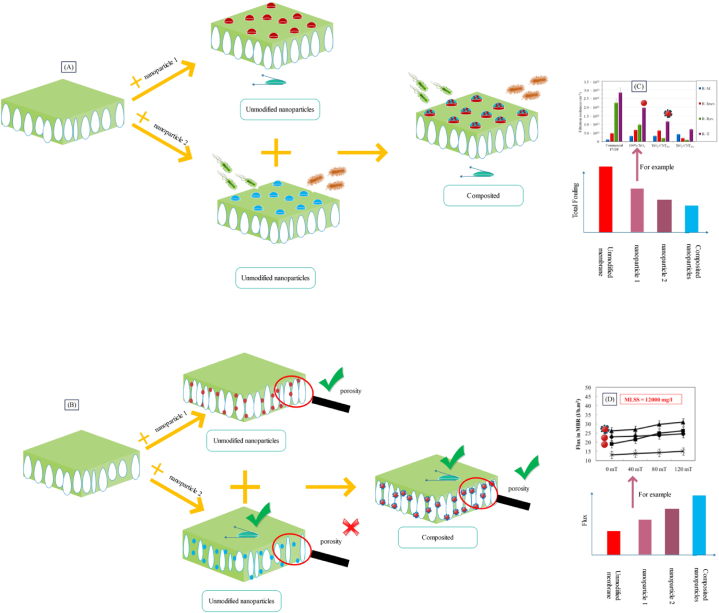
Modification of membrane with raw and composite nanoparticles: A: effect of surface modification on membrane fouling, B: impact of matrix modification on the membrane flux, C: Alteration of the filtration resistances of TFN membrane by adding composite nanoparticles to the surface layer (adapted from Ref. [177]), and D: Changes in the flux of MMM upon adding composite nanoparticles to the membrane matrix (adapted from Ref. [135]).

The modification methods of functionalization and coating result in enhanced performance of raw nanoparticles, while blending of two nanoparticles would confer new features to nanoparticles. Thus, usage of composite nanoparticles seems facilitate for benefiting from the positive features of both nanoparticles for the membrane. Indeed, nanoparticles have distinctive features such as antibacterial, hydrophilic, and magnetic properties. By employing composite nanoparticles within the membrane structure, the features of both nanoparticles can be harnessed concurrently, thereby improving the membrane performance in comparison to raw membrane or containing only one type of nanoparticles. Fig. 9 presents membrane modification with raw nanoparticles and modified nanoparticles via compositing method as well as the effect of this modification on the fouling and flux [50,[93], [133], [176]].

As observed in Fig. 9 (A), upon adding hydrophilic nanoparticles to the membrane surface, the membrane water contact angle falls, and usage of antibacterial nanoparticles leads to elimination of bacteria from the membrane surface. Ultimately, usage of both nanoparticles can contribute to reduced fouling of the membrane. Further, use of nanoparticles within the membrane structure results in enhanced membrane flux by augmenting hydrophilicity and porosity (Fig. 9 (B)). Fekete et al. [177] synthesized $\frac{{\text{TiO}}_{2}/\text{CNT}}{\text{PVDF}}M$ for separating oil in water emulsion. The results of this research indicated that the total filtration resistance for $\frac{{\text{TiO}}_{2}/\text{CNT}}{\text{PVDF}}M$, TiO2PVDFM, and PVDF membrane was found 0.75 × 10^11^, 1.95 × 10^12^, and 2.75 × 10^12^,respectively (Fig. 9 (C)). Elsewhere, for achieving high flux and enhancing cephalexin rejection from wastewater concurrently, Karimi et al. [62] employed iron oxide nanoparticles as porosity adjuster and zirconium oxide nanoparticles as an agent for improving hydrophilicity in the sublayer, plus zirconium and iron oxide nanoparticles (as core shell nanoparticles) in the top layer of membrane. $\frac{\text{PA}}{\text{PAN}}M$, $\frac{\text{PA}}{{\text{PAN}}^{\text{Fe}3O4}}M$, $\frac{\text{PA}}{{\text{PAN}}^{\text{ZrO}2}}M$, $\frac{{\text{PA}}^{\text{Fe}3O4/\text{ZrO}2}}{{\text{PAN}}^{\text{Fe}3O4}}M$, and $\frac{{\text{PA}}^{\text{Fe}3O4/\text{ZrO}2}}{{\text{PAN}}^{\text{ZrO}2}}M$ showed contact angles of 37, 28, 21, 21, and 18°, as well as fluxes of 53.49, 33.46, 40.62, 36, and 49.9 LMH, respectively. All indicate the positive effect of composite nanoparticles on the membrane structure and performance.

Noormohamadi et al. [135] synthesized ${\text{PAN}}^{\text{Fe}3O4/\text{ZrO}2}M$. For modifying the membrane structure, they applied iron oxide nanoparticles, because of magnetic properties, and zirconium oxide nanoparticles given their hydrophilic properties and lack of considerable increase in surface roughness. The filtration performance results indicated that the flux for ${\text{PAN}}^{\text{Fe}3O4/\text{ZrO}2}M$, ${\text{PAN}}^{\text{Fe}3O4}M$, ${\text{PAN}}^{\text{ZrO}2}M$, and PAN membrane was found 30.9, 26.1, 24.5, and 15 LMH (Fig. 9 (D)), while the filtration resistance for the mentioned membranes was found 10, 22, 15, and 55 % respectively.

**Fig. 10:**
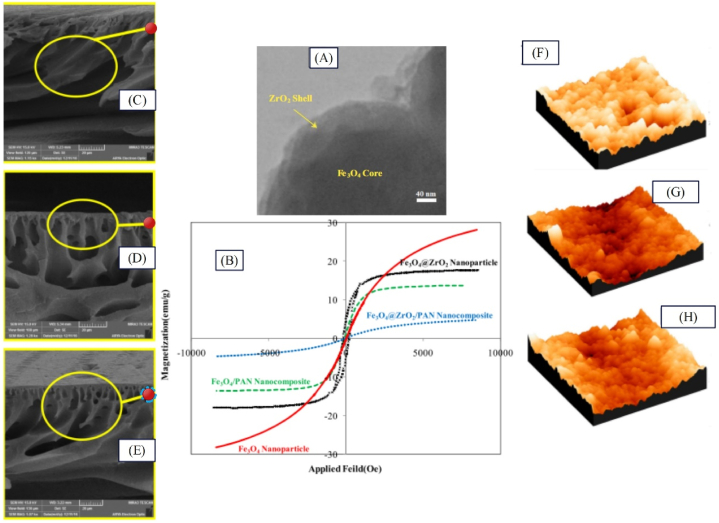
Results of the presence of composited nanostructures (ZrO_2_/Fe_3_O_4_) on the morphological and topological characteristics of ${\text{PAN}}^{\text{Fe}3O4/ZrO2}M$: A: TEM analysis of Fe_3_O_4_/ZrO_2_ nanoparticles, B: VSM analysis of Fe_3_O_4_ and Fe_3_O_4_/ZrO_2_ along with ${\text{PAN}}^{\text{Fe}3O4}M$ and ${\text{PAN}}^{\text{Fe}3O4/ZrO2}M$ , SEM images of C: ${\text{PAN}}^{\text{Fe}3O4}M$, D: ${\text{PAN}}^{\text{ZrO}2}M$, and E: ${\text{PAN}}^{\text{Fe}3O4/ZrO2}M$, AFM analysis of F: ${\text{PAN}}^{\text{Fe}3O4}M$, G: ${\text{PAN}}^{\text{ZrO}2}M$, H: and ${\text{PAN}}^{\text{Fe}3O4/ZrO2}M$ (adapted from Ref. [135]).

The TEM image (Fig. 10 (A)) demonstrates that the synthesized composite nanoparticles have a core-shell structure. The results related to VSM analysis reveal the magnetic properties of Fe_3_O_4_ and ZrO_2_/Fe_3_O_4_ nanoparticles as well as ${\text{PAN}}^{\text{Fe}3O4}M$ and ${\text{PAN}}^{\text{Fe}3O4/\text{ZrO}2}M$ (Fig. 10 (B)). VSM results indicated that the membrane containing composite nanoparticles (${\text{PAN}}^{\text{Fe}3O4/\text{ZrO}2}M$) has had a lower magnetic saturation than ${\text{PAN}}^{\text{Fe}3O4}M$, which can be due to the presence of zirconium oxide shell. As observed in the SEM images of nanocomposite membranes (Fig. 10 (C, D, E)), the membrane containing zirconium oxide nanoparticles has minimal porosity, while the porosity grows with the presence of iron oxide in the core-shell nanoparticles. According to AFM images (Fig. 10 (F, G, H)), the presence of zirconium shell in Fe_3_O_4_/ZrO_2_ nanoparticles makes the surface roughness of ${\text{PAN}}^{\text{Fe}3O4/\text{ZrO}2}M$ lower than that of ${\text{PAN}}^{\text{Fe}3O4}M$.

### 3.3 Efficiency of presence of nanostructures: what is the extent of improvement of membrane performance resulting from presence of nanostructure in separating pollutants from wastewater?

To investigate further details of application of these nanostructures regarding various fields of membrane processes, presence of modified nanostructures has been examined in separation of organic pollutants from wastewater, filtration of textile wastewater, separation of pharmaceutical pollutants from wastewater, separation of salt from wastewater, and improving the membrane performance in membrane bioreactors.

#### 3.3.1 The efficiency of presence of nanostructures in improving the membrane performance in separating organic pollutants from wastewater

**Fig. 11:**
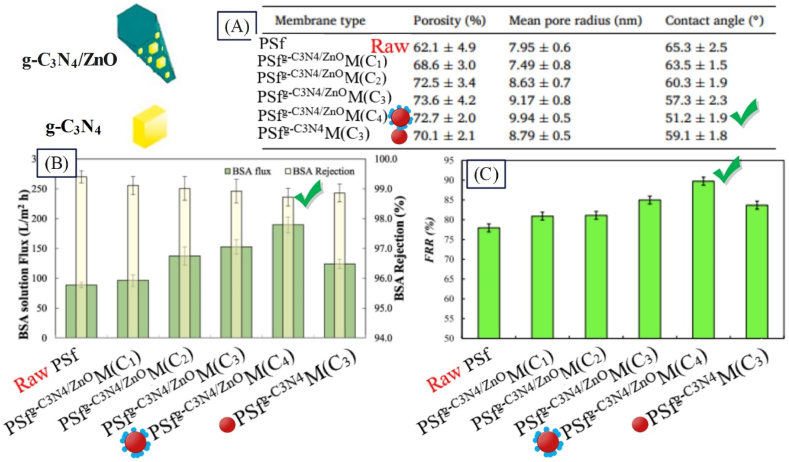
Performance results of a structurally modified membrane with composite nanoparticles (${\text{PSf}}^{g-C3N4/\mathrm{ZnO}}M$) in the separation of organic pollutants: A: porosity, contact angle and mean pore size analysis; B: BSA solution flux and rejection; and C: Flux recovery ratio (FRR) (adapted from Ref. [178]).

Extensive studies have been done on use of nanoparticles within the membrane structure. In these studies, different polymers and various nanostructures have been added to the membrane structure. The synthesized membranes have been used for removal of different pollutants with different concentrations. According to results, presence of nanostructures affects the flux and extent of removal of pollutants. Vatanpour et al. [178] synthesized ${\text{PSf}}^{g-C3N4/\text{ZnO}}M$ for separating organic compounds. According to Fig. 11 (A), ${\text{PSf}}^{g-C3N4/\text{ZnO}}M$ containing g-C_3_N_4_/ZnO composite nanostructure had a lower contact angle than ${\text{PSf}}^{g-C3N4}M$ containing g-C_3_N_4_ nanostructure, due to higher hydrophilicity g-C_3_N_4_/ZnO resulting from the presence of –OH groups within the ZnO structure. Also, according to Fig. 11 (B), the separation percentage for all membranes is higher than 98 % and at the optimal concentration (0.5 wt%), ${\text{PSf}}^{g-C3N4/\text{ZnO}}M$ has the highest flux. Meanwhile, according to Fig. 11 (C), the antifouling performance of ${\text{PSf}}^{g-C3N4/\text{ZnO}}M$ is higher than that of the raw membrane and ${\text{PSf}}^{g-C3N4}M$.

Zhou et al. [87] employed ${\text{PVDF}/\text{PFSA}}^{\text{SiO}2}$ M containing unmodified nanoparticles for separating bovine serum albumin (BSA). They found that, due to the reduction in contact angle from 93.7 to 63.5, presence of unmodified nanoparticles improved the pure water flux by 1.8 % and BSA separation by 1.5 %. Also, with the reduction of roughness, the flux decline ratio reached 17.4 %. Yu et al. [100] employed ${\text{PVDF}}^{\text{SiO}2}M$ containing SiO_2_ unmodified nanoparticles for BSA. They found that presence of SiO_2_ improved pure water flux by 150 % and lowered rejection by 3 % due to the reduction in contact angle from 82.9 to 53.4 and the increase in porosity from 52.5 to 84.2. Through functionalized nanoparticles in ${\text{PSf}}^{\text{NH}2‐\text{SiO}2}M$ for BSA separation, the resulting membrane could enhance the pure water flux by 700 %, and improve the separation by 10 % [87]. Considering the diversity of studies, employed particles, and their concentrations, it is not possible to offer an absolute judgment about the efficiency of membranes. Accordingly, IFP parameter was calculated based on Relation (3), with its results presented in Table 3. Higher values of this parameter indicate greater membrane efficiency and greater influence of the modification applied.

**Table 3:** Examining the influence of presence of nanoparticles on the performance of nanocomposite membranes in separation of organic pollutants from wastewater.

Row	Nanocomposite Membrane Composition		Filtration condition		Filtration performance		IFP for organic pollutant separationn	Reference
	Membrane name	NS/Polymer Conc. (wt.%)	Pollutant Type	Pollutant Conc.	Rejection Enhancement	Pure Water Flux Enhancement	$\frac{\mathrm{PWF}\;\mathrm{enhancement}}{\mathrm{Consumed}\;\mathrm{NP}}$	
1	${\text{PVDF}}^{\text{TiO}2}M$	2.5	BSA	1000	+18	−16.7	**−6.7**	[86]
2	${\text{PVDF}/\text{PFSA}}^{\text{SiO}2}M$^a^	16.8	BSA	1000	+1.5	+1.8	**0.1**	[87]
3	${\text{PVDF}}^{\text{TiO}2}M$	62	BSA	–	0	+26	**0.41**	[88]
4	${\text{PES}}^{\text{TiO}2}M$	37	Protein	–	+2	+20	**0.54**	[37]
5	${\text{PSf}}^{\text{SiO}2}M$	10	Oil	65	+0.3	+8	**0.8**	[81]
	${\text{PSf}}^{\text{Ce}\_\text{SiO}2}M$		Oil	65	+0.6	+13	**1.3**	
6	${\text{PES}}^{\text{TiO}2}M$	26.7	N/A	–	–	+29.3	**1.1**	[89]
7	${\text{PS}}^{\text{TiO}2}M$^b^	51	BSA	100	+72	+100	**1.9**	[90]
8	${\text{PVA}}^{\text{SiO}2}M$	14	Dye	60	+19	+40	**2.85**	[91]
9	${\text{PES}}^{\text{SiO}2-\text{Ag}}M$	11	BSA	1000	0	+32	**2.9**	[92]
10	${\text{PVDF}}^{\text{TiO}2/\text{ZrO}2}M$	15	N/A	–	–	+49	**3.2**	[93]
	${\text{PVDF}}^{\text{SiO}2}M$					+15	**1**	
11	${\text{PVDF}}^{\text{SiO}2}M$	30	Dextran-40	1000	−28.5	+100	**3.3**	[94]
12	${\text{PVDF}}^{\text{TiO}2}M$	20	BSA	1000	+23	+100	**5**	[95]
13	${\text{CA}}^{\text{TiO}2}M$	19	BSA	1000	+15	+104	**5.4**	[96]
14	${\text{PSf}}^{\text{ZrO}2}M$	5	N/A	–	–	+35	**7**	[97]
15	${\text{EPVC}}^{\text{TiO}2}M$^c^	13.4	BSA	500	+8	+100	**7.46**	[98]
16	${\text{PVDF}}^{Mg-Al\;}M$	3.3	BSA	100	+8.5	+25	**7.57**	[99]
17	${\text{PES}}^{\text{TiO}2}M$	11.1	BSA	1000	+2.6	+18	**1.6**	[77]
	${\text{PES}}^{\text{Sulfate}-\text{TiO}2}M$		BSA		+2.9	+78	**7**	
18	${\text{PVDF}}^{\text{SiO}2}M$	16	BSA	300	−3	+150	**9.3**	[100]
19	${\text{PVDF}}^{\text{ZnO}}M$	6.7	N/A	–	–	+63	**9.4**	[101]
20	${\text{CA}}^{\text{TiO}2}M$	57	BSA	800	+3	+550	**9.6**	[102]
21	${\text{PES}}^{\text{ZrO}2}M$	5	PEO	–	0	+55	**11**	[103]
22	${\text{PVC}}^{\text{SiO}2}M$^d^	1.5	PEO	–	0	+17	**11.33**	[104]
23	${\text{PVDF}}^{\text{polyhexanide}-\text{CuO}}M$	20.6	BSA	1000	+177	237	**11.5**	[105]
24	${\text{CA}}^{\text{NH}2-\text{SiC}}M$^e^	5.5	Oil	25000	+22.6	+69	**12.58**	[106]
25	${\text{PVDF}}^{\text{SnO}2/\text{GO}}M$	6.25	BSA	1000	+52	+80.5	**12.88**	[85]
26	${\text{PES}}^{\text{APTES}-\text{TiO}2}M$^f^	12.5	Dextran	–	−4	+28.5	**14.8**	[107]
27	${\text{PES}}^{\text{Sulfonate}-\text{TiO}2}M$	6.7	BSA	500	+10.7	100	**14.9**	[43]
28	${\text{PES}}^{\text{ZnFe}2O4/\text{SiO}2}M$	2.5	N/A	–	–	+38	**15.2**	[108]
29	${\text{PES}}^{\text{PVP}-\text{SiO}2}M$	1	BSA	1000	−4	+18	**18**	[109]
30	${\text{PVC}}^{\text{Fe}3O4/O-\text{MWCNT}}M$	4.16	BSA	1000	+117	+80	**19.23**	[110]
31	${\text{PSf}}^{\text{NH}2-\text{SiO}2}M$	33	BSA	500	+10	+700	**21.2**	[111]
32	${\text{PES}}^{\text{COOOH}-\text{Fe}3O4/\text{SiO}2}M$	2.5	BSA	200	0	+76.6	**31.42**	[112]
33	${\text{PES}}^{\text{CuO}/\text{ZnO}}M$	1.1	N/A	–	–	+33	**30**	[113]
34	${\text{PES}}^{\text{PDA}-\text{ZnFe}2O4}M$	4	Oil	500	+92	+188	**47**	[114]
35	${\text{PSf}}^{\text{COOH}-\text{CNT}}M$	0.77	Oil	1000	+16.28	+43.5	**56.47**	[115]
36	${\text{PES}}^{\text{NH}2-\text{PDA}-\text{Fe}3O4}M$	0.5	BSA	1000	+28	+66.13	**132.27**	[116]
	${\text{PES}}^{\text{Sulf}-\text{PDA}-\text{Fe}3O4}M$				+22.9	+57.23	**114.48**	

a SPSF: Sulfonated polysulfone.

b PS: Polystyrene.

c EPVC: Emulsion poly (vinyl chloride).

d PVC: Poly (vinyl chloride).

e SiC: Silicon carbide.

f APTES: 3-aminopropyltriethoxysilane.

Regarding use of modified nanoparticles, for BSA separation, Guo and Kim [77] utilized ${\text{PES}}^{\text{TiO}2}M$ and ${\text{PES}}^{\text{Sulfate}‐\text{TiO}2}M$, whereby the extent of flux augmentation compared to the raw membrane was found 18 and 78 % respectively. In another research, Zhou et al. [87] used ${\text{PSf}}^{\text{NH}2‐\text{SiO}2}M$ for BSA separation, whereby IFP was found 21.2. In another research for oil separation, Kallem et al. [114]. employed ${\text{PES}}^{\text{PDA}‐\text{Fe}3O4}M$, whereby the IFP parameter was found as 47. As observed in Table 3, IFP parameter has been lower than 11.5 in studies in which unmodified nanoparticles have been used, while with modification of nanoparticles, the value of this parameter has grown to 132.27 for ${\text{PES}}^{{\text{NH}}_{2}‐\text{PDA}‐\text{Fe}3O4}M$ [116].

#### 3.3.2 Efficiency of presence of nanostructures in improving the membrane performance in separating dyes from wastewater

**Fig. 12:**
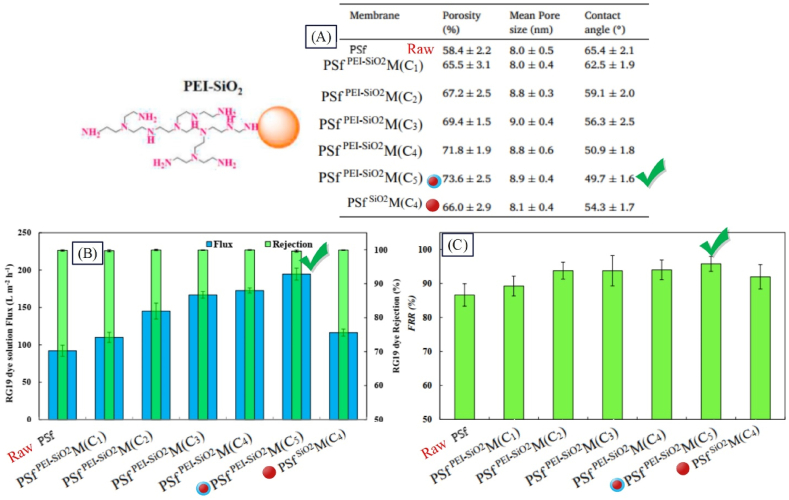
Performance results of a structurally modified membrane with coated nanoparticles (${\text{PSf}}^{\mathrm{PEI}-SiO2}M$**)** in separating dyes from textile wastewater: A: Porosity, water contact angle, and mean pore size; B: Reactive Green 19 dye solution flux and rejection; and C: FRR (adapted from Ref. [125]). (For interpretation of the references to colour in this figure legend, the reader is referred to the Web version of this article.)

Newly emerging dye pollutants are considered a major threat for the environment, since many textile industries generate a large volume of such heavily contaminated wastes. Without the proper treatment, discharge of textile wastes causes major concerns for the environment [119]. One of the commonly used methods for dye separation is nanocomposite membranes. Extensive studies have been performed on use of nanostructures within the membrane structure for removing dyes. In these studies, different polymers and nanostructures have been added to the membrane structure. The synthesized membranes have been employed for removal of different dyes at different concentrations. The results have shown that presence of nanostructures affects the flux and extent of dye removal. For instance, Vatanpour et al. [125] synthesized ${\text{PSf}}^{\text{PEI}-\text{SiO}2}M$ for dye separation from textile wastewater. According to Fig. 12 (A), adding SiO_2_ nanoparticle to the raw PSf membrane has resulted in a decline in the contact angle, and upon coating the nanoparticle, the extent of hydrophilicity has increased. So that ${\text{PSf}}^{\text{PEI}-\text{SiO}2}M$ with a contact angle of 50.9° has been more hydrophilic than ${\text{PSf}}^{\text{SiO}2}M$ with a contact angle of 54.3°. According to Fig. 12 (B), the separation of RG19 dye through all membranes is about 100 %. Due to the increase in hydrophilicity and porosity because of the presence of PEI-SiO_2_ coated nanoparticles, the flux rose from about 92 LMH for the raw membrane to about 195 LMH for ${\text{PSf}}^{\text{PEI}-\text{SiO}2}M$; The flux of the solution containing the pollutant has grown by about 65 % and the antifouling performance has also improved as a result of reducing the adhesion of organic pollutants such as protein and dyes to the surface (according to Fig. 12 (C)).

Zinadini et al. [117] utilized PES^GO^M for separation of Red 16 dye. They found that the resulting membrane could remove 96 % of dye from wastewater due to a decrease in the contact angle from 65.2 to 53.2. Also, the flux of the synthesized membrane was observed as 20.4 LMH, showing 148.7 % increase compared to the raw membrane. Akbari and Homayoonfal [22] employed ${\text{PSf}}^{\text{TiO}2}M$ for separating the Yellow 12 dye. They observed that the resulting membranes could separate 97 % of the dye from wastewater, indicating 4.2 % enhancement compared to the raw membrane, and the flux of the synthesized membrane was achieved 34 LMH. In another study by Yang et al. [121], PMIA^GO^M was utilized for separating Red, Red x-GTL, and Yellow dyes. The results revealed that the obtained membrane through a reduction in the contact angle from 76 to 56 could eliminate 95, 98, and 94 % of the mentioned dyes from wastewater, and the flux and FRR of the synthesized membrane reached 125.2 LMH and 98.7, revealing 159 % flux increase compared to the raw membrane. Considering the diversity of the examined results, IFP parameter was calculated according to Relation (4) for more precise investigation of studies. The results with further details are reported in Table 4.

**Table 4:** Investigating the effect of presence of nanoparticles on the performance of nanocomposite membranes in separating dyes from textile wastewater.

Row	Nanocomposite membrane composition		Filtration condition		Filtration performance		IFP for dye separation	Reference
	Membrane name	NS/Polymer Conc. (wt.%)	Dye name	Dye Conc. (g/L)	Water flux (LMH)	Dye removal (%)	$\frac{\text{Flux}\times \mathrm{Dye}\;\mathrm{Conc}}{\Delta P\times \mathrm{Consumed}\;\mathrm{NP}}$	
1	${\text{PES}}^{\text{GO}}M$	2.5	Red16	0.03	20.4	96	**0.061**	[117]
2	${\text{PES}}^{\text{FeON}}M$	26.66	Congo red	0.2	66	89.8	**0.179**	[118]
3	${\text{PES}}^{\text{CeO}2}M$	10	Congo red	0.1	131	9936	**0.262**	[119]
4	${\text{PES}}^{\text{PABFNP}}M$	1.25	red 16	0.03	64.2	99	**0.385**	[120]
5	${\text{PSf}}^{\text{TiO}2}M$	2	Yellow 12	0.1	34	97	**0.425**	[22]
6	${\text{PMIA}}^{\text{GO}}M$	1.5	Red x-GTL	0.05	125.2	98	**0.522**	[121]
			Red	0.05	125.2	95		
			Yellow	0.05	125.2	94		
7	${\text{PES}}^{\text{ZnO}/\text{MWCNT}}M$	2.5	Red16	0.5	16.7	94	**0.8.35**	[122]
8	${\text{PES}}^{F-\text{carbon}\;\text{dots}}M$	2.38	Red 198	0.1	76.5	98.9	**1.071**	[123]
9	${\text{PES}}^{\text{HMDI}-\text{TiO}2}M$^a^	4	Congo red	0.024	52	97.4	**1.585**	[124]
10	${\text{GO}}^{\text{PAA}-\text{NH}2-\text{UiO}-66}M$	2	Congo red	0.02	150	98	**1.667**	[79]
11	${\text{PES}}^{\text{MDA}-\text{Fe}3O4}M$	2.38	Green 19	0.1	185.7	98	**2.601**	[75]
12	${\text{PSf}}^{\text{PEI}-\text{SiO}2}M$	2	RG19	0.1	195	100	**3.25**	[125]
13	$\frac{{\text{PDA}}^{\text{PEI}-\text{TiO}2\_\text{Ag}\;}}{\text{PAN}}M$	–	black 5	0.2	81.2	99.5	**-**	[126]
14	$\frac{{\text{PA}}^{\text{GO}\_\text{TiO}2}}{\text{PSf}}M$	0.1	Green19	0.1	61	99.4	**6.1**	[127]
		0.1	Yellow12	0.1	61	95.4		
		0.1	Blue 21	0.1	61	81.4		
15	${\text{PSf}}^{\text{ZrO}2}M$	2	Yellow 4GNL	0.1	500	98	**8.333**	[31]
	${\text{PSf}}^{\text{COOH}-\text{ZrO}2}M$	2		0.1	520	97	**8.667**	
	${\text{PSf}}^{\text{SO}4-\text{ZrO}2}$ M	2		0.1	550	98	**9.167**	
16	${\text{PAN}}^{\text{Fe}3O4/\text{ZrO}2}M$	1	Yellow 4GNL	0.1	450	90	**15**	[49]

a HMDI: 1, 6-hexamethylene diisocyanate.

In ${\text{PSf}}^{\text{TiO}2}M$ [22], ${\text{PES}}^{\text{HMDI}‐\text{TiO}2}M$ [124], and $\frac{{\text{PDA}}^{\text{PEI}‐\text{TiO}2/\text{Ag}\;}}{\text{PAN}}M$ [126] membranes, TiO_2_ nanoparticles have been used as raw, modified via functionalization, and modified through coating and compositing methods respectively, and the flux of the mentioned membranes has been reported 34, 52, and 81.2 LMH respectively, indicating the positive effect of nanoparticles modification. Koulivand et al. [123] synthesized PES^F-carbon dots^ M by functionalizing carbon dots nanostructure with using sulfone, -SO_2_OH, and COOH groups and by adding it to the membrane structure for separating Red 198 dye, IFP was calculated as 1.071. In another research, Safarpour et al. [127] utilized $\frac{{\text{PA}}^{\text{GO}‐\text{TiO}2}}{\text{PSf}}M$, where IFP was calculated 6.1. As observed in Table 4, with modification of nanoparticles, the IFP parameter increased; in the examined studies, this parameter has had a value of lower than 0.6 for unmodified nanostructures, while soaring to 15 with the nanoparticle modification for ${\text{PAN}}^{\text{Fe}3O4/\text{ZrO}2}M$ [49].

#### 3.3.3 Efficiency of presence of nanostructures in improving the membrane performance in membrane bioreactors

**Fig. 13:**
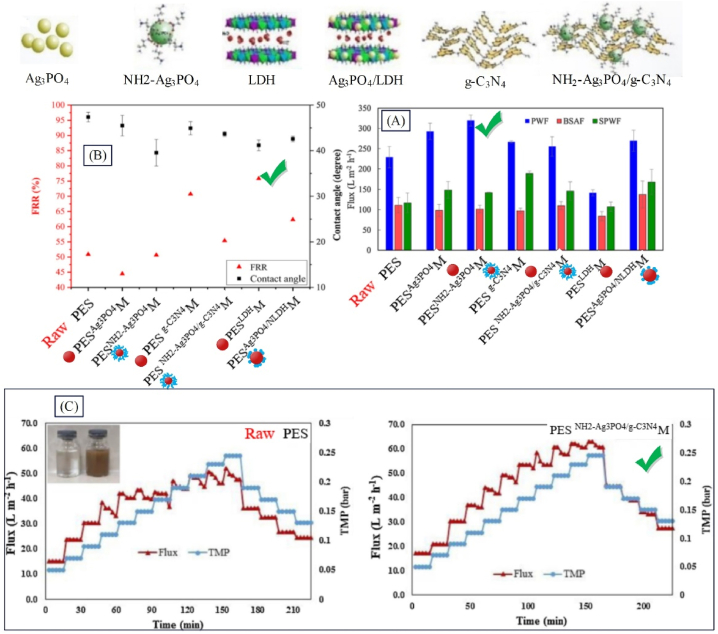
Performance results of a structurally modified membrane with modified nanoparticles (${\text{PES}}^{NH2-Ag3PO4/g-C3N4}M$) in membrane bioreactor: A: Water flux, BSA flux, and second water flux after protein filtration; B: Flux recovery ratios and the contact angle; C: The critical flux of raw and nanocomposite membrane in MBR (adapted from Ref. [134]).

Membrane bioreactor (MBR) is widely used as a water treatment process in treating household and industrial wastewater such as petrochemical wastewater, treating disinfectants-containing wastewater, leachate treatment, etc. Nevertheless, the membrane fouling which leads to shortening of the lifespan and high energy consumption of MBR is considered as the main stumbling block in development of MBR [179]. From among all methods of modification in membrane preparation, nanocomposite membranes have shown a promising performance, offering intrinsic properties of polymer membranes plus nanostructure, and presenting interesting advantages including anti-fouling and good separation performance as well as excellent compatibility with tough operational conditions [180]. Many studies have been performed on addition of nanostructures to the membrane structure of membrane bioreactors. Various studies were investigated in terms type of polymer, type of nanostructure, nanostructures concentration, and method of adding nanostructure to the membrane structure. In these studies, the conditions of membrane bioreactors including solid retention time (SRT), hydraulic retention time (HRT) and MLSS were compared as well as the membrane performance indicators including COD removal, filtration resistance, flux recovery ratio, and flux. The conclusion of the results is presented in Table 5. Based on investigations, Ghalamchi et al. [134] synthesized ${\text{PES}}^{g-C3N4}M$, ${\text{PES}}^{\text{Ag}3\text{PO}4}M$, ${\text{PES}}^{\text{NH}2-\text{Ag}3\text{PO}4}M$, ${\text{PES}}^{\text{NH}2-\text{Ag}3\text{PO}4/g-C3N4}M$, ${\text{PES}}^{\text{Ag}3\text{PO}4/\text{NLDH}}M$, and ${\text{PES}}^{\text{LDH}}M$ membranes to be applied in MBR. As observed in Fig. 13 (B), the hydrophilicity of the nanocomposite membranes was improved by adding inorganic, organic, and nanocomposite materials compared to the raw membrane. Modifying nanoparticles with amine groups and enhancing hydrophilicity caused the water flux of ${\text{PES}}^{\text{NH}2-\text{Ag}3\text{PO}4}M$ to be 9 % higher than that of ${\text{PES}}^{\text{Ag}3\text{PO}4}M$ (Fig. 13 (A)). Hydrophilic nanosheets within the polymer matrix facilitate the creation of hydrogen bonds with water molecules and by developing a thin layer of water molecules on the membranes, they prevent the absorption of BSA molecules on the surface of the membranes. Thus, the high values of FRR belong to the membranes containing nanosheets reaching 70.7 and 75.8 % for ${\text{PES}}^{g-C3N4}M$ and ${\text{PES}}^{\text{NLDH}}M$, respectively (Fig. 13 (B)). The critical flux of the raw membrane in MBR was 43 LMH, which suddenly dropped due to faster and severe membrane fouling while the antibacterial and hydrophilic properties of the ${\text{PES}}^{\text{NH}2-\text{Ag}3\text{PO}4/g-C3N4}M$ led to a reduction in the interaction between the foulant and the membrane surface, thus improving the critical flux (see Fig. 13 (C)).

Homayoonfal et al. [130] synthesized ${\text{PSF}}^{\text{Al}2O3}M$. They found that the flux was 12 LMH, showing 200 % increase compared to raw membrane due to a decline in the contact angle from 72 to 51. Amini et al. [132] synthesized ${\text{HDPE}}^{\text{SiO}2}$ M, reporting that due to a reduction in the contact angle from 116.2 to 97.2 and an increase in porosity from 61 to 69, COD removal, flux recovery, and membrane flux reached 96 %, 63 %, and 18 LMH respectively. Specifically, it showed 80 % increase in flux compared to the raw membrane. Also, in another research by Hashemi et al. [131], due to a reduction in the contact angle from 57 to 46 and an increase in porosity from 39 to 49, the removal percentage of COD and flux of the ${\text{PAN}}^{\text{Al}2O3}M$ synthesized membrane were reported 97 and 20 LMH respectively, indicating 300 % increase of flux compared to the raw membrane. Given the variety of the concentration of employed nanostructures and the varying operational conditions of membrane bioreactors, IFP parameter was defined and calculated according to Relation (5). The results have been presented in Table 5. Higher values of this parameter would indicate better efficiency of the membrane and greater impact of modification.

**Table 5:** Exploring the effect of presence of nanoparticles on the performance of nanocomposite membranes in membrane bioreactor.

Row	Nanocomposite membrane composition		Filtration condition				Filtration performance				IFP for MBR separation	Reference
	Membrane Name	NS/Polymer Conc. (wt.%)	MLSS (Kg/L)	HRT (h)	SRT (day)	Pressure (bar)	Flux (LMH)	Flux Recovery (%)	Filtration Resistance (%)	COD Removal (%)	$\frac{\mathrm{Flux}\times \mathrm{MLSS}}{\Delta P\times \mathrm{Consumed}\;\mathrm{NP}}$	
1	${\text{PES}}^{\text{GO}}M$	3.3	10	6	∞	3	21	92.8	26.2	94.7	**0.024**	[128]
2	${\text{PVDF}}^{\text{TiO}2}M$	5	2.5	12	25	0.4	35.28	–	39	–	**0.044**	[129]
3	${\text{PSF}}^{\text{Al}2O3}M$	3	12	–	∞	0.4	12	–	87	90.28	**0.12**	[130]
4	${\text{PAN}}^{\text{Al}2O3}M$	2	8.5	–	–	0.3	20	91	57	88.71	**0.283**	[131]
5	${\text{HDPE}}^{\text{SiO}2}M$	0.5	8	24	20	0.4	20	89.92	52.5	96.7	**0.8**	[132]
6	${\text{PVDF}}^{\text{Ag}/\text{SiO}2}M$	3.5	7	12	20	0.2	–	75	–	94.5	**-**	[133]
7	${\text{PES}}^{\text{NH}2-\text{Ag}3\text{PO}4}M$	3.125	6	–	–	0.5	242	55	–	–	**0.929**	[134]
8	${\text{PAN}}^{\text{Fe}3O4}M$	1	10	8	∞	0.3	43	80	22	91	**1.433**	[135]
	${\text{PAN}}^{\text{ZrO}2}M$	1	10			0.3	35	70	15	90	**1.167**	
	${\text{PAN}}^{\text{Fe}3O4/\text{ZrO}2}$ M	1	10			0.3	47	92	10	94	**1.567**	
9	${\text{PVDF}}^{\text{OCMCS}/\text{Fe}3O4}M$^a^	0.36	10	26	∞	2	125.8	95	–	97	**1.747**	[136]
10	${\text{PES}}^{\text{PVP}-\text{DDT}-\text{Ag}}$ M^b^	2	7.32	24	20	0.1	50	94.26	10.84	98.4	**1.83**	[137]
11	${\text{CA}}^{\text{NH}2-\text{ND}}$ M	2.85	6.75	24	25	0.1	77.8	95	83	91	**1.843**	[76]
12	${\text{PSf}}^{S-\text{ND}}M$	1	8	24	25	0.1	40	58.94	–	–	**3.2**	[138]
13	${\text{PVDF}}^{\text{OCMCS}/\text{Fe}3O4}M$	0.05	1	–	∞	1	250	95	72	–	**5**	[139]

a OCMCS: O-carboxymethyl chitosan.

b DDT: 1-dodecanthiol.

Regarding use of modified nanostructure within the structure of membrane of MBRs, Etemadi et al. [76] used functionalized nanoparticles in ${\text{CA}}^{\text{NH}2‐\text{ND}}M$, whereby the IFP value was found 1.873. In another research, Tizchang et al. [138] utilized ${\text{PSf}}^{S‐\text{ND}}M$ in membrane bioreactor, with IFP found as 3.2. As observed in Table 5, across all of the examined studies, when raw nanostructures were used, the value of this parameter has been lower than 1.5, and modification on the nanostructure would lead to enhancement of this parameter. For example, for the ${\text{PVDF}}^{\text{OCMCS}/\text{Fe}3O4}M$, this parameter was calculated 5 [139].

#### 3.3.4 Efficiency of presence of nanostructures in improving the membrane performance in separating salts from the wastewater

**Fig. 14:**
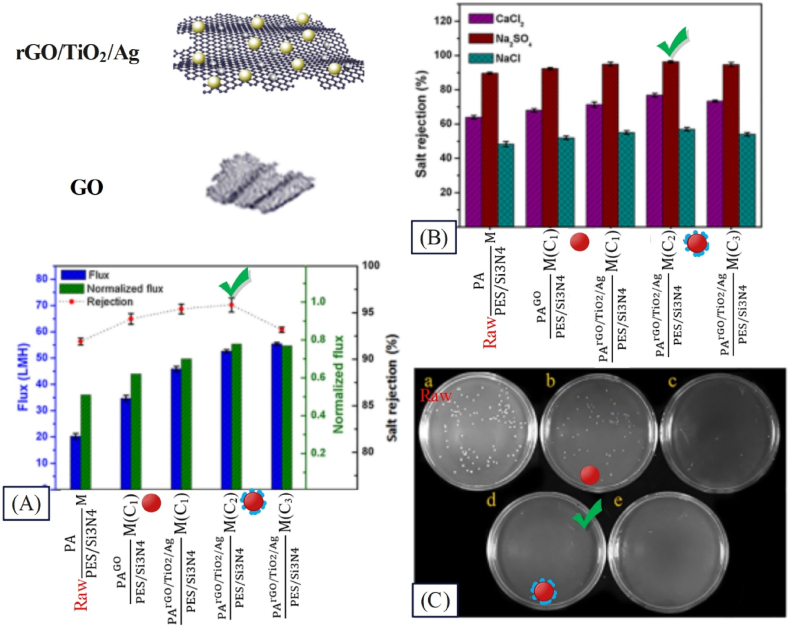
Performance results of a surface modified membrane with composite nanoparticles ($\frac{{\text{PA}}^{\mathrm{rGO}/TiO2/\mathrm{Ag}}}{\mathrm{PES}/Si3N4}M$) in the separation of salt from wastewater: A: flux, normalized flux, and salt rejection; B: salt rejection with CaCl_2_, Na_2_SO_4_, and NaCl feed solutions; C: antibacterial properties of the membranes (adapted from Ref. [149]).

The general water crisis especially freshwater has been one of the most serious challenges of recent decades. Today desalination process is finding a great status against such challenges [143]. Membrane separation method can play a vital role in treating drinking water, desalination of brine wastewater as well as seawater thanks to their numerous advantages such as low cost, low process temperature, lack of byproducts, and high separation efficiency [140]. Usage of nanostructures within the membrane structure would enhance its performance. Numerous studies have been done on addition of nanostructures within the membrane structure for salt separation. Various studies have been reviewed and investigated regarding the composition of the composite membrane including type of polymer, type of nanostructure, and concentration of nanostructure, with Table 6 summarizing the results. The results show that presence of nanostructure affects the flux and extent of salt removal. Abadikhah et al. [149] synthesized $\frac{{\text{PA}}^{\text{rGO}/\text{TiO}2/\text{Ag}}}{\text{PES}/\text{Si}3N4}M$ for desalination. Based on the results presented in Fig. 14 (A), the $\frac{{\text{PA}}^{\text{rGO}/\text{TiO}2/\text{Ag}}}{\text{PES}/\text{Si}3N4}M$ shows a 70 % increase in water flux compared to $\frac{\text{PA}}{\text{PES}/\text{Si}3N4}M$. This observation is due to the addition of nanostructures in the surface layer of PA, which can create additional pathways at the interface of nanoparticles and polymer matrix as well as facilitate water penetration. Also, $\frac{{\text{PA}}^{\text{rGO}/\text{TiO}2/\text{Ag}}}{\text{PES}/\text{Si}3N4}M$ presented a 48 % increase in water flux compared to $\frac{{\text{PA}}^{\text{GO}}}{\text{PES}/\text{Si}3N4}M$, which can be explained by the addition of rGO/TiO_2_/Ag hydrophilic composite nanostructures in the surface layer of PA. The increase in the normalized flux values in $\frac{{\text{PA}}^{\text{rGO}/\text{TiO}2/\text{Ag}}}{\text{PES}/\text{Si}3N4}M$ indicates that the addition of nanocomposite nanostructures has improved the antifouling properties. According to Fig. 14 (B), the best desalination performance is related to $\frac{{\text{PA}}^{\text{rGO}/\text{TiO}2/\text{Ag}}}{\text{PES}/\text{Si}3N4}M$ at the optimal concentration of composite nanostructure (0.2 wt%). As observed in Fig. 14 (C), by modifying the GO nanosheet with TiO_2_ and Ag nanoparticles, the antibacterial performance of the membranes has also improved.

Emadzadeh et al. [141] employed $\frac{\text{PA}}{{\text{PSf}}^{\text{TiO}2}}M$ for NaCl separation. The findings revealed that the resulting membranes could separate 93 % of salt from wastewater through the decrease in the contact angle from 71 to 64 and the increase of porosity from 71 to 75, where the flux of the synthesized membrane was found 4.9 LMH, indicating 64.7 % increase compared to the $\frac{\text{PA}}{\text{PSF}}$ membrane. Agbaje et al. [142] utilized ${\text{PVDF}}^{\text{Fe}3O4}M$ for separating NaCl salt. The results indicated that the obtained membranes could separate 99.5 % of salt from wastewater, and the flux of the synthesized membrane was observed 15 LMH, being 15 % greater compared to the raw membrane flux. In another study by Lakhotia et al. [140], $\frac{{\text{PA}}^{\text{FeO}}}{\text{PES}}M$ was utilized for separating MgCl_2_ salt. They found that the resulting membrane could separate 98.92 % of salt from wastewater with a decline in the contact angle from 76.1 to 49.6, and the flux of the synthesized membrane was observed 23.88 LMH, showing 36 % increase compared to raw membrane. Given the diversity of the examined results, the IFP parameter, obtained by Relation (6), was calculated for more precise investigation of studies. The results with greater details are provided in Table 6.

**Table 6:** Examining the effect of presence of nanoparticles on the performance of nanocomposite membranes in separating salt from wastewater.

Row	Nanocomposite membrane composition		Filtration condition		Filtration performance		IFP for salt separation	Reference
	Membrane name	NS/Polymer Conc. (wt.%)	Salt name	Salt Conc. (g/L)	Water flux (LMH)	Salt removal (%)	$\frac{\text{Flux}\times \mathrm{Salt}\;\mathrm{Conc}}{\Delta P\times \mathrm{Consumed}\;\mathrm{NP}}$	
1	$\frac{{\text{PA}}^{\text{FeO}}}{\text{PES}}M$	10	MgCl_2_	2	23.88	98.92	**0.46**	[140]
2	$\frac{\text{PA}}{{\text{PSf}}^{\text{TiO}2}}M$	2.855	NaCl	1.1688	4.9	73	**0.8**	[141]
3	${\text{PVDF}}^{\text{Fe}3O4}M$	10	NaCl	1	15	99.5	**0.75**	[142]
4	${\text{CA}}^{\text{ZnO}}$ M	3.57	NaCl	1	26.57	99	**1.24**	[143]
5	$\frac{{\text{PA}}^{\text{SiO}2}}{\text{PSf}}M$	5	NaCl	0.5844	36.5	78	**1.42**	[144]
6	${\text{PES}}^{\text{PVP}-\text{Fe}3O4}M$	0.55	Na_2_SO_4_	1	6.6	90	**2.4**	[46]
7	$\frac{{\text{PA}}^{\text{Fe}3O4/\text{ZnO}}}{{\text{PSE}}^{\text{Fe}3O4/\text{ZnO}}}M$	1.42	NaCl	1.1688	16.5	96.5	**5.43**	[71]
8	${\text{PES}}^{\text{NH}2‐\text{Fe}3O4/\text{SiO}2}M$	2.5	Na_2_SO_4_	1	65	99	**6.5**	[145]
9	$\frac{{\text{PDA}}^{\text{TiO}2/\text{ZnO}}}{\text{PA}}M$	1	MgSO_4_	1	28.4	99	**7.1**	[146]
10	$\frac{{\text{PA}}^{\text{GO}‐\text{SiO}2}}{\text{PSF}}M$	0.5	Na2SO_4_	2	43	93.2	**24.57**	[147]
11	$\frac{\text{PA}}{{\text{PES}}^{\text{NH}2‐\text{ZnO}}}M$	0.3	NaCl	2.33	10.225	93.08	**31.77**	[148]
12	$\frac{{\text{PA}}^{\text{rGO}/\text{TiO}2/\text{Ag}}}{\text{PES}/\text{Si}3N4}M$	0.2	MgSO_4_	1	52	95	**37.1**	[149]
13	${\text{PVDF}}^{\text{CMC}-\text{ZnO}}M$^a^	0.33	Na_2_SO_4_	1	139.788	95.01	**423.6**	[150]

a CMC: Carboxymethyl chitosan.

In ${\text{PVDF}}^{\text{Fe}3O4}M$ [142], ${\text{PES}}^{\text{PVP}‐\text{Fe}3O4}M$ [46], $\frac{{\text{PA}}^{\text{Fe}3O4/\text{ZnO}}}{{\text{PSE}}^{\text{Fe}3O4/\text{ZnO}}}M$ [71], and ${\text{PES}}^{\text{NH}2‐\text{Fe}3O4/\text{SiO}2}M$ [145] membranes, Fe_3_O_4_ nanoparticles have been used as raw, modified via coating method, modified through compositing method, and use in top layer and sublayer, as well as modified through compositing and functionalization methods, respectively. The IFP parameter of the mentioned membranes has been reported 0.75, 2.4, 5.43, and 6.5 respectively, indicating the positive effect of nanoparticle modification. In another research, Darabi et al. [148] used amine groups for functionalizing ZnO nanoparticles in $\frac{\text{PA}}{{\text{PES}}^{\text{NH}2‐\text{ZnO}}}M$, whereby the IFP was achieved as 31.77. As reported in Table 6, with modification of nanoparticles, the IFP parameter shows growth, such that in the examined studies, this parameter has been below 2 for unmodified of particles, while increasing up to 423.6 for ${\text{PVDF}}^{\text{CMC}‐\text{ZnO}}M$ upon modification of nanoparticles [150].

#### 3.3.5 Efficiency of presence of nanostructures in improving the membrane performance in separating pharmaceutical pollutants from the wastewater

Elevation of the level of pharmaceutical compounds in water environments has become a major concern in recent years. Although drugs including antibiotics are usually detected at low concentrations, they may have harmful physiological consequences for humans and other living creatures. Thus, it is essential to recycle antibiotics and pharmaceutical compounds from the wastewater of industrial factories [181]. The conventional methods of biological treatment of wastewater are not efficient enough in removal of antibiotics. Membrane processes have advantages such as higher efficiency, reducing operational costs, decreasing energy consumption, and being environmentally friendly.

**Fig. 15:**
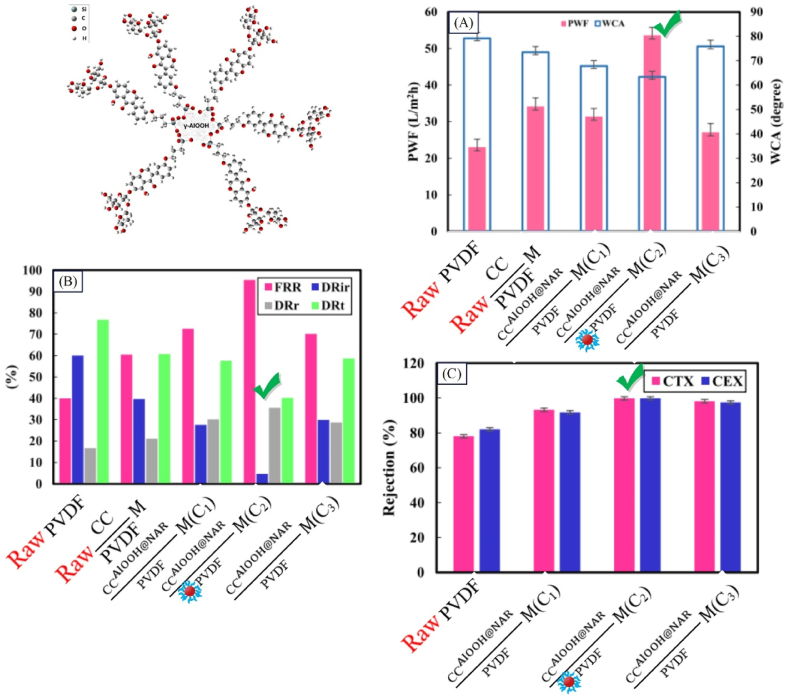
Performance results of a surface modified nanocomposite membrane sample with functionalized nanoparticles ($\frac{{\text{CC}}^{\mathrm{AlOOH}@\mathrm{NAR}}}{\text{PVDF}}M$) in the separation of pharmaceutical pollutants: A:water contact angle and pure water flux; B: fouling parameters; and C: rejection of the single solute (CTX or CEX) (adapted from Ref. [160]).

Extensive studies have been performed on addition of nanostructures to the membrane structure for separation of pharmaceutical pollutants. Various studies have been reviewed and further examined regarding the composition of the composite membrane including type of polymer, type of nanostructure, and concentration of nanostructure. Synthesized membranes have been used for removal of pharmaceutical pollutants. The results indicated that presence of nanostructure influences the flux and extent of removal. Moradi et al. [160] synthesized $\frac{{\text{CC}}^{\text{AlOOH}@\text{NAR}}}{\text{PVDF}}M$ for drug separation from wastewater. According to Fig. 15 (A), $\frac{CC}{\text{PVDF}}M$ has a higher water flux than PVDF membrane due to greater porosity and hydrophilicity. Due to the reduction of roughness and contact angle because of the presence of γ-AlOOH@Nar functionalized nanoparticles, the highest flux (according to Fig. 15 (A)) and the highest FRR (about 95.4 % according to Fig. 15 (B)) were related to $\frac{{\text{CC}}^{\text{AlOOH}@\text{NAR}}}{\text{PVDF}}M$. According to Fig. 15 (C), the $\frac{{\text{CC}}^{\text{AlOOH}@\text{NAR}}}{\text{PVDF}}M$ membranes show not only the highest flux but also the greatest rejection for both tested drug compounds (CTX and CEX).

Paseta et al. [151] employed $\frac{{\text{PA}}^{\text{ZIF}‐93\;}}{\text{PI}}M$ for separating diclofenac. The results indicated that the resulting membrane could separate 99 % of the drug from wastewater, and the flux of the synthesized membrane was found 242 LMH, indicating 255.8 % increase compared to the raw membrane. This increment in flux is related to the porosity of the MOF, higher hydrophilicity of the membrane, and higher surface roughness. Bojnourd and Pakizeh [152] utilized $\frac{\text{PVA}}{\text{PSf}}M$ for separating cephalexin. The results indicated that the resulting membranes could separate 99 % of the drug from wastewater, and the flux of membrane was achieved 14 LMH, revealing 58.8 % increase compared to the raw membrane. Considering the diversity of the examined results and for more precise investigation of studies, the IFP parameter was calculated according to Relation (7). The results with further details are outlined in Table 7.

**Table 7:** Inspection the effect of presence of nanoparticles on the performance of nanocomposite membranes in separating pharmaceutical pollutants from the wastewater.

Row	Nanocomposite membrane composition		Filtration condition			Filtration performance		IFP for drug separation	Reference
	Membrane name	NS/thin film conc.	Process	Pollutant Type	Drug Conc. (g/L)	Rejection (%)	Flux (LMH)	$\frac{\mathrm{Flux}\times \mathrm{Drug}\;\mathrm{Conc}}{\Delta P\times \mathrm{Consumed}\;\mathrm{NP}}$	
1	$\frac{{\text{PA}}^{\text{ZIF}-93\;}}{\text{PI}}M$^a^	20	NF	Diclofenac	0.001	99	242	**0.0012**	[151]
				Naproxen	0.001	99.3			
2	$\frac{{\text{PA}}^{\;\text{zeolite}}}{\text{PSf}}M$	0.75	NF	Carbamazepine	0.0002	85	58.916	**0.0015**	[39]
				Metoprolol	0.0002	82			
				Ranitidine	0.0002	84			
				Chloramphenicol	0.0002	84			
3	$\frac{\text{PVA}}{\text{PSf}}M$	1	NF	Cephalexin	0.050	99	14	**0.0875**	[152]
				Amoxicillin	0.050	97.7			
				IBU	0.050	74			
				PVP-I	0.050	93.8			
4	$\frac{\text{CNT}}{\text{PVC}}M$	_	UF	AAP	1	78	283.6	**-**	[4]
				CAF	0.5	90			
				TCS	0.5	90			
				CBD	0.5	94			
5	$\frac{\text{CNT}}{\text{PVDF}}M$	_	MF	TCS	0.001	93	_	**-**	[153]
				IBU	0.001	34			
				AAP	0.001	29			
				CBZ	0.001	70			
				ATAP	0.001	67			
				CAF	0.001	42			
				PTN	0.001	71			
				CBD	0.001	65			
6	$\frac{{\text{PVA}}^{\text{Clay}\;}}{\text{PSf}}M$	0.6	NF	Cephalexin	–	98.16	10.8	**-**	[154]
				Amoxicillin	–	95.4			
				IBU	–	83.37			
7	$\frac{{\text{PA}}^{\;\text{ZIF}-8\;}}{\text{PSf}}M$	1.21	NF	AAP	0.1	46	16	**0.331**	[3]
8	$\frac{\text{CNT}}{\text{PVDF}}M$	_	MF	AAP	0.001	77	_	**-**	[155]
				CAF	0.001	90			
				TCS	0.001	90			
				CBD	0.001	92			
9	$\frac{{\text{PA}}^{\frac{\text{PDA}}{\text{PEG}}@\text{ZIF}-8}}{\text{PES}}M$^b^	0.3	UF	VB12	0.01	98	46.8	**0.39**	[156]
				OTC	0.01	90			
10	$\frac{{\text{PDA}}^{\;\text{TiO}2}}{\text{polysulfonate}}M$	1.26	FO	TCS	0.0005	99	_	**-**	[157]
				MTP	0.0005	90			
				SMX	0.0005	99			
11	$\frac{{\text{rGO}}^{\;\text{CNT}\;}}{\text{PVDF}}M$	1	NF	AAP	1	62	0.4454	**4.454**	[158]
12	$\frac{{\text{PA}}^{\;\text{NH}2-\text{UiO}-66\;}}{\text{PI}}M$	0.75	NF	tetracycline	0.2	99	200	**5.33**	[159]
13	$\frac{{\text{PA}}^{\;\text{ZrO}2/\text{Fe}3O4}}{\text{PAN}}M$	0.17	NF	Cephalexin	0.05	78	105	**7.72**	[62]
14	$\frac{{\text{CC}}^{\text{AlOOH}@\text{NAR}}}{\text{PVDF}}M$	0.4	NF	Cephalexin	0.03	99.8	53.6	**10.05**	[160]
15	$\frac{{\text{PA}}^{\;\text{MIL}-53}}{\text{PSF}}M$	0.2	FO	doxycycline	1	98.5	11.27	**11.27**	[161]
16	$\frac{{AA}^{\text{AA}-\text{CuS}}}{\text{PSf}}M$	0.2	NF	Metronidazole	0.025	98.3	512.8	**16.025**	[33]

a PI: Polyamide.

b PEG: Poly (Ethylene Glycol).

**Fig. 16:**
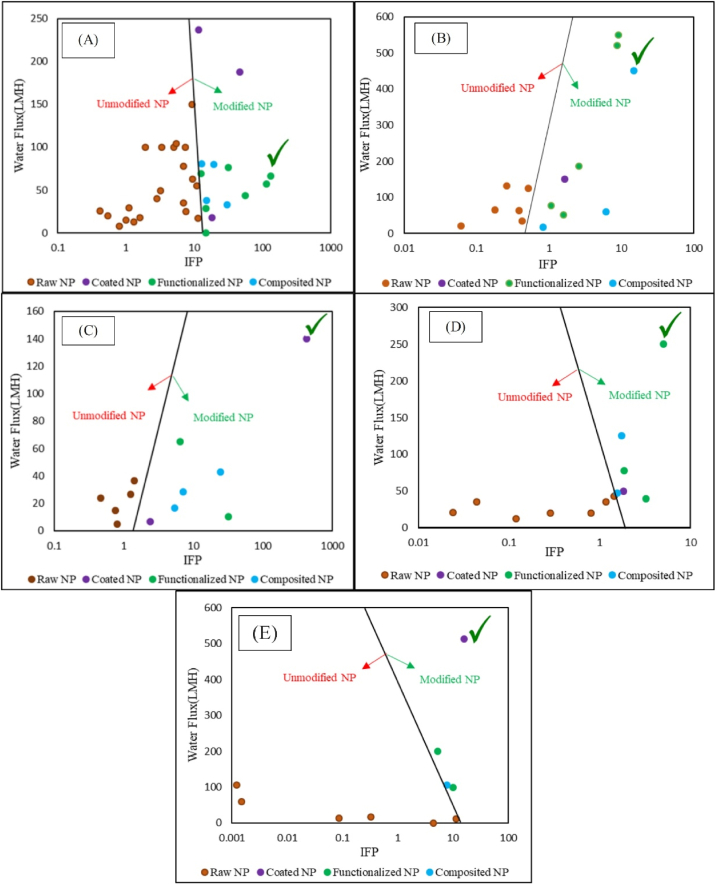
Investigating the effect of the presence and surface/structural modification of nanoparticles on the performance of nanocomposite membranes in separation of A: organic pollutants, B: filtration of textile wastewater, C: separation of salts, D: membrane bioreactors, and E: separation of pharmaceutical pollutants (More details about the presented data, have been provided in Table 3, Table 4, Table 5, Table 6, Table 7).

For separating cephalexin drug in the examined studies, $\frac{\text{PVA}}{\text{PSf}}M$ [152], $\frac{{\text{PA}}^{\;\text{ZrO}2/\text{Fe}3O4}}{\text{PAN}}M$ [62], and $\frac{{\text{CC}}^{\text{AlOOH}@\text{NAR}}}{\text{PVDF}}M$ [160] membranes were used, where IFP was reported 0.0875, 7.72, and 10.05 respectively, indicating the positive effect of presence of nanoparticles on the membrane performance. In another research, Guo et al. [159] utilized amine groups for functionalizing UiO-66 nanoparticles within the $\frac{{\text{PA}}^{\;\text{NH}2‐\text{UiO}‐66\;}}{\text{PI}}M$ structure where the IFP was calculated to be 5.33. As observed in Table 7, the IFP parameter for the membranes without modified nanoparticles has been lower than 5, while growing up to 16.025 in case of nanoparticle modification for $\frac{{\text{AA}}^{\;\text{AA}‐\text{CuS}}}{\text{PSf}}M$ [24]. Based on the IFP parameter calculated for the reviewed studies, the performance comparison of membrane containing modified and unmodified nanoparticle has been presented in Fig. 16. In the presented figure, the best nanoparticle modification approach in each category is presented already.

Based on Fig. 16, the use of modified nanoparticle compared to unmodified nanoparticle has improved the performance of the membrane and enhanced the value of IFP plus water flux. In the field of organic wastewater filtration (Fig. 16 (A)), the functionalization of nanoparticles resulted in the highest value of IFP (equal to 132.27) for the membrane. In the field of textile wastewater filtration (Fig. 16 (B)), the composite of nanoparticles resulted in the highest value of IFP (equal to 15) for the membrane containing modified nanoparticles. By examining the application of nanocomposite membranes in improving the filtration performance of brine wastewater (Fig. 16 (C)), it seems that the use of functionalized nanoparticles leads to a very significant increase in IFP and flux compared to unmodified nanoparticles. Application of nanocomposite membranes in improving the filtration performance of the membrane bioreactor (Fig. 16 (D)) would result in the highest IFP (equal to 5) and the highest flux for coated nanoparticles, showing a significant improvement compared to the membrane containing unmodified nanoparticles. Finally, nanoparticle coating has offered the best efficiency in improving membrane performance in pharmaceutical wastewater filtration (Fig. 16 (E)), so that the resulting IFP membrane has had a far higher flux than other types of membranes.

## 4 Challenges and future approaches/developments

Although recent review articles in the field of nanocomposite membranes have done valuable research in different fields such as the specific type of polymeric materials (such as polyamide [54]), or on a specific feature of the membrane (such as antibacterial [55], antimicrobial [56], or antifouling [7]), or specific application area (such as nanofiltration [51], desalination [52], or the removal of toxic substances [53]), no research has been done in the field of the site of loading (top layer or sublayer) and the structure of nanostructures (composite, coated, and functionalized). Since in this article, a unique parameter (IFP) was defined based on which the data of published articles in this field were reviewed and compared, so the results are comprehensive, generalizable, and replicable.

In this section, for practical usage by future researchers, the most widely used nanoparticle as well as its modification method and the best modified nanoparticle in five different fields of wastewater treatment are summarized. As a general conclusion, it can be stated that TiO_2_ has claimed the largest share (29 %) among nanoparticles for modification of nanocomposite membranes, followed by SiO_2_, Fe_3_O_4_, and ZnO with usage share of 24, 16, and 7 % respectively. Among the nanoparticle modification methods, functionalization with a usage share of 26 % of has attracted more attention compared to coating and compositing. Indeed, this high share of application is not surprising, since after functionalization of nanoparticles, the IFP has improved by 197 % on average, compared to usage of raw nanoparticles. Meanwhile, coating and compositing methods have been associated with 90 and 93 % improvement on average. In the modification method through functionalization, amine groups with 42 % were found to be used more than other functional groups. Indeed, application of this functional group caused the ${\text{CA}}^{\text{NH}2-\text{ND}}$ membrane to have IFP = 86. However, for ${\text{PES}}^{\text{MDA}‐\text{Fe}3O4}M$, ${\text{PES}}^{S‐\text{TiO}2}M$, ${\text{PES}}^{S\text{iO}2‐\text{TiO}2}M$, ${\text{PSf}}^{C\text{COOH}‐\text{ZrO}2}M$, and ${\text{PSf}}^{S\text{iO}4‐\text{ZrO}2}M$, the IFP parameter was calculated 62.5, 16, 7, 40, and 40.5 respectively.

The efficiency of presence of nanostructures in improving the membrane performance was examined for separation of organic pollutants from wastewater, filtration of textile wastewater, separation of pharmaceutical pollutants from wastewater, salt separation from wastewater, and improving membrane performance in membrane bioreactors. In the groups of separation of organic pollutants, filtration of textile wastewater, membrane bioreactors, separation of salts, and separation of pharmaceutical pollutants, IFP parameter has been lower than 11.5, 0.6, 14, 2, and 7.63 in studies in which unmodified nanoparticles have been used. However, the value of this parameter with modification of nanoparticles via functionalization methods has grown to 132.27 for the group of organic pollutants, to 15 for the groups of textile wastewater through compositing, to 5 for membrane bioreactors by coating, to 423.6 for brine wastewater through functionalization, and to 16.025 for pharmaceutical pollutants via coating method. Thus, investigations confirm presence of nanoparticles as well as modification of their structure on improving the performance of membrane filtration in different applications.

Regarding separation of organic wastewater, although TiO_2_ and SiO_2_ nanoparticles have been used more than other nanoparticles, the highest IFP was calculated for ${\text{PES}}^{{\text{NH}}_{2}‐\text{PDA}‐\text{Fe}3O4}M$ as 132.27. Thus, it is recommended that researchers interested in treatment of organic wastewater employ Fe_3_O_4_ nanoparticles as modified. In separation of textile dyes, although TiO_2_ has had greatest application, the largest IFP has been found for ZrO_2_. This parameter was calculated 6.1 and 15 for TiO_2_-containing of $\frac{{\text{PA}}^{\text{GO}\_\text{TiO}2}}{\text{PSf}}M$ and TiO_2_-containing ${\text{PAN}}^{\text{Fe}3O4/\text{ZrO}2}M$, respectively. Thus, researchers interested in removal of textile wastewater are proposed to use ZrO_2_ nanoparticles composited with iron oxide within the membrane matrix. In membrane bioreactors, Fe_3_O_4_ nanoparticles revealed the greatest application and highest IFP. This parameter was calculated 5 for the ${\text{PVDF}}^{\text{OCMCS}/\text{Fe}3O4}M$. Thus, usage of Fe_3_O_4_ seems to be better than other nanoparticles in membrane bioreactors. Also, in separation of salts, ZnO indicated the highest application and IFP value. The value of this parameter for the ${\text{PVDF}}^{\text{CMC}‐\text{ZnO}}M$ was calculated 423.6. Thus, researchers dealing with separation of salts are suggested to utilize this nanoparticle. When separating pharmaceutical pollutants, ZIFs indicated the greatest application. Nevertheless, the highest IFP was related to $\frac{{\text{AA}}^{\text{AA}‐\;\text{CuS}}}{\text{PSf}}M$, found as 16.025. Thus, CuS nanoparticles as modified in the top layer of membrane seem to offer the best performance.

## 5 Conclusion

Examination of 118 studies performed on application of nanocomposite membranes in the filtration of different types of wastewater, while emphasizing the effectiveness of nanoparticles on the membrane performance, revealed that the site of loading of nanoparticles and structural modification of nanoparticles (coating, compositing, and functionalization) determine the magnitude of effectiveness of nanoparticles. The investigations revealed that loading of nanoparticles in one layer, for $\frac{{\text{PA}}^{\text{Zeolite}}}{\text{PES}}M$ and $\frac{\text{PA}}{{\text{PSf}}^{\text{Zeolite}}}M$ was associated with −36 and −3% changes in the parameter of rejection enhancement. On the other hand, $\frac{{\text{PA}}^{\text{Fe}3O4/\text{ZnO}}}{{\text{PAN}}^{\text{ZnO}}}M$ and $\frac{{\text{PA}}^{\text{Zeolite}}}{{\text{PSf}}^{\text{Zeolite}}}M$ (containing nanoparticles in both membrane layers) showed 44.6 % and 8 % rejection enhancement. Investigation of different types of modifications applied onto the structure of nanoparticles also revealed that modification of Fe_3_O_4_ nanoparticles with MDA functional group led to IPF = 62.5, while this value for compositing this nanoparticle with ZrO_2_ as well as coated with PANI was found 42.8 and 45.45, respectively. Overall, a general comparison indicated that although incorporation of TiO_2_ nanostructure in the ${\text{PES}}^{\text{TiO}2}M$ matrix caused 9.52 % enhancement in IFP parameter compared to the raw membrane, which is an acceptable value, modification of the nanostructure with Ag and its incorporation in the $\frac{\text{PA}\;}{{\text{PSf}}^{\text{TiO}2/\text{Ag}}}M$ sublayer was associated with 24.63 % improvement compared to the raw membrane. Thus, in addition to the selection of the suitable type of nanostructures for each application, its loading site, as well as the surface modification performed on it also heavily determine the extent of effectiveness of presence of nanoparticles.

Examination of application of nanocomposite membranes in enhancing the organic wastewater filtration performance confirmed that the best IFP was achieved through incorporating Fe_3_O_4_ nanostructure modified with amine groups and PDA in the ${\text{PES}}^{{\text{NH}}_{2}‐\text{PDA}‐\text{Fe}3O4}M$ structure. Thus, regarding filtration of organic wastewater, functionalization of nanoparticles seems to improve the filtration performance. Investigation of the application of nanocomposite membranes in enhancing the textile wastewater filtration performance confirmed that the best IFP was obtained through incorporating Fe_3_O_4_ nanostructure composited with ZrO_2_ within the ${\text{PAN}}^{\text{Fe}3O4/\text{ZrO}2}M$ structure. Examination of application of nanocomposite membranes in boosting the filtration performance of membrane bioreactor confirmed that the best IFP was achieved through incorporating Fe_3_O_4_ nanostructure modified with OCMCS (${\text{PVDF}}^{\text{OCMCS}/\text{Fe}3O4}M$). Thus, it seems that coating iron oxide nanoparticles is the best solution for enhancing the membrane performance in membrane bioreactors. Investigation of use of nanocomposite membranes in augmenting the brine wastewater filtration performance confirmed that the best IFP was obtained by incorporating ZnO nanostructure modified with CMC within the ${\text{PVDF}}^{\text{CMC}/\text{ZnO}}M$ structure. Thus, use of coated ZnO nanostructure seems to function better in the filtration of brine wastewater. Exploration of the use of nanocomposite membranes in boosting the pharmaceutical wastewater filtration performance confirmed that the best IFP was achieved through incorporating CuS nanostructure modified with AA in the top layer ($\frac{{\text{AA}}^{\text{AA}‐\;\text{CuS}}}{\text{PSf}}M$). Therefore, coating nanoparticles and their incorporation in the top layer seem to offer the best efficiency in boosting the membrane performance in pharmaceutical wastewater filtration.

## Data availability statement

Data will be made available on request.

## CRediT authorship contribution statement

**Fateme Tahmasebi Sefiddashti:** Writing – original draft, Investigation, Data curation, Conceptualization. **Maryam Homayoonfal:** Writing – review & editing, Supervision, Project administration, Methodology, Conceptualization.

## Declaration of competing interest

The authors declare that they have no known competing financial interests or personal relationships that could have appeared to influence the work reported in this paper.
