# Supplementary material for: Nanostructure-manipulated filtration performance in nanocomposite membranes: A comprehensive investigation for water and wastewater treatment
Source: Heliyon. 2024 Aug 26;10(17):e36874. doi: 10.1016/j.heliyon.2024.e36874 (PMC11419920; doi:10.1016/j.heliyon.2024.e36874)
Supplement: Multimedia component 1 [file mmc1.docx]

**Nanostructure-** **manipulated Filtration Performance in Nanocomposite Membranes: A Comprehensive Investigation for Water and Wastewater Treatment**

**Fateme Tahmasebi Sefiddashti, Maryam Homayoonfal ***

**Department of Chemical Engineering, College of Engineering, University of Isfahan, P.O. Box 81746-73441, Isfahan, Iran**

**^*^** Corresponding author email address: [m.homayoonfal@eng.ui.ac.ir](mailto:m.homayoonfal@eng.ui.ac.ir)

**Table S1 Different types of nanostructures used in the membrane structure based on studies published in during the years 2010-2023**

| **NP** | **Membrane** | **Ref** | **Membrane** | **Ref** | **Membrane** | **Ref** |
| --- | --- | --- | --- | --- | --- | --- |
| **TiO_2_** | $\frac{\mathrm{PVA}^{\mathrm{TiO}2}}{\mathrm{PVDF}}M$ | [1] | $\mathrm{PPESK}^{TiO2}M$ | [2] | $\mathrm{PVC}^{TiO2/GO}M$ | [3] |
|  | $\frac{\mathrm{PA}^{\mathrm{NH}2-TiO2}}{\mathrm{PES}}M$ | [4] | $\mathrm{PES}^{TiO2}M$ | [5] | $\mathrm{PC}^{PDA-TiO2}M$ | [6] |
|  | $\mathrm{CA}^{TiO2/Al2O3}M$ | [7] | ${PVDF/SPES}^{TiO2}M$ | [8] | $\mathrm{PVDF}^{TiO2/CNT}M$ | [9] |
|  | ${PVDF/PMMA}^{TiO2}M$ | [10] | $\mathrm{PES}^{TiO2/\mathrm{MWCNT}}M$ | [11] | $\mathrm{PVDF}^{PPy-TiO2}M$ | [12] |
|  | $\mathrm{PVDF}^{\mathrm{TiO}2}M$ | [13] | $\frac{\text{PVA}^{\text{TiO2}}}{\text{PES}}M$ | [14] | $\mathrm{PVC}^{TiO2/ZnO}M$ | [15] |
|  | $\mathrm{PES}^{\mathrm{TiO}2}M$ | [16] | $\mathrm{PES}^{TiO2}M$ | [17] | $\frac{\mathrm{PA}^{\text{TiO2}}}{\mathrm{PES}}M$ | [18] |
|  | $\mathrm{PVDF}^{\mathrm{TiO}2/ZrO2}M$ | [19] | $\mathrm{PVDF}^{TiO2}M$ | [20] | $\frac{\mathrm{PA}^{APTMS-\text{TiO2}}}{\mathrm{PSF}}M$ | [21] |
|  | $\mathrm{PVDF}^{\mathrm{TiO}2}M$ | [22] | $\frac{\text{PVA}^{\text{TiO2}}}{\text{PS}}M$ | [23] | $\frac{TiO2}{\mathrm{PSF}}M$ | [24] |
|  | $\mathrm{CA}^{\mathrm{TiO}2}M$ | [25] | $\mathrm{PVDF}^{TiO2}M$ | [26] | ${polyurethane/CA}^{F-TiO2}M$ | [27] |
|  | $\mathrm{EPVC}^{\mathrm{TiO}2}M$ | [28] | $\frac{\text{PVA}^{\text{TiO2}}}{\mathrm{Polyester}}M$ | [29] | $\frac{\mathrm{PA}}{\mathrm{PES}^{PDA-TiO2}}M$ | [30] |
|  | $\mathrm{CA}^{\mathrm{TiO}2}M$ | [31] | $\mathrm{PPSU}^{TiO2}M$ | [32] | $\frac{\mathrm{PA}^{\text{TiO2/CD}}}{\mathrm{PSF}}M$ | [33] |
|  | $\mathrm{PES}^{\mathrm{APTES}-TiO2}M$ | [34] | $\mathrm{PES}^{PANi-TiO2}M$ | [35] | $\frac{\mathrm{PVP}^{\text{TiO2}}}{\mathrm{CA}}M$ | [36] |
|  | $\mathrm{PSf}^{\mathrm{TiO}2}M$ | [37] | $\frac{\text{PA}^{\text{TiO2}}}{\mathrm{PAN}}M$ | [38] | $\mathrm{PAN}^{TiO2}M$ | [39] |
|  | $\mathrm{PES}^{HMDI-TiO2}M$ | [40] | $\mathrm{PP}^{TiO2}M$ | [41] | $\mathrm{PVDF}^{TiO2}M$ | [42] |
|  | $\mathrm{PVDF}^{\mathrm{TiO}2}M$ | [43] | $\mathrm{PVDF}^{TiO2/HNT}M$ | [44] | $\mathrm{PVDF}^{TiO2/BiVO4/CNT}M$ | [45] |
|  | $\frac{\mathrm{PA}}{\mathrm{PSf}^{\mathrm{TiO}2}}M$ | [46] | $\mathrm{PC}^{TiO2}M$ | [47] | $\mathrm{PA}^{TiO2/GO}M$ | [48] |
|  | ${PVDF/HFP}^{\mathrm{TiO}2}M$ | [49] | $\frac{\text{GO}^{\text{TiO2}}}{\mathrm{CA}}M$ | [50] | $\frac{{PVA/GO}^{\text{TiO2}}}{\mathrm{PVDF}}M$ | [51] |
|  | $\mathrm{PSF}^{TiO2}M$ | [52] | $\mathrm{PES}^{TiO2/Ni}M$ | [53] | $\mathrm{PVDF}^{PVP-TiO2}M$ | [54] |
|  | $\frac{\mathrm{PDA}^{\mathrm{TiO}2}}{\mathrm{polysulfonate}}M$ | [55] | $\frac{\mathrm{Polyester}^{\text{TA-TiO2}}}{\mathrm{PES}}M$ | [56] | $\mathrm{PES}^{TiO2}M$ | [57] |
|  | $\mathrm{PES}^{S-TiO2}M$ | [58] | $\mathrm{PES}^{CPTES-TiO2}M$ | [59] | $\frac{\mathrm{PA}^{\text{Pal-TiO2}}}{\mathrm{PSf}}M$ | [60] |
|  | $\mathrm{PES}^{SiO2-TiO2}M$ | [61] | $\mathrm{PVC}^{TiO2/HNT}M$ | [62] | $\mathrm{PES}^{TiO2}M$ | [63] |
|  | $\mathrm{PVA}^{PANI-TiO2}M$ | [64] | $\mathrm{PEI}^{TiO2}M$ | [65] | $\mathrm{PSf}^{TiO2}M$ | [66] |
|  | $\mathrm{PVDF}^{PANI-TiO2}M$ | [67] | $\mathrm{PES}^{TiO2}M$ | [68] | ${CA/PVA}^{TiO2}M$ | [69] |
|  | ${PVDF/SPES}^{TiO2}M$ | [70] | $\frac{\mathrm{Pebax}^{\text{TiO2}}}{PFS/PES}M$ | [71] | $\mathrm{PVDF}^{TiO2/GO}M$ | [72] |
|  | ${PES/CA/PVP}^{TiO2}M$ | [73] | $\mathrm{PES}^{S-TiO2}M$ | [74] |  |  |
| **SiO_2_** | ${PVDF/PFSA}^{SiO2}M$ | [75] | $\frac{\mathrm{PA}^{\text{SiO2}}}{\mathrm{PSF}}M$ | [76] | ${PPSU/PES}^{SiO2}M$ | [77] |
|  | ${CA/AEDOT}^{SiO2}M$ | [78] | $\mathrm{HDPE}^{\mathrm{SiO}2}M$ | [79] | $\frac{{PDA/PEI}^{\text{NH2-SiO2}}}{\mathrm{PSF}}M$ | [80] |
|  | $\mathrm{PVA}^{\mathrm{SiO}2}M$ | [81] | $\mathrm{PVDF}^{SiO2/GO}M$ | [82] | $\mathrm{PVA}^{PDA-SiO2}M$ | [83] |
|  | $\mathrm{PVDF}^{\mathrm{SiO}2}M$ | [84] | $\mathrm{HDPE}^{F-SiO2}M$ | [85] | ${PVDF/PMMA/CA}^{SiO2}M$ | [86] |
|  | $\mathrm{PVC}^{\mathrm{SiO}2}M$ | [87] | $\mathrm{CA}^{SiO2}M$ | [88] | $\mathrm{PVA}^{SiO2}M$ | [89] |
|  | $\frac{\mathrm{PA}^{\text{NH2-SiO2}}}{PES/Si3N4}M$ | [90] | $\mathrm{PVDF}^{SiO2}M$ | [91] | $\mathrm{CTA}^{L\mathrm{udox}-SiO2}M$ | [92] |
|  | $\frac{\mathrm{PDA}^{\text{SiO2/TiO2}}}{\mathrm{PVDF}}M$ | [93] | $\mathrm{PSf}^{SiO2}M$ | [94] | $\mathrm{PVDF}^{SiO2}M$ | [95] |
|  | $\frac{\mathrm{PA}^{\mathrm{SiO}2}}{\mathrm{PSf}}M$ | [96] | $\mathrm{PAN}^{PDA-SiO2}M$ | [97] | ${PS/PVP}^{SiO2}M$ | [98] |
|  | $\frac{\mathrm{PA}^{\text{NH2-SiO2}}}{\mathrm{PES}}M$ | [99] | $\frac{\mathrm{PA}^{\text{OA-SiO2}}}{\mathrm{PSF}}M$ | [100] | $\mathrm{PES}^{PEI-SiO2}M$ | [101] |
|  | $\frac{\mathrm{PA}^{GO-SiO2}}{\mathrm{PSF}}M$ | [102] | $\frac{\mathrm{PA}^{\text{SiO2}}}{\mathrm{PS}}M$ | [103] | $\mathrm{PAN}^{SiO2/GO}M$ | [104] |
|  | $\mathrm{PSf}^{\mathrm{NH}2-\mathrm{SiO}2}M$ | [105] | $\frac{\mathrm{PA}^{\text{OA-SiO2}}}{\mathrm{PSF}}M$ | [106] | $\frac{\mathrm{PA}^{m\text{-SiO2}}}{\mathrm{PES}}M$ | [107] |
|  | $\frac{\mathrm{PA}^{\mathrm{Fluorinatedsilica}}}{\mathrm{PES}}M$ | [108] | $\mathrm{PSF}^{S-TiO2/SiO2}M$ | [109] | $\mathrm{PES}^{NH2-SiO2/COOH-\mathrm{MWCNT}}M$ | [110] |
|  | $\mathrm{PSf}^{PEI-SiO2}M$ | [111] | $\mathrm{PES}^{PSS-SiO2}M$ | [112] | $\mathrm{PSf}^{SiO2/TiO2}M$ | [113] |
|  | $\frac{\mathrm{PA}^{\text{SiO2}}}{\mathrm{PAN}}M$ | [114] | $\mathrm{PES}^{SiO2}M$ | [115] | ${PVDF-g-PMMA}^{SiO2/GO}M$ | [116] |
|  | ${PSf/PF127}^{SiO2}M$ | [117] | $\mathrm{PSF}^{SiO2/GO}M$ | [118] | $\mathrm{CA}^{SiO2/GO}M$ | [119] |
|  | $\frac{\mathrm{PEI}^{\text{SiO2}}}{\mathrm{PSF}}M$ | [120] | $\frac{\mathrm{PA}}{\mathrm{PEI}^{NH2-SiO2}}M$ | [121] | $\mathrm{PES}^{PEI-SiO2}M$ | [122] |
|  | $\frac{\mathrm{PA}^{\text{SiO2}}}{\mathrm{PSF}}M$ | [123] | $\mathrm{PES}^{N-Halamine-SiO2}M$ | [124] | $\mathrm{PVDF}^{PEI-SiO2}M$ | [125] |
|  | $\frac{\mathrm{PA}^{\mathrm{NH}2-SiO2/SiC}}{Al2O3}M$ | [126] | $\mathrm{PSF}^{SiO2}M$ | [127] | ${PEI/PVP}^{\mathrm{SiO}2}M$ | [128] |
|  | $\frac{\mathrm{PDA}^{\text{NH2-SiO2}}}{\mathrm{PES}}M$ | [129] | $\mathrm{PSf}^{SiO2}M$ | [130] | $\mathrm{PVDF}^{\mathrm{SiO}2/Cu}M$ | [131] |
|  | $\mathrm{PVDF}^{APTES-SiO2/Ag}M$ | [132] | $\mathrm{PES}^{SiO2/GO}M$ | [133] | $\mathrm{Psf}^{Ag/Cu/SiO2}M$ | [134] |
|  | $\frac{\mathrm{PDA}^{\text{SiO2}}}{\mathrm{PVDF}}M$ | [135] | $\mathrm{GO}^{SiO2}M$ | [136] |  |  |
| **Fe_3_O_4_** | $\frac{\mathrm{PA}^{\mathrm{Fe}3O4/ZrO2}}{\mathrm{PAN}^{ZrO2}}$M | [137] | $\mathrm{PES}^{Fe3o4/GO}M$ | [138] | $\mathrm{PES}^{Fe3O4/MoS2}M$ | [139] |
|  | $\mathrm{PVDF}^{Fe3O4/XG}M$ | [140] | $\mathrm{PVDF}^{\mathrm{Fe}3O4/HNT}M$ | [141] | $\mathrm{PVDF}^{TiO2/Fe3O4}M$ | [142] |
|  | $\mathrm{PES}^{MDA-Fe3O4}M$ | [143] | $\mathrm{PVC}^{\mathrm{Fe}3O4/Ag}M$ | [144] | $\mathrm{PSF}^{Fe3O4/GO}M$ | [145] |
|  | $\mathrm{PES}^{PVP-Fe3o4}M$ | [146] | $\mathrm{CA}^{Fe3O4/MXene}M$ | [147] | $\mathrm{PVDF}^{PGS-Fe3O4}M$ | [148] |
|  | $\mathrm{PAN}^{\mathrm{Fe}3O4/ZrO2}M$ | [149] | $\mathrm{PSf}^{talc-Fe3O4}M$ | [150] | $\mathrm{PES}^{8-HQFe3O4}M$ | [151] |
|  | $\mathrm{PVC}^{Fe3O4/O-MWCNT}M$ | [152] | $\mathrm{PVDF}^{Fe3O4}M$ | [153] | $\mathrm{PVDF}^{TiO2/Fe3O4}M$ | [154] |
|  | $\mathrm{PES}^{\mathrm{COOOH}-\mathrm{Fe}3O4/SiO2}M$ | [155] | $\mathrm{PES}^{NH2-Fe3O4}M$ | [156] | $\frac{\mathrm{GO}^{NH2-Fe3O4}}{\mathrm{PVDF}}M$ | [157] |
|  | $\mathrm{PES}^{NH2-PDA-Fe3O4}M$ | [158] | $\mathrm{PES}^{Cu/Fe3O4}M$ | [159] | $\mathrm{PES}^{APTES-Fe3O4}M$ | [160] |
|  | $\mathrm{PAN}^{\mathrm{Fe}3O4/ZrO2}M$ | [161] | $\mathrm{PVC}^{GA-Fe3O4}M$ | [162] | $\mathrm{PVDF}^{g-C3N4/Fe3O4}M$ | [163] |
|  | $\mathrm{PVDF}^{OCMCS/Fe3O4}M$ | [164] | $\mathrm{PES}^{Fe3O4}M$ | [165] | $\frac{{chitosan/PVA}^{NH2-Fe3O4}}{\mathrm{PES}}M$ | [166] |
|  | $\mathrm{PVDF}^{OCMCS/Fe3O4}M$ | [167] | $\mathrm{PSF}^{Fe3O4/GO}M$ | [168] | ${PEES/PVP}^{Fe3O4}M$ | [169] |
|  | $\mathrm{PES}^{\mathrm{NH}2-Fe3O4/SiO2}M$ | [170] | ${PES/PVP}^{NH2-Fe3O4}M$ | [171] | $\mathrm{PES}^{PAA-Fe3O4}M$ | [172] |
|  | $\mathrm{PES}^{PANI-Fe3o4}M$ | [173] | $\mathrm{PES}^{Fe3O4/HNC}M$ | [174] | $\mathrm{PSF}^{Fe3O4/GO}M$ | [175] |
|  | $\mathrm{PVDF}^{\mathrm{Fe}3O4}M$ | [176] |  |  |  |  |
| **ZnO** | $\frac{\mathrm{PA}^{\mathrm{Fe}3O4/ZnO}}{\mathrm{PES}^{\mathrm{Fe}3O4/ZnO}}M$ | [177] | $\mathrm{PES}^{silane-ZnO}M$ | [178] | $\mathrm{PES}^{NH2-ZnO}M$ | [179] |
|  | $\mathrm{PVDF}^{\mathrm{ZnO}}M$ | [180] | $\mathrm{PES}^{\mathrm{ZnO}}M$ | [181] | $\mathrm{PES}^{ZnO/GO}M$ | [182] |
|  | $\mathrm{PES}^{Cu/ZnO}M$ | [183] | $\mathrm{PES}^{\mathrm{ZnO}}M$ | [184] | $\mathrm{PU}^{ZnO/CdS}M$ | [185] |
|  | $\mathrm{PES}^{MWCNT/ZnO}M$ | [186] | $\mathrm{PVDF}^{Ag/ZnO}M$ | [187] | $\frac{\mathrm{PVA}^{\text{NH2-ZnO}}}{\mathrm{PC}}M$ | [188] |
|  | $\frac{\mathrm{PDA}^{\mathrm{TiO}2/ZnO}}{\mathrm{PA}}M$ | [189] | $\mathrm{PSF}^{ZnO/MnO2/SiO2}M$ | [190] | $\mathrm{CA}^{\mathrm{ZnO}}M$ | [191] |
|  | $\frac{\mathrm{PA}}{\mathrm{PES}^{NH2-ZnO}}$ | [192] | $\mathrm{BC}^{TiO2/ZnO}M$ | [193] | PAN^SA-ZnO^M | [194] |
| **Ag** | $\frac{\mathrm{PA}^{\mathrm{Ag}}}{\mathrm{PSf}}M$ | [195] | $\mathrm{PVDF}^{Ag/SiO2}M$ | [196] | $\frac{\mathrm{PA}^{\mathrm{Ag}}}{\mathrm{PSU}^{\mathrm{MWNT}}}$ | [197] |
|  | $\frac{\text{PA}^{\text{rGO/TiO2/Ag}}}{\text{PES/Si3N4}}M$ | [198] | $\mathrm{PES}^{PVP-DDT-Ag}$ | [199] | $\frac{\mathrm{PDA}^{\text{Ag/MoS2/TiO2}}}{\mathrm{PSF}}M$ | [200] |
|  | $\frac{\mathrm{PA}}{\mathrm{PSf}^{TiO2/Ag}}M$ | [201] | $\mathrm{PES}^{FBN/GO/Ag}M$ | [202] | $\mathrm{PVDF}^{TiO2/Ag}M$ | [203] |
|  | $\mathrm{PES}^{SiO2/Ag}M$ | [204] | $\frac{\text{PA}^{\text{NH2-rGO/TiO2/Ag}}}{\text{PAN/Si3N4}}M$ | [205] | $\frac{\mathrm{PA}^{\text{TiO2/Ag}}}{\mathrm{PES}}M$ | [206] |
|  | $\frac{\mathrm{PDA}^{PEI-TiO2\_Ag}}{\mathrm{PAN}}M$ | [207] |  |  |  |  |
| **Other** | $\mathrm{PSf}^{\mathrm{COOH}-ZrO2}M$ | [208] | $\mathrm{PES}^{\mathrm{ZrO}2}M$ | [209] | $\frac{\mathrm{PDA}^{\text{NH2-MWCNT}}}{\mathrm{PES}}M$ | [210] |
|  | $\frac{\mathrm{PA}}{\mathrm{PSf}^{\mathrm{Zeolite}}}$M | [211] | $\mathrm{PAN}^{\mathrm{COOH}-ZrO2}M$ | [212] | $\frac{\mathrm{PA}^{\mathrm{Zeolite}}}{\mathrm{PES}}M$ | [213] |
|  | $\frac{\mathrm{PA}^{\text{GO}}}{\mathrm{PSF}}M$ | [214] | $\mathrm{PSF}^{COOH-CNT}M$ | [215] | $\frac{\mathrm{PA}^{\mathrm{NH}2-UiO-66}}{\mathrm{PI}}M$ | [216] |
|  | $\mathrm{CA}^{NH2-SiC}M$ | [217] | $\frac{\mathrm{PVA}^{\mathrm{Clay}}}{\mathrm{PSf}}M$ | [218] | $\mathrm{PAN}^{G\mathrm{oethite}}M$ | [219] |
|  | $\frac{\mathrm{PA}^{\mathrm{Zeolite}}}{\mathrm{PSf}^{\mathrm{Zeolite}}}M$ | [220] | $\frac{\mathrm{PA}^{ZIF-8}}{\mathrm{PSf}}M$ | [221] | $\frac{\mathrm{PA}^{Fe-NH2-clay}}{\mathrm{PAN}}M$ | [222] |
|  | $\mathrm{PSF}^{Al2O3}M$ | [223] | $\frac{\mathrm{PA}^{MIL-53}}{\mathrm{PSF}}M$ | [224] | $\frac{\mathrm{polypiperazineamide}^{NH2-ND}}{\mathrm{PA}}M$ | [225] |
|  | $\mathrm{PAN}^{Al2O3}M$ | [226] | $\frac{\mathrm{PA}^{\frac{\mathrm{PDA}}{\mathrm{PEG}}@ZIF-8}}{\mathrm{PES}}M$ | [227] | $\frac{\mathrm{PA}^{NH2-CQD}}{\mathrm{PES}}M$ | [228] |
|  | $\mathrm{PES}^{\mathrm{FeON}}M$ | [229] | $\mathrm{PES}^{\mathrm{PABFNP}}M$ | [230] | $\frac{\mathrm{PA}^{NH2-GO}}{\mathrm{PES}}M$ | [231] |
|  | $\mathrm{PES}^{CeO2}M$ | [232] | $\frac{\mathrm{PA}^{\mathrm{Zeolite}}}{\mathrm{PS}}M$ | [233] | $\frac{\mathrm{PA}^{COOH-COF}}{\mathrm{MCE}}M$ | [234] |
|  | $\mathrm{PES}^{F-carbon dots}M$ | [235] | $\mathrm{PES}^{\mathrm{NH}2-ZiF-8/GO}$ | [236] | $\frac{\mathrm{PA}^{COOH-MoS2}}{\mathrm{PES}}M$ | [237] |
|  | $\frac{{AA}^{AA-CuS}}{\mathrm{PSf}}M$ | [238] | $\mathrm{PVDF}^{\mathrm{APDEMS}-SiC}M$ | [239] | $\mathrm{CA}^{COOH-ND}M$ | [240] |
|  | $\frac{\mathrm{PA}^{Fe(0)}}{\mathrm{PSf}}M$ | [241] | $\mathrm{PVC}^{PDA-Al2O3}M$ | [242] | $\frac{\mathrm{PA}^{\mathrm{FeO}}}{\mathrm{PES}}M$ | [243] |
|  | $\mathrm{PSf}^{S-ND}M$ | [244] | $\mathrm{PVDF}^{COOH-MWCNT}M$ | [245] | $\mathrm{PVDF}^{COOH-FO}M$ | [246] |
|  | $\mathrm{CA}^{\mathrm{NH}2-ND}$ | [247] | $\mathrm{PSF}^{\mathrm{NH}2-NC}$ | [248] | $\mathrm{PVDF}^{polyhexanide-\mathrm{CuO}}M$ | [249] |
|  | $\mathrm{PES}^{PDA-ZnFe2O4}M$ | [250] | $\mathrm{GO}^{PAA-NH2-UiO-66}M$ | [251] | $\frac{\mathrm{PA}^{COOH-CN}}{\mathrm{PSF}}M$ | [252] |
|  | $\frac{\mathrm{PA}^{NH2-CQD}}{\mathrm{PSF}}M$ | [253] | $\frac{\mathrm{CNT}}{\mathrm{PVDF}}M$ | [254] | $\frac{\mathrm{PA}^{ZIF-93}}{\mathrm{PI}}M$ | [255] |
|  | $\mathrm{PVDF}^{Mg-Al}M$ | [256] |  |  |  |  |

**Table S2 different types of structural modifications done on nanostructures based on studies published in during the years 2010-2023**

| **Modified method** | **Membrane** | **Ref** | **Membrane** | **Ref** | **Membrane** | **Ref** |
| --- | --- | --- | --- | --- | --- | --- |
| **Unmodified NP** | $\frac{\mathrm{PA}^{\mathrm{Zeolite}}}{\mathrm{PES}}M$ | [213] | $\frac{\mathrm{PDA}^{\mathrm{TiO}2}}{\mathrm{polysulfonate}}M$ | [55] | $\frac{\mathrm{Pebax}^{\text{TiO2}}}{PFS/PES}M$ | [71] |
|  | $\frac{\mathrm{PVA}^{\mathrm{TiO}2}}{\mathrm{PVDF}}M$ | [1] | $\frac{\mathrm{PA}^{MIL-53}}{\mathrm{PSF}}M$ | [224] | $\mathrm{CA}^{SiO2}M$ | [88] |
|  | $\frac{\mathrm{PA}^{Fe(0)}}{\mathrm{PSf}}M$ | [241] | $\frac{\mathrm{PA}^{\text{GO}}}{\mathrm{PSF}}M$ | [214] | $\mathrm{PSf}^{SiO2}M$ | [94] |
|  | $\frac{\mathrm{PA}^{\mathrm{Ag}}}{\mathrm{PSf}}M$ | [195] | ${PVDF/HFP}^{\mathrm{TiO}2}M$ | [49] | $\frac{\mathrm{PA}^{\text{SiO2}}}{\mathrm{PS}}M$ | [103] |
|  | $\frac{\mathrm{PA}}{\mathrm{PSf}^{\mathrm{Zeolite}}}$M | [211] | $\mathrm{PSF}^{TiO2}M$ | [52] | $\mathrm{PES}^{SiO2}M$ | [115] |
|  | $\frac{\mathrm{PA}^{\mathrm{Zeolite}}}{\mathrm{PSf}^{\mathrm{Zeolite}}}M$ | [220] | ${PES/CA/PVP}^{TiO2}M$ | [73] | $\mathrm{PSF}^{SiO2/GO}M$ | [118] |
|  | $\mathrm{PVDF}^{\mathrm{TiO}2}M$ | [13] | $\frac{\mathrm{PA}^{\text{SiO2}}}{\mathrm{PAN}}M$ | [114] | $\mathrm{PSF}^{SiO2}M$ | [127] |
|  | ${PVDF/PFSA}^{SiO2}M$ | [75] | ${PSf/PF127}^{SiO2}M$ | [117] | $\mathrm{PSf}^{SiO2}M$ | [130] |
|  | $\mathrm{PES}^{\mathrm{TiO}2}M$ | [16] | $\frac{\mathrm{PA}^{\text{SiO2}}}{\mathrm{PSF}}M$ | [120] | ${PVDF/PMMA/CA}^{SiO2}M$ | [86] |
|  | $\mathrm{PVA}^{\mathrm{SiO}2}M$ | [81] | $\frac{\mathrm{PA}^{\text{SiO2}}}{\mathrm{PSF}}M$ | [123] | $\mathrm{PVA}^{SiO2}M$ | [89] |
|  | $\mathrm{PVDF}^{\mathrm{TiO}2}M$ | [22] | ${PVDF/PMMA}^{TiO2}M$ | [10] | $\mathrm{PVDF}^{SiO2}M$ | [95] |
|  | $\mathrm{CA}^{\mathrm{TiO}2}M$ | [25] | ${CA/AEDOT}^{SiO2}M$ | [78] | ${PS/PVP}^{SiO2}M$ | [98] |
|  | $\mathrm{EPVC}^{\mathrm{TiO}2}M$ | [28] | $\mathrm{PVDF}^{Fe3o4}M$ | [153] | ${PEI/PVP}^{\mathrm{SiO}2}M$ | [128] |
|  | $\mathrm{PVDF}^{Mg-Al}M$ | [256] | $\frac{\mathrm{PA}^{ZIF-8}}{\mathrm{PSf}}M$ | [221] | $\mathrm{PES}^{Fe3o4}M$ | [165] |
|  | $\mathrm{PVDF}^{\mathrm{SiO}2}M$ | [84] | ${PVDF/SPES}^{TiO2}M$ | [70] | $\mathrm{PSf}^{TiO2}M$ | [66] |
|  | $\mathrm{PVDF}^{\mathrm{ZnO}}M$ | [180] | $\frac{\mathrm{PA}^{\mathrm{Zeolite}}}{\mathrm{PS}}M$ | [233] | ${CA/PVA}^{TiO2}M$ | [69] |
|  | $\mathrm{CA}^{\mathrm{TiO}2}M$ | [31] | $\mathrm{PPESK}^{TiO2}M$ | [2] | $\frac{\mathrm{PA}^{\text{TiO2}}}{\mathrm{PES}}M$ | [18] |
|  | $\mathrm{PES}^{\mathrm{ZrO}2}M$ | [209] | $\mathrm{PES}^{TiO2}M$ | [5] | $\frac{TiO2}{\mathrm{PSF}}M$ | [24] |
|  | $\mathrm{PVC}^{\mathrm{SiO}2}M$ | [87] | ${PVDF/SPES}^{TiO2}M$ | [8] | $\frac{\mathrm{PVP}^{\text{TiO2}}}{\mathrm{CA}}M$ | [36] |
|  | $\mathrm{PES}^{\mathrm{FeON}}M$ | [229] | $\frac{\text{PVA}^{\text{TiO2}}}{\text{PES}}M$ | [14] | $\mathrm{PAN}^{TiO2}M$ | [39] |
|  | $\mathrm{PES}^{CeO2}M$ | [232] | $\mathrm{PES}^{TiO2}M$ | [17] | $\mathrm{PVDF}^{TiO2}M$ | [42] |
|  | $\mathrm{PSf}^{\mathrm{TiO}2}M$ | [37] | $\mathrm{PVDF}^{TiO2}M$ | [20] | $\frac{{PVA/GO}^{\text{TiO2}}}{\mathrm{PVDF}}M$ | [51] |
|  | $\mathrm{PVDF}^{\mathrm{TiO}2}M$ | [43] | $\frac{\text{PVA}^{\text{TiO2}}}{\text{PS}}M$ | [23] | $\mathrm{PES}^{TiO2}M$ | [57] |
|  | $\mathrm{PSF}^{Al2O3}M$ | [223] | $\mathrm{PVDF}^{TiO2}M$ | [26] | $\mathrm{PES}^{TiO2}M$ | [63] |
|  | $\mathrm{PAN}^{Al2O3}M$ | [226] | $\mathrm{PES}^{TiO2}M$ | [68] | ${PEES/PVP}^{Fe3O4}M$ | [169] |
|  | $\mathrm{HDPE}^{\mathrm{SiO}2}M$ | [79] | $\frac{\text{PVA}^{\text{TiO2}}}{\mathrm{Polyester}}M$ | [29] | $\mathrm{PVDF}^{SiO2}M$ | [91] |
|  | $\frac{\mathrm{PA}^{\mathrm{FeO}}}{\mathrm{PES}}M$ | [243] | $\frac{\mathrm{PA}^{\text{SiO2}}}{\mathrm{PSF}}M$ | [76] | ${PPSU/PES}^{SiO2}M$ | [77] |
|  | $\frac{\mathrm{PA}}{\mathrm{PSf}^{\mathrm{TiO}2}}M$ | [46] | $\frac{\mathrm{PVA}^{\mathrm{Clay}}}{\mathrm{PSf}}M$ | [218] | $\mathrm{PPSU}^{TiO2}M$ | [32] |
|  | $\mathrm{PVDF}^{\mathrm{Fe}3O4}M$ | [176] | $\mathrm{PES}^{TiO2}M$ | [184] | $\mathrm{PAN}^{G\mathrm{oethite}}M$ | [219] |
|  | $\mathrm{CA}^{\mathrm{ZnO}}M$ | [191] | $\frac{\text{PA}^{\text{TiO2}}}{\mathrm{PAN}}M$ | [38] | $\mathrm{PES}^{\mathrm{ZnO}}M$ | [181] |
|  | $\frac{\mathrm{PA}^{\mathrm{SiO}2}}{\mathrm{PSf}}M$ | [96] | $\mathrm{PP}^{TiO2}M$ | [41] | $\mathrm{PEI}^{TiO2}M$ | [65] |
|  | $\frac{\mathrm{PA}^{ZIF-93}}{\mathrm{PI}}M$ | [255] | $\mathrm{PC}^{TiO2}M$ | [47] | $\frac{\mathrm{PDA}^{\text{SiO2}}}{\mathrm{PVDF}}M$ | [135] |
|  | $\frac{\mathrm{CNT}}{\mathrm{PVDF}}M$ | [254] | $\frac{\text{GO}^{\text{TiO2}}}{\mathrm{CA}}M$ | [50] | $\mathrm{GO}^{SiO2}M$ | [136] |
| **Functionalization NP** | $\frac{\mathrm{PA}^{\mathrm{NH}2-TiO2}}{\mathrm{PES}}M$ | [4] | $\frac{\mathrm{PDA}^{\text{NH2-SiO2}}}{\mathrm{PES}}M$ | [129] | $\mathrm{PVDF}^{COOH-FO}M$ | [246] |
|  | $\frac{\mathrm{PA}^{\mathrm{Fluorinatedsilica}}}{\mathrm{PES}}M$ | [108] | $\mathrm{PES}^{NH2-Fe3o4}M$ | [156] | $\mathrm{PES}^{NH2-ZnO}M$ | [179] |
|  | $\mathrm{PES}^{MDA-Fe3O4}M$ | [143] | $\mathrm{PVC}^{Fe3O4/O-MWCNT}M$ | [152] | $\frac{\mathrm{PA}^{\text{NH2-SiO2}}}{PES/Si3N4}M$ | [90] |
|  | $\mathrm{PES}^{S-TiO2}M$ | [58] | $\mathrm{PVDF}^{OCMCS/Fe3O4}M$ | [167] | $\mathrm{PES}^{8-HQFe3O4}M$ | [151] |
|  | $\mathrm{CA}^{\mathrm{NH}2-ND}$ | [247] | $\mathrm{PSf}^{talc-Fe3o4}M$ | [150] | $\frac{\mathrm{GO}^{NH2-Fe3O4}}{\mathrm{PVDF}}M$ | [157] |
|  | $\mathrm{PES}^{SiO2-TiO2}M$ | [61] | $\mathrm{PES}^{CPTES-TiO2}M$ | [59] | $\mathrm{PES}^{APTES-Fe3O4}M$ | [160] |
|  | $\mathrm{PSf}^{\mathrm{COOH}-ZrO2}M$ | [208] | $\mathrm{PES}^{S-TiO2}M$ | [74] | $\frac{{chitosan/PVA}^{NH2-Fe3O4}}{\mathrm{PES}}M$ | [166] |
|  | $\mathrm{CA}^{NH2-SiC}M$ | [217] | $\mathrm{HDPE}^{F-SiO2}M$ | [85] | $\mathrm{PVC}^{GA-Fe3o4}M$ | [162] |
|  | $\mathrm{PES}^{\mathrm{APTES}-TiO2}M$ | [34] | $\frac{\mathrm{PA}^{\text{OA-SiO2}}}{\mathrm{PSF}}M$ | [100] | $\mathrm{PES}^{silane-ZnO}M$ | [178] |
|  | $\mathrm{PSf}^{\mathrm{NH}2-\mathrm{SiO}2}M$ | [105] | $\mathrm{PSF}^{S-TiO2/SiO2}M$ | [109] | $\mathrm{PES}^{\mathrm{PABFNP}}M$ | [230] |
|  | $\mathrm{PES}^{\mathrm{COOOH}-\mathrm{Fe}3O4/SiO2}M$ | [155] | $\frac{\mathrm{PA}}{\mathrm{PEI}^{NH2-SiO2}}M$ | [121] | $\frac{\mathrm{PA}^{Fe-NH2-clay}}{\mathrm{PAN}}M$ | [222] |
|  | $\mathrm{PSF}^{COOH-CNT}M$ | [215] | $\mathrm{PES}^{N-Halamine-SiO2}M$ | [124] | $\frac{\mathrm{polypiperazineamide}^{NH2-ND}}{\mathrm{PA}}M$ | [225] |
|  | ${PES/PVP}^{NH2-Fe3o4}M$ | [171] | $\frac{{PDA/PEI}^{\text{NH2-SiO2}}}{\mathrm{PSF}}M$ | [80] | $\frac{\mathrm{PA}^{NH2-CQD}}{\mathrm{PES}}M$ | [228] |
|  | $\mathrm{PAN}^{\mathrm{COOH}-ZrO2}M$ | [212] | $\frac{\mathrm{PA}^{m\text{-SiO2}}}{\mathrm{PES}}M$ | [107] | $\frac{\mathrm{PVA}^{\text{NH2-ZnO}}}{\mathrm{PC}}M$ | [188] |
|  | $\mathrm{PES}^{F-carbon dots}M$ | [235] | $\mathrm{PES}^{NH2-SiO2/COOH-\mathrm{MWCNT}}M$ | [110] | $\frac{\mathrm{PA}^{\text{NH2-SiO2}}}{\mathrm{PES}}M$ | [99] |
|  | $\mathrm{PES}^{HMDI-TiO2}M$ | [40] | $\mathrm{PVDF}^{\mathrm{APDEMS}-SiC}M$ | [239] | $\frac{\mathrm{PA}^{NH2-GO}}{\mathrm{PES}}M$ | [231] |
|  | $\mathrm{PSf}^{S-ND}M$ | [244] | $\frac{\mathrm{PA}^{APTMS-\text{TiO2}}}{\mathrm{PSF}}M$ | [21] | $\frac{\mathrm{PA}^{COOH-COF}}{\mathrm{MCE}}M$ | [234] |
|  | $\mathrm{PVDF}^{OCMCS/Fe3O4}M$ | [164] | ${polyurethane/CA}^{F-TiO2}M$ | [27] | $\frac{\mathrm{PA}^{COOH-MoS2}}{\mathrm{PES}}M$ | [237] |
|  | $\frac{\mathrm{PA}^{COOH-CN}}{\mathrm{PSF}}M$ | [252] | $\mathrm{PVDF}^{COOH-MWCNT}M$ | [245] | $\mathrm{CA}^{COOH-ND}M$ | [240] |
|  | $\frac{\mathrm{PA}}{\mathrm{PES}^{NH2-ZnO}}$ | [192] | $\mathrm{PSF}^{\mathrm{NH}2-NC}$ | [248] | $\frac{\text{PA}^{\text{NH2-rGO/TiO2/Ag}}}{\text{PAN/Si3N4}}M$ | [205] |
|  | $\frac{\mathrm{PA}^{\mathrm{NH}2-UiO-66}}{\mathrm{PI}}M$ | [216] | $\mathrm{PES}^{\mathrm{NH}2-ZiF-8/GO}$ | [236] | $\mathrm{PVDF}^{APTES-SiO2/Ag}M$ | [132] |
|  | $\frac{\mathrm{PDA}^{\text{NH2-MWCNT}}}{\mathrm{PES}}M$ | [210] | $\frac{\mathrm{PA}^{NH2-CQD}}{\mathrm{PSF}}M$ | [253] | $\frac{\mathrm{PA}^{\text{OA-SiO2}}}{\mathrm{PSF}}M$ | [106] |
|  | $\frac{\mathrm{PA}^{\mathrm{NH}2-SiO2/SiC}}{Al2O3}M$ | [126] |  |  |  |  |
| **Coating NP** | $\mathrm{PSf}^{PEI-SiO2}M$ | [111] | $\frac{\mathrm{PA}^{\text{Pal-TiO2}}}{\mathrm{PSf}}M$ | [60] | $\mathrm{PES}^{PEI-SiO2}M$ | [122] |
|  | $\frac{{AA}^{AA-CuS}}{\mathrm{PSf}}M$ | [238] | PAN^SA-ZnO^M | [194] | $\mathrm{PVDF}^{PEI-SiO2}M$ | [125] |
|  | $\frac{\mathrm{PA}^{\frac{\mathrm{PDA}}{\mathrm{PEG}}@ZIF-8}}{\mathrm{PES}}M$ | [227] | $\mathrm{PVA}^{PANI-TiO2}M$ | [64] | $\mathrm{PVC}^{PDA-Al2O3}M$ | [242] |
|  | $\mathrm{PES}^{PVP-Fe3o4}M$ | [146] | $\mathrm{PES}^{PANi-TiO2}M$ | [35] | $\mathrm{PC}^{PDA-TiO2}M$ | [6] |
|  | $\mathrm{PES}^{PVP-DDT-Ag}$ | [199] | $\mathrm{PVDF}^{PANI-TiO2}M$ | [67] | $\mathrm{PVDF}^{PPy-TiO2}M$ | [12] |
|  | $\frac{\mathrm{PDA}^{PEI-TiO2\_Ag}}{\mathrm{PAN}}M$ | [207] | $\frac{\mathrm{Polyester}^{\text{TA-TiO2}}}{\mathrm{PES}}M$ | [56] | $\frac{\mathrm{PA}}{\mathrm{PES}^{PDA-TiO2}}$ | [30] |
|  | $\mathrm{GO}^{PAA-NH2-UiO-66}M$ | [251] | $\mathrm{PAN}^{PDA-SiO2}M$ | [97] | $\mathrm{PVDF}^{PVP-TiO2}M$ | [54] |
|  | $\mathrm{PES}^{NH2-PDA-Fe3O4}M$ | [158] | $\mathrm{PES}^{PSS-SiO2}M$ | [112] | $\mathrm{PVDF}^{XG-Fe3O4}M$ | [140] |
|  | $\mathrm{PES}^{PDA-ZnFe2O4}M$ | [250] | $\mathrm{PVA}^{PDA-SiO2}M$ | [83] | $\mathrm{PVDF}^{PGS-Fe3O4}M$ | [148] |
|  | $\mathrm{PVDF}^{polyhexanide-\mathrm{CuO}}M$ | [249] | $\mathrm{CTA}^{L\mathrm{udox}-SiO2}M$ | [92] | $\mathrm{PES}^{PAA-Fe3O4}M$ | [172] |
|  | $\mathrm{PES}^{PANI-Fe3o4}M$ | [173] | $\mathrm{PES}^{PEI-SiO2}M$ | [101] |  |  |
| **Compositing NP** | $\frac{\mathrm{PA}^{\mathrm{Fe}3O4/ZrO2}}{\mathrm{PAN}^{ZrO2}}$M | [137] | $\frac{\mathrm{PA}^{\mathrm{Ag}}}{\mathrm{PSU}^{\mathrm{MWNT}}}$ | [197] | $\mathrm{PVDF}^{TiO2/Ag}M$ | [203] |
|  | $\frac{\mathrm{PA}^{\mathrm{Fe}3O4/ZnO}}{\mathrm{PES}^{\mathrm{Fe}3O4/ZnO}}M$ | [177] | $\mathrm{PVDF}^{TiO2/HNT}M$ | [44] | $\mathrm{PES}^{Fe3O4/MoS2}M$ | [139] |
|  | $\mathrm{PAN}^{\mathrm{Fe}3O4/ZrO2}M$ | [149] | $\mathrm{PES}^{TiO2/Ni}M$ | [53] | $\mathrm{PVDF}^{TiO2/Fe3O4}M$ | [142] |
|  | $\mathrm{CA}^{TiO2/Al2O3}M$ | [7] | $\mathrm{PVDF}^{SiO2/GO}M$ | [82] | $\mathrm{PSF}^{Fe3o4/GO}M$ | [168] |
|  | $\frac{\mathrm{PA}}{\mathrm{PSf}^{TiO2/Ag}}M$ | [201] | $\mathrm{PES}^{SiO2/GO}M$ | [133] | $\mathrm{PSF}^{Fe3O4/GO}M$ | [175] |
|  | $\mathrm{PES}^{SiO2/Ag}M$ | [204] | $\frac{\mathrm{PDA}^{\text{SiO2/TiO2}}}{\mathrm{PVDF}}M$ | [93] | $\mathrm{PES}^{Fe3O4/HNC}M$ | [174] |
|  | $\mathrm{PVDF}^{\mathrm{TiO}2/ZrO2}M$ | [19] | $\mathrm{PSf}^{SiO2/TiO2}M$ | [113] | $\mathrm{PSF}^{Fe3O4/GO}M$ | [145] |
|  | $\mathrm{PES}^{TiO2/\mathrm{MWCNT}}M$ | [11] | ${PVDF-g-PMMA}^{SiO2/GO}M$ | [116] | $\mathrm{PVDF}^{TiO2/Fe3O4}M$ | [154] |
|  | $\mathrm{PES}^{Cu/ZnO}M$ | [183] | $\mathrm{PSF}^{ZnO/MnO2/SiO2}M$ | [190] | $\mathrm{PVDF}^{g-C3N4/Fe3O4}M$ | [163] |
|  | $\mathrm{PES}^{MWCNT/ZnO}M$ | [186] | $\mathrm{PVDF}^{\mathrm{SiO}2/Cu}M$ | [131] | $\mathrm{PES}^{ZnO/GO}M$ | [182] |
|  | $\mathrm{PAN}^{\mathrm{Fe}3O4/ZrO2}M$ | [161] | $\mathrm{Psf}^{Ag/Cu/SiO2}M$ | [134] | $\mathrm{PU}^{ZnO/CdS}M$ | [185] |
|  | $\mathrm{PVDF}^{Ag/SiO2}M$ | [196] | $\mathrm{PVDF}^{TiO2/GO}M$ | [72] | $\frac{\text{PA}^{\text{rGO/TiO2/Ag}}}{\text{PES/Si3N4}}M$ | [198] |
|  | $\mathrm{PES}^{\mathrm{NH}2-Fe3O4/SiO2}M$ | [170] | $\mathrm{PVC}^{TiO2/GO}M$ | [3] | $\mathrm{PVDF}^{Ag/ZnO}M$ | [187] |
|  | $\frac{\mathrm{PDA}^{\mathrm{TiO}2/ZnO}}{\mathrm{PA}}M$ | [189] | $\frac{\mathrm{PDA}^{\text{Ag/MoS2/TiO2}}}{\mathrm{PSF}}M$ | [200] | $\mathrm{PES}^{FBN/GO/Ag}M$ | [202] |
|  | $\frac{\mathrm{PA}^{GO-SiO2}}{\mathrm{PSF}}M$ | [102] | $\mathrm{PVDF}^{TiO2/CNT}M$ | [9] | $\frac{\mathrm{PA}^{\text{TiO2/Ag}}}{\mathrm{PES}}M$ | [206] |
|  | $\mathrm{CA}^{Fe3o4/MXene}M$ | [147] | $\mathrm{PVC}^{TiO2/ZnO}M$ | [15] | $\mathrm{PVC}^{TiO2/HNT}M$ | [62] |
|  | $\mathrm{PES}^{Fe3o4/GO}M$ | [138] | $\mathrm{BC}^{TiO2/ZnO}M$ | [193] | $\mathrm{PA}^{TiO2/GO}M$ | [48] |
|  | $\mathrm{PVDF}^{\mathrm{Fe}3O4/HNT}M$ | [141] | $\frac{\mathrm{PA}^{\text{TiO2/CD}}}{\mathrm{PSF}}M$ | [33] | $\mathrm{CA}^{SiO2/GO}M$ | [119] |
|  | $\mathrm{PVC}^{\mathrm{Fe}3O4/Ag}M$ | [144] | $\mathrm{PVDF}^{TiO2/BiVO4/CNT}M$ | [45] | $\mathrm{PAN}^{SiO2/GO}M$ | [104] |
|  | $\mathrm{PES}^{Cu/Fe3o4}M$ | [159] |  |  |  |  |

**Table S*3* Different types of functional groups of nanoparticles based on studies published in during the years 2010-2023**

| **functional groups** | **Membrane** | **Ref** | **Membrane** | **Ref** | **Membrane** | **Ref** |
| --- | --- | --- | --- | --- | --- | --- |
| **Amine** | $\frac{\mathrm{PA}^{Fe-NH2-clay}}{\mathrm{PAN}}M$ | [222] | $\mathrm{PES}^{\mathrm{NH}2-ZiF-8/GO}$ | [236] | $\frac{\mathrm{PA}^{NH2-CQD}}{\mathrm{PSF}}M$ | [253] |
|  | $\frac{\mathrm{polypiperazineamide}^{NH2-ND}}{\mathrm{PA}}M$ | [225] | $\frac{\mathrm{PA}}{\mathrm{PES}^{NH2-ZnO}}$ | [192] | $\mathrm{PES}^{NH2-ZnO}M$ | [179] |
|  | $\frac{\mathrm{PA}^{NH2-CQD}}{\mathrm{PES}}M$ | [228] | $\frac{\mathrm{PA}^{\mathrm{NH}2-UiO-66}}{\mathrm{PI}}M$ | [216] | $\frac{\mathrm{PA}^{\text{NH2-SiO2}}}{PES/Si3N4}M$ | [90] |
|  | $\frac{\mathrm{PVA}^{\text{NH2-ZnO}}}{\mathrm{PC}}M$ | [188] | $\frac{\mathrm{PDA}^{\text{NH2-MWCNT}}}{\mathrm{PES}}M$ | [210] | $\frac{\mathrm{GO}^{NH2-Fe3O4}}{\mathrm{PVDF}}M$ | [157] |
|  | $\frac{\mathrm{PA}^{\text{NH2-SiO2}}}{\mathrm{PES}}M$ | [99] | $\frac{\mathrm{PA}^{\mathrm{NH}2-SiO2/SiC}}{Al2O3}M$ | [126] | $\frac{{chitosan/PVA}^{NH2-Fe3O4}}{\mathrm{PES}}M$ | [166] |
|  | $\frac{\mathrm{PA}^{NH2-GO}}{\mathrm{PES}}M$ | [231] | $\frac{\mathrm{PDA}^{\text{NH2-SiO2}}}{\mathrm{PES}}M$ | [129] | $\mathrm{PES}^{\mathrm{PABFNP}}M$ | [230] |
|  | $\frac{\mathrm{PA}^{\mathrm{NH}2-TiO2}}{\mathrm{PES}}M$ | [4] | $\frac{\mathrm{PA}}{\mathrm{PEI}^{NH2-SiO2}}M$ | [121] | $\frac{\text{PA}^{\text{NH2-rGO/TiO2/Ag}}}{\text{PAN/Si3N4}}M$ | [205] |
|  | $\mathrm{CA}^{\mathrm{NH}2-ND}$ | [247] | $\frac{{PDA/PEI}^{\text{NH2-SiO2}}}{\mathrm{PSF}}M$ | [80] | $\mathrm{PVDF}^{APTES-SiO2/Ag}M$ | [132] |
|  | $\mathrm{CA}^{NH2-SiC}M$ | [217] | $\mathrm{PES}^{NH2-SiO2/COOH-\mathrm{MWCNT}}M$ | [110] | $\mathrm{PES}^{NH2-Fe3o4}M$ | [156] |
|  | $\mathrm{PSf}^{\mathrm{NH}2-\mathrm{SiO}2}M$ | [105] | $\mathrm{PSF}^{\mathrm{NH}2-NC}$ | [248] | ${PES/PVP}^{NH2-Fe3o4}M$ | [171] |
| **Carboxyl** | $\mathrm{PSf}^{\mathrm{COOH}-ZrO2}M$ | [208] | $\mathrm{PVDF}^{OCMCS/Fe3O4}M$ | [167] | $\frac{\mathrm{PA}^{COOH-COF}}{\mathrm{MCE}}M$ | [234] |
|  | $\mathrm{PES}^{\mathrm{COOOH}-\mathrm{Fe}3O4/SiO2}M$ | [155] | $\mathrm{PVDF}^{COOH-MWCNT}M$ | [245] | $\frac{\mathrm{PA}^{COOH-MoS2}}{\mathrm{PES}}M$ | [237] |
|  | $\frac{\mathrm{PA}^{COOH-CQD}}{\mathrm{PSF}}M$ | [253] | $\frac{\mathrm{PA}^{COOH-CN}}{\mathrm{PSF}}M$ | [252] | $\mathrm{CA}^{COOH-ND}M$ | [240] |
|  | $\mathrm{PAN}^{\mathrm{COOH}-ZrO2}M$ | [212] | $\mathrm{PVDF}^{COOH-FO}M$ | [246] | $\mathrm{PES}^{NH2-SiO2/COOH-\mathrm{MWCNT}}M$ | [110] |
|  | $\mathrm{PVDF}^{OCMCS/Fe3O4}M$ | [164] |  |  |  |  |
| **Sulfate** | $\mathrm{PES}^{S-TiO2}M$ | [58] | $\mathrm{PES}^{Sulf-PDA-Fe3O4}M$ | [158] | $\mathrm{PSF}^{S-TiO2/SiO2}M$ | [109] |
|  | $\mathrm{PES}^{SiO2-TiO2}M$ | [61] | $\mathrm{PAN}^{SO4-ZrO2}M$ | [212] | $\mathrm{PES}^{S-TiO2}M$ | [74] |
|  | $\mathrm{PSf}^{SO4-ZrO2}M$ | [208] |  |  |  |  |
| **Other** | $\frac{\mathrm{PA}^{\mathrm{Fluorinatedsilica}}}{\mathrm{PES}}M$ | [108] | $\mathrm{PSf}^{talc-Fe3o4}M$ | [150] | ${polyurethane/CA}^{F-TiO2}M$ | [27] |
|  | $\mathrm{PES}^{MDA-Fe3O4}M$ | [143] | $\mathrm{PES}^{CPTES-TiO2}M$ | [59] | $\mathrm{PES}^{8-HQFe3O4}M$ | [151] |
|  | $\mathrm{PES}^{\mathrm{APTES}-TiO2}M$ | [34] | $\mathrm{HDPE}^{F-SiO2}M$ | [85] | $\mathrm{PES}^{APTES-Fe3O4}M$ | [160] |
|  | $\mathrm{PES}^{F-carbon dots}M$ | [235] | $\frac{\mathrm{PA}^{\text{OA-SiO2}}}{\mathrm{PSF}}M$ | [100] | $\mathrm{PVC}^{GA-Fe3o4}M$ | [162] |
|  | $\mathrm{PES}^{HMDI-TiO2}M$ | [40] | $\frac{\mathrm{PA}^{m\text{-SiO2}}}{\mathrm{PES}}M$ | [107] | $\mathrm{PES}^{silane-ZnO}M$ | [178] |
|  | $\mathrm{PSf}^{S-ND}M$ | [244] | $\mathrm{PVDF}^{\mathrm{APDEMS}-SiC}M$ | [239] | $\frac{\mathrm{PA}^{\text{OA-SiO2}}}{\mathrm{PSF}}M$ | [106] |
|  | $\mathrm{PES}^{metformine-Fe3o4}M$ | [156] | $\frac{\mathrm{PA}^{APTMS-\text{TiO2}}}{\mathrm{PSF}}M$ | [21] | $\mathrm{PES}^{N-Halamine-SiO2}M$ | [124] |
|  | $\mathrm{PVC}^{Fe3O4/O-MWCNT}M$ | [152] |  |  |  |  |

**Table S4 different coatings of nanoparticles based on studies published in during the years 2010-2023**

| **Used coating** | **Membrane** | **Ref** | **Membrane** | **Ref** | **Membrane** | **Ref** |
| --- | --- | --- | --- | --- | --- | --- |
| **PDA** | $\mathrm{PAN}^{PDA-SiO2}M$ | [97] | $\mathrm{PC}^{PDA-TiO2}M$ | [6] | $\mathrm{PES}^{NH2-PDA-Fe3O4}M$ | [158] |
|  | $\mathrm{PVA}^{PDA-SiO2}M$ | [83] | $\frac{\mathrm{PA}}{\mathrm{PES}^{PDA-TiO2}}$ | [30] | $\mathrm{PES}^{PDA-ZnFe2O4}M$ | [250] |
|  | $\mathrm{PVC}^{PDA-Al2O3}M$ | [242] | $\frac{\mathrm{PA}^{\frac{\mathrm{PDA}}{\mathrm{PEG}}@ZIF-8}}{\mathrm{PES}}M$ | [227] |  |  |
| **PANI** | $\mathrm{PVA}^{PANI-TiO2}M$ | [64] | $\mathrm{PVDF}^{PANI-TiO2}M$ | [67] | $\mathrm{PES}^{PANI-Fe3o4}M$ | [173] |
|  | $\mathrm{PES}^{PANi-TiO2}M$ | [35] |  |  |  |  |
| **PEI** | $\mathrm{PES}^{PEI-SiO2}M$ | [101] | $\mathrm{PVDF}^{PEI-SiO2}M$ | [125] | PEI$\mathrm{PSf}^{PEI-SiO2}M$ | [111] |
|  | $\mathrm{PES}^{PEI-SiO2}M$ | [122] | $\frac{\mathrm{PDA}^{PEI-TiO2\_Ag}}{\mathrm{PAN}}M$ | [207] |  |  |
| **Other** | $\frac{{AA}^{AA-CuS}}{\mathrm{PSf}}M$ | [238] | PAN^SA-ZnO^M | [194] | $\mathrm{PVDF}^{XG-Fe3O4}M$ | [140] |
|  | $\mathrm{PES}^{PVP-Fe3o4}M$ | [146] | $\frac{\mathrm{Polyester}^{\text{TA-TiO2}}}{\mathrm{PES}}M$ | [56] | $\mathrm{PVDF}^{PGS-Fe3O4}M$ | [148] |
|  | $\mathrm{PES}^{PVP-DDT-Ag}$ | [199] | $\mathrm{PES}^{PSS-SiO2}M$ | [112] | $\mathrm{PES}^{PAA-Fe3O4}M$ | [172] |
|  | $\mathrm{GO}^{PAA-NH2-UiO-66}M$ | [251] | $\mathrm{PVDF}^{PPy-TiO2}M$ | [12] | $\frac{\mathrm{PA}^{\text{Pal-TiO2}}}{\mathrm{PSf}}M$ | [60] |
|  | $\mathrm{PVDF}^{polyhexanide-\mathrm{CuO}}M$ | [249] | $\mathrm{PVDF}^{PVP-TiO2}M$ | [54] | $\mathrm{CTA}^{L\mathrm{udox}-SiO2}M$ | [92] |

**Table S5 Different types of composite structures of nanoparticles based on studies published in during the years 2010-2023**

| **Types of composite** | **Membrane** | **Ref** | **Membrane** | **Ref** | **Membrane** | **Ref** |
| --- | --- | --- | --- | --- | --- | --- |
| **Core-shell** | $\frac{\mathrm{PA}^{\mathrm{Fe}3O4/ZrO2}}{\mathrm{PAN}^{ZrO2}}$M | [137] | $\mathrm{PES}^{TiO2/Ni}M$ | [53] | $\mathrm{PVDF}^{TiO2/Fe3O4}M$ | [142] |
|  | $\mathrm{PAN}^{\mathrm{Fe}3O4/ZrO2}M$ | [149] | $\mathrm{PSf}^{SiO2/TiO2}M$ | [113] | $\mathrm{PVDF}^{TiO2/Fe3O4}M$ | [154] |
|  | $\mathrm{PES}^{SiO2/Ag}M$ | [204] | $\mathrm{PSF}^{ZnO/MnO2/SiO2}M$ | [190] | $\mathrm{PES}^{Cu/Fe3o4}M$ | [159] |
|  | $\mathrm{PVDF}^{\mathrm{TiO}2/ZrO2}M$ | [19] | $\mathrm{Psf}^{Ag/Cu/SiO2}M$ | [134] | $\frac{\mathrm{PA}^{\text{TiO2/CD}}}{\mathrm{PSF}}M$ | [33] |
|  | $\mathrm{PAN}^{\mathrm{Fe}3O4/ZrO2}M$ | [161] | $\frac{\mathrm{PDA}^{\text{Ag/MoS2/TiO2}}}{\mathrm{PSF}}M$ | [200] |  |  |
| **Hybrid** | $\frac{\mathrm{PA}^{\mathrm{Fe}3O4/ZnO}}{\mathrm{PES}^{\mathrm{Fe}3O4/ZnO}}M$ | [177] | $\frac{\mathrm{PA}^{\mathrm{Ag}}}{\mathrm{PSU}^{\mathrm{MWNT}}}$ | [197] | $\mathrm{PSF}^{Fe3o4/GO}M$ | [168] |
|  | $\mathrm{CA}^{TiO2/Al2O3}M$ | [7] | $\mathrm{PVDF}^{TiO2/HNT}M$ | [44] | $\mathrm{PSF}^{Fe3O4/GO}M$ | [175] |
|  | $\frac{\mathrm{PA}}{\mathrm{PSf}^{TiO2/Ag}}M$ | [201] | $\mathrm{PVDF}^{SiO2/GO}M$ | [82] | $\mathrm{PES}^{Fe3O4/HNC}M$ | [174] |
|  | $\mathrm{PES}^{Fe3O4/MoS2}M$ | [139] | $\mathrm{PES}^{SiO2/GO}M$ | [133] | $\mathrm{PSF}^{Fe3O4/GO}M$ | [145] |
|  | $\mathrm{PES}^{Cu/ZnO}M$ | [183] | $\frac{\mathrm{PDA}^{\text{SiO2/TiO2}}}{\mathrm{PVDF}}M$ | [93] | $\mathrm{PVDF}^{g-C3N4/Fe3O4}M$ | [163] |
|  | $\mathrm{PES}^{MWCNT/ZnO}M$ | [186] | ${PVDF-g-PMMA}^{SiO2/GO}M$ | [116] | $\mathrm{PES}^{ZnO/GO}M$ | [182] |
|  | $\mathrm{PAN}^{\mathrm{Fe}3O4/ZrO2}M$ | [161] | $\mathrm{PVDF}^{\mathrm{SiO}2/Cu}M$ | [131] | $\mathrm{PU}^{ZnO/CdS}M$ | [185] |
|  | $\mathrm{PVDF}^{Ag/SiO2}M$ | [196] | $\mathrm{PVDF}^{TiO2/GO}M$ | [72] | $\frac{\text{PA}^{\text{rGO/TiO2/Ag}}}{\text{PES/Si3N4}}M$ | [198] |
|  | $\frac{\mathrm{PDA}^{\mathrm{TiO}2/ZnO}}{\mathrm{PA}}M$ | [189] | $\mathrm{PVC}^{TiO2/GO}M$ | [3] | $\mathrm{PVDF}^{Ag/ZnO}M$ | [187] |
|  | $\frac{\mathrm{PA}^{GO-SiO2}}{\mathrm{PSF}}M$ | [102] | $\mathrm{PVDF}^{TiO2/CNT}M$ | [9] | $\mathrm{PES}^{FBN/GO/Ag}M$ | [202] |
|  | $\mathrm{PES}^{TiO2/\mathrm{MWCNT}}M$ | [11] | $\mathrm{PVC}^{TiO2/ZnO}M$ | [15] | $\frac{\mathrm{PA}^{\text{TiO2/Ag}}}{\mathrm{PES}}M$ | [206] |
|  | $\mathrm{PES}^{Fe3o4/GO}M$ | [138] | $\mathrm{BC}^{TiO2/ZnO}M$ | [193] | $\mathrm{PVC}^{TiO2/HNT}M$ | [62] |
|  | $\mathrm{PVDF}^{\mathrm{Fe}3O4/HNT}M$ | [141] | $\mathrm{PVDF}^{TiO2/BiVO4/CNT}M$ | [45] | $\mathrm{CA}^{SiO2/GO}M$ | [119] |
|  | $\mathrm{PVC}^{\mathrm{Fe}3O4/Ag}M$ | [144] | $\mathrm{PA}^{TiO2/GO}M$ | [48] | $\mathrm{PAN}^{SiO2/GO}M$ | [104] |
|  | $\mathrm{PES}^{Cu/Fe3O4}M$ | [159] | $\mathrm{PVDF}^{TiO2/Ag}M$ | [203] | $\mathrm{CA}^{Fe3o4/MXene}M$ | [147] |

| Acrylic acid | AA |
| --- | --- |
| 3-aminopropyl(diethoxy)methylsilane | APDEMS |
| 3-aminopropyltriethoxysilane | APTES |
| (3-aminopropyl)trimethoxysilane | APTMS |
| Cellulose acetate | CA |
| Cyanuric chloride | CC |
| Carbon dot | CD |
| Carboxymethyl chitosan | CMC |
| Carbon nanosphere | CNS |
| Carbon nanotube | CNT |
| Covalent organic frameworks | COF |
| Cyanopropyltriethoxysilane | CPTES |
| Carbon quantum dots | CQD |
| Cellulose triacetate | CTA |
| 1-dodecanthiol | DDT |
| Emulsion polyvinyl chloride | EPVC |
| Functionalized hexagonal boron nitride | FBN |
| Gum Arabic | GA |
| Graphene oxide | GO |
| Hexafluoropropylene | HFP |
| High-density polyethylene | HDPE |
| 1, 6-hexamethylene diisocyanate | HMDA |
| Halloysite nanoclay | HNC |
| Halloysite nanotubes | HNT |
| Melamine-based dendrimer amine | MDA |
| Multiwalled carbon nanotube | MWCNT |
| Multi-walled carbon nanotube | MWNT |
| Nanocrystalline cellulose | NC |
| Nanodiamond | ND |
| Naringin | Nar |
| Oleic acid | OA |
| O-carboxymethyl chitosan | OCMCS |
| Polyamide | PA |
| Palygorskite | Pal |
| Para-amino benzoate ferroxane nanoparticle | PABFNP |
| Polyacrylonitrile | PAN |
| Polyaniline | PANI |
| Polydopamine | PDA |
| Phenylene ether-ether sulfone | PEES |
| Polyethylene glycol | PEG |
| Polyethyleneimine | PEI |
| Polyether sulfone | PES |
| Perfluorosulfonic acid | PFSA |
| Palygorskite | PGS |
| Polyamide | PI |
| Poly m-phenylene isophthalamide | PMIA |
| Poly(methyl methacrylate) | PMMA |
| Poly (phthalazine ether sulfone ketone) | PPESK |
| Polystyrene | PS |
| Polysulfone | PSF |
| Sodium 4-styrene sulfonate | PSS |
| Polyvinyl alcohol | PVA |
| Poly vinyl chloride | PVC |
| Polyvinylidene fluoride | PVDF |
| Polyvinylpyrrolidone | PVP |
| Polyphenylsulfone | PPSU |
| Polypyrrole | PPY |
| Reduced graphene oxide | rGO |
| Sodium alginate | SA |
| Silicon carbide | SiC |
| Sulfonated polysulfone | SPSF |
| Tannic acid | TA |
| Xanthan gum | XG |
| 8-hydroxyquinoline | 8-HQ |

[1] B. Rajaeian, A. Heitz, M. O. Tade, and S. Liu, "Improved separation and antifouling performance of PVA thin film nanocomposite membranes incorporated with carboxylated TiO2 nanoparticles," *Journal of Membrane Science,* vol. 485, pp. 48-59, 2015.

[2] S. Zhao, P. Wang, C. Wang, X. Sun, and L. Zhang, "Thermostable PPESK/TiO2 nanocomposite ultrafiltration membrane for high temperature condensed water treatment," *Desalination,* vol. 299, pp. 35-43, 2012.

[3] S. A. Mousa, H. Abdallah, S. Ibrahim, and S. Khairy, "Enhanced photocatalytic properties of graphene oxide/polyvinylchloride membranes by incorporation with green prepared SnO2 and TiO2 nanocomposite for water treatment," *Applied Physics A,* vol. 129, no. 12, p. 831, 2023.

[4] S. Wei *et al.*, "Monovalent/Divalent salts separation via thin film nanocomposite nanofiltration membrane containing aminated TiO2 nanoparticles," *Journal of the Taiwan Institute of Chemical Engineers,* vol. 112, pp. 169-179, 2020.

[5] V. Vatanpour, S. S. Madaeni, A. R. Khataee, E. Salehi, S. Zinadini, and H. A. Monfared, "TiO2 embedded mixed matrix PES nanocomposite membranes: Influence of different sizes and types of nanoparticles on antifouling and performance," *Desalination,* vol. 292, pp. 19-29, 2012.

[6] A. Yousefi, H. Etemadi, and H. Sattari, "Study on the Fabricated PC/PDA-Modified TiO2 Hybrid Membranes for oily wastewater treatment in a submerged membrane system," *Journal of Inorganic and Organometallic Polymers and Materials,* vol. 33, no. 9, pp. 2861-2872, 2023.

[7] J. Baniasadi, Z. Shabani, T. Mohammadi, and S. Sahebi, "Enhanced performance and fouling resistance of cellulose acetate forward osmosis membrane with the spatial distribution of TiO2 and Al2O3 nanoparticles," *Journal of Chemical Technology & Biotechnology,* vol. 96, no. 1, pp. 147-162, 2021.

[8] A. Rahimpour, M. Jahanshahi, A. Mollahosseini, and B. Rajaeian, "Structural and performance properties of UV-assisted TiO2 deposited nano-composite PVDF/SPES membranes," *Desalination,* vol. 285, pp. 31-38, 2012.

[9] L. Fekete *et al.*, "Outstanding separation performance of Oil-in-Water emulsions with TiO2/CNT nanocomposite-modified PVDF membranes," *Membranes,* vol. 13, no. 2, p. 209, 2023.

[10] K. Bouziane Errahmani, O. Benhabiles, S. Bellebia, Z. Bengharez, M. Goosen, and H. Mahmoudi, "Photocatalytic nanocomposite polymer-TiO2 membranes for pollutant removal from wastewater," *Catalysts,* vol. 11, no. 3, p. 402, 2021.

[11] V. Vatanpour, S. S. Madaeni, R. Moradian, S. Zinadini, and B. Astinchap, "Novel antibifouling nanofiltration polyethersulfone membrane fabricated from embedding TiO2 coated multiwalled carbon nanotubes," *Separation and purification technology,* vol. 90, pp. 69-82, 2012.

[12] U. Baig and A. Waheed, "A facile strategy for fabrication of nanocomposite ultrafiltration membrane: oily wastewater treatment and photocatalytic self-cleaning," *npj Clean Water,* vol. 6, no. 1, p. 68, 2023.

[13] J. Zhang, Z. Wang, Q. Wang, C. Pan, and Z. Wu, "Comparison of antifouling behaviours of modified PVDF membranes by TiO2 sols with different nanoparticle size: Implications of casting solution stability," *Journal of Membrane Science,* vol. 525, pp. 378-386, 2017.

[14] S. Pourjafar, A. Rahimpour, and M. Jahanshahi, "Synthesis and characterization of PVA/PES thin film composite nanofiltration membrane modified with TiO2 nanoparticles for better performance and surface properties," *Journal of Industrial and Engineering Chemistry,* vol. 18, no. 4, pp. 1398-1405, 2012.

[15] S. A. Mousa, H. Abdallah, and S. Khairy, "Low-cost photocatalytic membrane modified with green heterojunction TiO2/ZnO nanoparticles prepared from waste," *Scientific Reports,* vol. 13, no. 1, p. 22150, 2023.

[16] J.-F. Li, Z.-L. Xu, H. Yang, L.-Y. Yu, and M. Liu, "Effect of TiO2 nanoparticles on the surface morphology and performance of microporous PES membrane," *Applied Surface Science,* vol. 255, no. 9, pp. 4725-4732, 2009.

[17] A. Razmjou, A. Resosudarmo, R. L. Holmes, H. Li, J. Mansouri, and V. Chen, "The effect of modified TiO2 nanoparticles on the polyethersulfone ultrafiltration hollow fiber membranes," *Desalination,* vol. 287, pp. 271-280, 2012.

[18] M. A. Abu-Dalo, A. Bozeya, Z. Sawalmeh, B. Albiss, N. Alnairat, and R. Abu-Zurayk, "Antifouling polymeric nanocomposite membrane based on interfacial polymerization of polyamide enhanced with green TiO2 nanoparticles for water desalination," *PeerJ Analytical Chemistry,* vol. 5, p. e26, 2023.

[19] Y. Zhang, L. Wang, and Y. Xu, "Effect of doping porous ZrO2 solid superacid shell/void/TiO2 core nanoparticles (ZVT) on properties of polyvinylidene fluoride (PVDF) membranes," *Desalination,* vol. 358, pp. 84-93, 2015.

[20] F. Shi, Y. Ma, J. Ma, P. Wang, and W. Sun, "Preparation and characterization of PVDF/TiO2 hybrid membranes with different dosage of nano-TiO2," *Journal of Membrane Science,* vol. 389, pp. 522-531, 2012.

[21] Y. Zhu, Y. Xu, S. Chen, Y. Zhou, J. Zhu, and G. Chen, "(3-Aminopropyl) trimethoxysilane-Functionalized Titanium Dioxide Thin-Film Nanocomposite Membrane: Enhanced Rejection Performance for Unary and Binary High-Salt Wastewater," *ACS Applied Polymer Materials,* vol. 5, no. 12, pp. 10137-10147, 2023.

[22] M. T. Moghadam *et al.*, "Improved antifouling properties of TiO2/PVDF nanocomposite membranes in UV‐coupled ultrafiltration," *Journal of Applied Polymer Science,* vol. 132, no. 21, 2015.

[23] M. Abdel-Hameed, "Polysulfone/Polyvinyl alcohol thin film nano-composite membranes: synthesis, characterization and application for desalination of saline groundwater," *J. App. Sci. Res,* vol. 8, no. 7, pp. 3811-3822, 2012.

[24] S. H. Salim, R. H. Al-Anbari, and A. Haider, "Polysulfone/TiO2 thin film nanocomposite for commercial ultrafiltration membranes," *Journal of Applied Sciences and Nanotechnology,* vol. 2, no. 1, pp. 80-89, 2022.

[25] K. A. Gebru and C. Das, "Removal of bovine serum albumin from wastewater using fouling resistant ultrafiltration membranes based on the blends of cellulose acetate, and PVP-TiO2 nanoparticles," *Journal of environmental management,* vol. 200, pp. 283-294, 2017.

[26] Y. Teow, B. Ooi, A. Ahmad, and J. Lim, "Mixed-matrix membrane for humic acid removal: influence of different types of TiO2 on membrane morphology and performance," *International Journal of Chemical Engineering and Applications,* vol. 3, no. 6, p. 374, 2012.

[27] A. Ahmad *et al.*, "Novel antibacterial polyurethane and cellulose acetate mixed matrix membrane modified with functionalized TiO2 nanoparticles for water treatment applications," *Chemosphere,* vol. 301, p. 134711, 2022.

[28] H. Rabiee, M. H. D. A. Farahani, and V. Vatanpour, "Preparation and characterization of emulsion poly (vinyl chloride)(EPVC)/TiO2 nanocomposite ultrafiltration membrane," *Journal of Membrane Science,* vol. 472, pp. 185-193, 2014.

[29] L. Liu, C. Zhao, and F. Yang, "TiO2 and polyvinyl alcohol (PVA) coated polyester filter in bioreactor for wastewater treatment," *Water research,* vol. 46, no. 6, pp. 1969-1978, 2012.

[30] P. Kallem, R. P. Pandey, H. M. Hegab, R. Gaur, S. W. Hasan, and F. Banat, "High-performance thin-film composite forward osmosis membranes with hydrophilic PDA@ TiO2 nanocomposite substrate for the treatment of oily wastewater under PRO mode," *Journal of Environmental Chemical Engineering,* vol. 10, no. 3, p. 107454, 2022.

[31] S. D. Neelapala, A. K. Nair, and P. JagadeeshBabu, "Synthesis and characterisation of TiO2 nanofibre/cellulose acetate nanocomposite ultrafiltration membrane," *Journal of Experimental Nanoscience,* vol. 12, no. 1, pp. 152-165, 2017.

[32] T. Al-Jadir *et al.*, "Fabrication and characterization of polyphenylsulfone/titanium oxide nanocomposite membranes for oily wastewater treatment," *Journal of Ecological Engineering,* vol. 23, no. 12, 2022.

[33] V. Vatanpour, S. Paziresh, S. A. N. Mehrabani, S. Feizpoor, A. Habibi-Yangjeh, and I. Koyuncu, "TiO2/CDs modified thin-film nanocomposite polyamide membrane for simultaneous enhancement of antifouling and chlorine-resistance performance," *Desalination,* vol. 525, p. 115506, 2022.

[34] A. Razmjou, J. Mansouri, and V. Chen, "The effects of mechanical and chemical modification of TiO2 nanoparticles on the surface chemistry, structure and fouling performance of PES ultrafiltration membranes," *Journal of Membrane Science,* vol. 378, no. 1-2, pp. 73-84, 2011.

[35] A. P. Mojdehi, M. P. Chenar, M. Namvar-Mahboub, and M. Eftekhari, "Development of PES/polyaniline-modified TiO2 adsorptive membrane for copper removal," *Colloids and Surfaces A: Physicochemical and Engineering Aspects,* vol. 583, p. 123931, 2019.

[36] H. Jain, A. Verma, R. Dhupper, S. Wadhwa, and M. Garg, "Development of CA-TiO2-incorporated thin-film nanocomposite forward osmosis membrane for enhanced water flux and salt rejection," *International Journal of Environmental Science and Technology,* vol. 19, no. 6, pp. 5387-5400, 2022.

[37] A. Akbari and M. Homayoonfal, "Sulfonation and mixing with TiO2 nanoparticles as two simultaneous solutions for reducing fouling of polysulfone loose nanofiltration membrane," *Korean Journal of Chemical Engineering,* journal article vol. 33, no. 8, pp. 2439-2452, August 01 2016, doi: 10.1007/s11814-016-0107-5.

[38] P. Kedchaikulrat, I. F. Vankelecom, K. Faungnawakij, and C. Klaysom, "Effects of colloidal TiO2 and additives on the interfacial polymerization of thin film nanocomposite membranes," *Colloids and Surfaces A: Physicochemical and Engineering Aspects,* vol. 601, p. 125046, 2020.

[39] M. Dmitrenko *et al.*, "Modification strategies of polyacrylonitrile ultrafiltration membrane using TiO2 for enhanced antifouling performance in water treatment," *Separation and Purification Technology,* vol. 286, p. 120500, 2022.

[40] L. Zhang, H. Guan, N. Zhang, B. Jiang, Y. Sun, and N. Yang, "A loose NF membrane by grafting TiO2-HMDI nanoparticles on PES/β-CD substrate for dye/salt separation," *Separation and Purification Technology,* vol. 218, pp. 8-19, 2019.

[41] H. Etemadi, M. Fonouni, and R. Yegani, "Investigation of antifouling properties of polypropylene/TiO2 nanocomposite membrane under different aeration rate in membrane bioreactor system," *Biotechnology Reports,* vol. 25, p. e00414, 2020.

[42] V. Vatanpour, M. Hazrati, M. Sheydaei, and A. Dehqan, "Investigation of using UV/H2O2 pre-treatment process on filterability and fouling reduction of PVDF/TiO2 nanocomposite ultrafiltration membrane," *Chemical Engineering and Processing-Process Intensification,* vol. 170, p. 108677, 2022.

[43] W. Hu, J. Yin, B. Deng, and Z. Hu, "Application of nano TiO2 modified hollow fiber membranes in algal membrane bioreactors for high-density algae cultivation and wastewater polishing," *Bioresource technology,* vol. 193, pp. 135-141, 2015.

[44] A. Moslehyani, R. Farnood, S. Tabe, T. Matsuura, and A. F. Ismail, "Novel nanocomposite HNT-TiO2/PVDF adsorptive nanofiber membranes for arsenic (III) removal," *Journal of Membrane Science and Research,* vol. 6, no. 4, pp. 416-423, 2020.

[45] E. J. Sisay *et al.*, "Visible-light-driven photocatalytic PVDF-TiO2/CNT/BiVO4 hybrid nanocomposite ultrafiltration membrane for dairy wastewater treatment," *Chemosphere,* vol. 307, p. 135589, 2022.

[46] D. Emadzadeh, W. J. Lau, T. Matsuura, M. Rahbari-Sisakht, and A. F. Ismail, "A novel thin film composite forward osmosis membrane prepared from PSf–TiO2 nanocomposite substrate for water desalination," *Chemical Engineering Journal,* vol. 237, pp. 70-80, 2014.

[47] H. Etemadi, A. Amirjangi, N. Ghasemian, and E. Shokri, "Synthesis and characterization of polycarbonate/TiO2 ultrafiltration membranes: critical flux determination," *Chemical Engineering & Technology,* vol. 43, no. 11, pp. 2247-2258, 2020.

[48] A. Q. Al-Gamal, W. S. Falath, and T. A. Saleh, "Enhanced efficiency of polyamide membranes by incorporating TiO2-Graphene oxide for water purification," *Journal of Molecular Liquids,* vol. 323, p. 114922, 2021.

[49] A. Yadav, K. Singh, A. B. Panda, P. K. Labhasetwar, and V. K. Shahi, "Membrane distillation crystallization for simultaneous recovery of water and salt from tannery industry wastewater using TiO2 modified poly (vinylidene fluoride-co-hexafluoropropylene) nanocomposite membranes," *Journal of Water Process Engineering,* vol. 44, p. 102393, 2021.

[50] Z. Yu, H. Zeng, X. Min, and X. Zhu, "High‐performance composite photocatalytic membrane based on titanium dioxide nanowire/graphene oxide for water treatment," *Journal of Applied Polymer Science,* vol. 137, no. 12, p. 48488, 2020.

[51] Q. Zhong, G. Shi, Q. Sun, P. Mu, and J. Li, "Robust PVA-GO-TiO2 composite membrane for efficient separation oil-in-water emulsions with stable high flux," *Journal of Membrane Science,* vol. 640, p. 119836, 2021.

[52] T. D. Kusworo, N. Ariyanti, and D. P. Utomo, "Effect of nano-TiO2 loading in polysulfone membranes on the removal of pollutant following natural-rubber wastewater treatment," *Journal of Water Process Engineering,* vol. 35, p. 101190, 2020.

[53] T. Sun *et al.*, "Magnetic field assisted arrangement of photocatalytic TiO2 particles on membrane surface to enhance membrane antifouling performance for water treatment," *Journal of colloid and interface science,* vol. 570, pp. 273-285, 2020.

[54] H. Rafiei, M. Abbasian, and R. Yegani, "Polyvinylidene fluoride as a neat and the synthesized novel membranes based on PVDF/polyvinyl pyrrolidone polymer grafted with TiO2 nanoparticles through RAFT method for water purification," *Iranian Polymer Journal,* vol. 30, no. 8, pp. 769-780, 2021.

[55] M. Huang, Y. Chen, C.-H. Huang, P. Sun, and J. Crittenden, "Rejection and adsorption of trace pharmaceuticals by coating a forward osmosis membrane with TiO2," *Chemical Engineering Journal,* vol. 279, pp. 904-911, 2015.

[56] T. Li *et al.*, "In-situ coating TiO2 surface by plant-inspired tannic acid for fabrication of thin film nanocomposite nanofiltration membranes toward enhanced separation and antibacterial performance," *Journal of colloid and interface science,* vol. 572, pp. 114-121, 2020.

[57] N. Mahdi, P. Kumar, A. Goswami, B. Perdicakis, K. Shankar, and M. Sadrzadeh, "Robust polymer nanocomposite membranes incorporating discrete TiO2 nanotubes for water treatment," *Nanomaterials,* vol. 9, no. 9, p. 1186, 2019.

[58] S. Ayyaru and Y.-H. Ahn, "Fabrication and separation performance of polyethersulfone/sulfonated TiO 2 (PES–STiO 2) ultrafiltration membranes for fouling mitigation," *Journal of Industrial and Engineering Chemistry,* 2018.

[59] V. Barahimi, R. A. Taheri, A. Mazaheri, and H. Moghimi, "Fabrication of a novel antifouling TiO 2/CPTES/metformin-PES nanocomposite membrane for removal of various organic pollutants and heavy metal ions from wastewater," *Chemical Papers,* vol. 74, pp. 3545-3556, 2020.

[60] T. Zhang, Z. Li, W. Wang, Y. Wang, B. Gao, and Z. Wang, "Enhanced antifouling and antimicrobial thin film nanocomposite membranes with incorporation of Palygorskite/titanium dioxide hybrid material," *Journal of colloid and interface science,* vol. 537, pp. 1-10, 2019.

[61] J. Guo and J. Kim, "Modifications of polyethersulfone membrane by doping sulfated-TiO 2 nanoparticles for improving anti-fouling property in wastewater treatment," *RSC Advances,* vol. 7, no. 54, pp. 33822-33828, 2017.

[62] G. Mishra and M. Mukhopadhyay, "TiO2 decorated functionalized halloysite nanotubes (TiO2@ HNTs) and photocatalytic PVC membranes synthesis, characterization and its application in water treatment," *Scientific reports,* vol. 9, no. 1, pp. 1-17, 2019.

[63] Y. Orooji, E. Ghasali, N. Emami, F. Noorisafa, and A. Razmjou, "ANOVA design for the optimization of TiO2 coating on polyether sulfone membranes," *Molecules,* vol. 24, no. 16, p. 2924, 2019.

[64] T. M. Aminabhavi and M. B. Patil, "Nanocomposite membranes of poly (vinyl alcohol) loaded with polyaniline-coated TiO2 and TiO2 nanoparticles for the pervaporation dehydration of aqueous mixtures of 1, 4-dioxane and tetrahydrofuran," *Designed Monomers and Polymers,* vol. 13, no. 6, pp. 497-508, 2010.

[65] B. Al-Ghafri, W.-J. Lau, M. Al-Abri, P.-S. Goh, and A. F. Ismail, "Titanium dioxide-modified polyetherimide nanofiber membrane for water treatment," *Journal of Water Process Engineering,* vol. 32, p. 100970, 2019.

[66] R. Sharma, P. Galav, and H. D. Raval, "High performance, low‐fouling nanocomposite membrane with TiO2 nanomaterial in polysulfone matrix for advanced water treatment," *Journal of Applied Polymer Science,* vol. 140, no. 14, p. e53662, 2023.

[67] H. Nawaz *et al.*, "Photodegradation of textile pollutants by nanocomposite membranes of polyvinylidene fluoride integrated with polyaniline–titanium dioxide nanotubes," *Chemical Engineering Journal,* vol. 419, p. 129542, 2021.

[68] S. S. Hosseini, S. Fakharian Torbati, M. A. Alaei Shahmirzadi, and T. Tavangar, "Fabrication, characterization, and performance evaluation of polyethersulfone/TiO2 nanocomposite ultrafiltration membranes for produced water treatment," *Polymers for Advanced Technologies,* vol. 29, no. 10, pp. 2619-2631, 2018.

[69] E. S. Mansor, H. Abdallah, and A. Shaban, "Development of TiO2/polyvinyl alcohol-cellulose acetate nanocomposite reverse osmosis membrane for groundwater-surface water interfaces purification," *Materials Science and Engineering: B,* vol. 289, p. 116222, 2023.

[70] A. Rahimpour, M. Jahanshahi, B. Rajaeian, and M. Rahimnejad, "TiO2 entrapped nano-composite PVDF/SPES membranes: Preparation, characterization, antifouling and antibacterial properties," *Desalination,* vol. 278, no. 1-3, pp. 343-353, 2011.

[71] N. Cheshomi, M. Pakizeh, and M. Namvar‐Mahboub, "Preparation and characterization of TiO2/Pebax/(PSf‐PES) thin film nanocomposite membrane for humic acid removal from water," *Polymers for Advanced Technologies,* vol. 29, no. 4, pp. 1303-1312, 2018.

[72] X. Yue, X. Ji, H. Xu, B. Yang, M. Wang, and Y. Yang, "Performance investigation on GO-TiO2/PVDF composite ultrafiltration membrane for slightly polluted ground water treatment," *Energy,* vol. 273, p. 127215, 2023.

[73] M. Batool, A. Shafeeq, B. Haider, and N. M. Ahmad, "TiO2 nanoparticle filler-based mixed-matrix PES/CA nanofiltration membranes for enhanced desalination," *Membranes,* vol. 11, no. 6, p. 433, 2021.

[74] R. Pandiyan, S. Ayyaru, and Y.-H. Ahn, "Non-toxic properties of TiO2 and STiO2 nanocomposite PES ultrafiltration membranes for application in membrane-based environmental biotechnology," *Ecotoxicology and Environmental Safety,* vol. 158, pp. 248-255, 2018.

[75] L. Zhou *et al.*, "Constructing dual-defense mechanisms on membrane surfaces by synergy of PFSA and SiO2 nanoparticles for persistent antifouling performance," *Applied Surface Science,* vol. 440, pp. 113-124, 2018.

[76] L. Jin *et al.*, "Preparation and characterization of a novel PA-SiO2 nanofiltration membrane for raw water treatment," *Desalination,* vol. 298, pp. 34-41, 2012.

[77] A. Dehban, A. Kargari, and F. Z. Ashtiani, "Preparation and optimization of antifouling PPSU/PES/SiO2 nanocomposite ultrafiltration membranes by VIPS-NIPS technique," *Journal of Industrial and Engineering Chemistry,* vol. 88, pp. 292-311, 2020.

[78] M. E. El-Naggar *et al.*, "Experimental and theoretical investigations on fouling resistant cellulose acetate/SiO2 NPs/PEDOT ultrafiltration nanocomposite membranes," *Journal of Cleaner Production,* vol. 324, p. 129288, 2021.

[79] M. Amini, H. Etemadi, A. Akbarzadeh, and R. Yegani, "Preparation and performance evaluation of high-density polyethylene/silica nanocomposite membranes in membrane bioreactor system," *Biochemical Engineering Journal,* vol. 127, pp. 196-205, 2017.

[80] R. C. Puerari, R. A. Gonçalves, N. M. Justino, D. S. Vicentini, and W. G. Matias, "The influence of amine-functionalized SiO2 nanostructures upon nanofiltration membranes," *Environmental nanotechnology, monitoring & management,* vol. 13, p. 100287, 2020.

[81] S. A. Hosseini, M. Vossoughi, N. M. Mahmoodi, and M. Sadrzadeh, "Efficient dye removal from aqueous solution by high-performance electrospun nanofibrous membranes through incorporation of SiO2 nanoparticles," *Journal of Cleaner Production,* vol. 183, pp. 1197-1206, 2018.

[82] Z. Zhu *et al.*, "Improving the hydrophilic and antifouling properties of polyvinylidene fluoride membrane by incorporation of novel nanohybrid GO@ SiO2 particles," *Chemical Engineering Journal,* vol. 314, pp. 266-276, 2017.

[83] H. Yin, J. Zhao, Y. Li, L. Huang, H. Zhang, and L. Chen, "A novel Pd decorated polydopamine-SiO2/PVA electrospun nanofiber membrane for highly efficient degradation of organic dyes and removal of organic chemicals and oils," *Journal of cleaner production,* vol. 275, p. 122937, 2020.

[84] L.-Y. Yu, Z.-L. Xu, H.-M. Shen, and H. Yang, "Preparation and characterization of PVDF–SiO2 composite hollow fiber UF membrane by sol–gel method," *Journal of Membrane Science,* vol. 337, no. 1-2, pp. 257-265, 2009.

[85] A. Akbari, R. Yegani, B. Pourabbas, and A. Behboudi, "Analysis of antifouling behavior of high dispersible hydrophilic poly (ethylene glycol)/vinyl functionalized SiO2 nanoparticles embedded polyethylene membrane," *Desalination and Water Treatment,* vol. 76, pp. 83-97, 2017.

[86] B. Khan, S. Haider, R. Khurram, Z. Wang, and X. Wang, "Preparation of an ultrafiltration (UF) membrane with narrow and uniform pore size distribution via etching of SiO2 nano-particles in a membrane matrix," *Membranes,* vol. 10, no. 7, p. 150, 2020.

[87] Z. Yu, X. Liu, F. Zhao, X. Liang, and Y. Tian, "Fabrication of a low‐cost nano‐SiO2/PVC composite ultrafiltration membrane and its antifouling performance," *Journal of Applied Polymer Science,* vol. 132, no. 2, 2015.

[88] N. Rakhshan and M. Pakizeh, "The effect of functionalized SiO2 nanoparticles on the morphology and triazines separation properties of cellulose acetate membranes," *Journal of Industrial and Engineering Chemistry,* vol. 34, pp. 51-60, 2016.

[89] S. Căprărescu, C. Modrogan, V. Purcar, A. M. Dăncilă, and O. D. Orbuleț, "Study of polyvinyl alcohol-SiO2 nanoparticles polymeric membrane in wastewater treatment containing zinc ions," *Polymers,* vol. 13, no. 11, p. 1875, 2021.

[90] H. Abadikhah, E. N. Kalali, S. Behzadi, S. A. Khan, X. Xu, and S. Agathopoulos, "Amino functionalized silica nanoparticles incorporated thin film nanocomposite membrane with suppressed aggregation and high desalination performance," *Polymer,* vol. 154, pp. 200-209, 2018.

[91] M. Obaid, Z. K. Ghouri, O. A. Fadali, K. A. Khalil, A. A. Almajid, and N. A. Barakat, "Amorphous SiO2 NP-incorporated poly (vinylidene fluoride) electrospun nanofiber membrane for high flux forward osmosis desalination," *ACS applied materials & interfaces,* vol. 8, no. 7, pp. 4561-4574, 2016.

[92] I. Prihatiningtyas, Y. Hartanto, M. S. R. Ballesteros, and B. Van der Bruggen, "Cellulose triacetate/LUDOX‐SiO2 nanocomposite for synthesis of pervaporation desalination membranes," *Journal of Applied Polymer Science,* vol. 138, no. 11, p. 50000, 2021.

[93] M. Safarnia, M. Pakizeh, and M. Namvar-Mahboub, "Assessment of structural and separation properties of a PVDF/PD composite membrane incorporated with TiO2 nanotubes and SiO2 particles," *Industrial & Engineering Chemistry Research,* vol. 60, no. 1, pp. 659-669, 2020.

[94] M. R. Jamalludin *et al.*, "Antifouling polysulfone membranes blended with green SiO2 from rice husk ash (RHA) for humic acid separation," *Chemical Engineering Research and Design,* vol. 114, pp. 268-279, 2016.

[95] Q. Xu, Y. Chen, T. Xiao, and X. Yang, "A facile method to control pore structure of PVDF/SiO2 composite membranes for efficient oil/water purification," *Membranes,* vol. 11, no. 11, p. 803, 2021.

[96] N. Niksefat, M. Jahanshahi, and A. Rahimpour, "The effect of SiO2 nanoparticles on morphology and performance of thin film composite membranes for forward osmosis application," *Desalination,* vol. 343, pp. 140-146, 2014.

[97] B. P. Tripathi, N. C. Dubey, R. Subair, S. Choudhury, and M. Stamm, "Enhanced hydrophilic and antifouling polyacrylonitrile membrane with polydopamine modified silica nanoparticles," *RSC advances,* vol. 6, no. 6, pp. 4448-4457, 2016.

[98] M. E. Ali, A. Shahat, T. I. Ayoub, and R. M. Kamel, "Fabrication of high flux polysulfone/mesoporous silica nanocomposite ultrafiltration membranes for industrial wastewater treatment," *Biointerface Res. Appl. Chem,* vol. 12, pp. 7556-7572, 2022.

[99] A. Shakeri, R. Razavi, H. Salehi, M. Fallahi, and T. Eghbalazar, "Thin film nanocomposite forward osmosis membrane embedded with amine-functionalized ordered mesoporous silica," *Applied Surface Science,* vol. 481, pp. 811-818, 2019.

[100] N. Rakhshan and M. Pakizeh, "Removal of triazines from water using a novel OA modified SiO2/PA/PSf nanocomposite membrane," *Separation and purification technology,* vol. 147, pp. 245-256, 2015.

[101] D. A. H. Al-Timimi, Q. F. Alsalhy, A. A. AbdulRazak, and E. Drioli, "Novel polyether sulfone/polyethylenimine grafted nano-silica nanocomposite membranes: Interaction mechanism and ultrafiltration performance," *Journal of Membrane Science,* vol. 659, p. 120784, 2022.

[102] Y. Liu *et al.*, "Synthesis of novel high flux thin-film nanocomposite nanofiltration membranes containing GO–SiO2 via interfacial polymerization," *Industrial & Engineering Chemistry Research,* vol. 58, no. 49, pp. 22324-22333, 2019.

[103] A. Peyki, A. Rahimpour, and M. Jahanshahi, "Preparation and characterization of thin film composite reverse osmosis membranes incorporated with hydrophilic SiO2 nanoparticles," *Desalination,* vol. 368, pp. 152-158, 2015.

[104] F. Ebrahimi, S. R. Nabavi, and A. Omrani, "Fabrication of hydrophilic special sandwich structure of PAN/GO/SiO2 electrospun membrane decorated with SiO2 nanoparticles for oil/water separation," *Journal of Water Process Engineering,* vol. 48, p. 102926, 2022.

[105] X. Li, A. Janke, P. Formanek, A. Fery, M. Stamm, and B. P. Tripathi, "One pot preparation of polysulfone-amino functionalized SiO 2 nanoparticle ultrafiltration membranes for water purification," *Journal of Environmental Chemical Engineering,* 2018.

[106] N. Rakhshan and M. Pakizeh, "The effect of chemical modification of SiO 2 nanoparticles on the nanofiltration characteristics of polyamide membrane," *Korean Journal of Chemical Engineering,* vol. 32, pp. 2524-2533, 2015.

[107] A. Q. Al-Gamal, M. Satria, F. I. Alghunaimi, N. W. Aljuryyed, and T. A. Saleh, "Synthesis of thin-film nanocomposite membranes using functionalized silica nanoparticles for water desalination with drastically improved properties," *Reactive and Functional Polymers,* vol. 181, p. 105433, 2022.

[108] R. Pang and K. Zhang, "Fabrication of hydrophobic fluorinated silica-polyamide thin film nanocomposite reverse osmosis membranes with dramatically improved salt rejection," *Journal of colloid and interface science,* vol. 510, pp. 127-132, 2018.

[109] Y. Zhang and P. Liu, "Polysulfone (PSF) composite membrane with micro-reaction locations (MRLs) made by doping sulfated TiO2 deposited on SiO2 nanotubes (STSNs) for cleaning wastewater," *Journal of Membrane Science,* vol. 493, pp. 275-284, 2015.

[110] H. M. Hegab *et al.*, "Designing of amino silica covalently functionalized carboxylic multi-wall carbon nanotubes-based polyethersulfone membranes for enhancing oily wastewater treatment," *Journal of Environmental Chemical Engineering,* vol. 10, no. 6, p. 108667, 2022.

[111] V. Vatanpour *et al.*, "Hyperbranched polyethylenimine functionalized silica/polysulfone nanocomposite membranes for water purification," *Chemosphere,* vol. 290, p. 133363, 2022.

[112] L. Xing, N. Guo, Y. Zhang, H. Zhang, and J. Liu, "A negatively charged loose nanofiltration membrane by blending with poly (sodium 4-styrene sulfonate) grafted SiO2 via SI-ATRP for dye purification," *Separation and Purification Technology,* vol. 146, pp. 50-59, 2015.

[113] F. Dalanta, T. D. Kusworo, and N. Aryanti, "Synthesis, characterization, and performance evaluation of UV light-driven Co-TiO2@ SiO2 based photocatalytic nanohybrid polysulfone membrane for effective treatment of petroleum refinery wastewater," *Applied Catalysis B: Environmental,* vol. 316, p. 121576, 2022.

[114] J. Li, L. Cheng, W. Song, Y. Xu, F. Liu, and Z. Wang, "In-situ sol-gel generation of SiO2 nanoparticles inside polyamide membrane for enhanced nanofiltration," *Desalination,* vol. 540, p. 115981, 2022.

[115] M. B. Ghandashtani, F. Z. Ashtiani, M. Karimi, and A. Fouladitajar, "A novel approach to fabricate high performance nano-SiO2 embedded PES membranes for microfiltration of oil-in-water emulsion," *Applied Surface Science,* vol. 349, pp. 393-402, 2015.

[116] H. Mahdavi, M. A. Kerachian, and M. Abazari, "Synergistic effect of GO@ SiO2 and GO@ ZnO nano-hybrid particles with PVDF-g-PMMA copolymer in high-flux ultrafiltration membrane for oily wastewater treatment and antifouling properties," *Journal of Industrial and Engineering Chemistry,* vol. 108, pp. 374-388, 2022.

[117] G. N. Manikandan, A. Janani, and M. Helen Kalavathy, "Effect of SiO2 on PSF/PF127 nanocomposite mixed matrix membrane for the separation of oil–water emulsion," *Chemical Papers,* vol. 77, no. 6, pp. 3093-3110, 2023.

[118] H. Wu, B. Tang, and P. Wu, "Development of novel SiO2–GO nanohybrid/polysulfone membrane with enhanced performance," *Journal of Membrane Science,* vol. 451, pp. 94-102, 2014.

[119] Y. Liu *et al.*, "A multifunctional hierarchical porous SiO2/GO membrane for high efficiency oil/water separation and dye removal," *Carbon,* vol. 160, pp. 88-97, 2020.

[120] M. R. S. Kebria, M. Jahanshahi, and A. Rahimpour, "SiO2 modified polyethyleneimine-based nanofiltration membranes for dye removal from aqueous and organic solutions," *Desalination,* vol. 367, pp. 255-264, 2015.

[121] M. Namvar-Mahboub and M. Pakizeh, "Development of a novel thin film composite membrane by interfacial polymerization on polyetherimide/modified SiO2 support for organic solvent nanofiltration," *Separation and Purification Technology,* vol. 119, pp. 35-45, 2013.

[122] D. D. Al-Araji, F. H. Al-Ani, and Q. F. Alsalhy, "Modification of polyethersulfone membranes by Polyethyleneimine (PEI) grafted Silica nanoparticles and their application for textile wastewater treatment," *Environmental Technology,* vol. 44, no. 20, pp. 3033-3049, 2023.

[123] L. Jin *et al.*, "Synthesis of a novel composite nanofiltration membrane incorporated SiO2 nanoparticles for oily wastewater desalination," *Polymer,* vol. 53, no. 23, pp. 5295-5303, 2012.

[124] H. Yu, X. Zhang, Y. Zhang, J. Liu, and H. Zhang, "Development of a hydrophilic PES ultrafiltration membrane containing SiO2@ N-Halamine nanoparticles with both organic antifouling and antibacterial properties," *Desalination,* vol. 326, pp. 69-76, 2013.

[125] L. Teng, C. Yue, and G. Zhang, "Epoxied SiO2 nanoparticles and polyethyleneimine (PEI) coated polyvinylidene fluoride (PVDF) membrane for improved oil water separation, anti-fouling, dye and heavy metal ions removal capabilities," *Journal of Colloid and Interface Science,* vol. 630, pp. 416-429, 2023.

[126] U. Baig and A. Waheed, "Facile fabrication of ceramic-polymeric nanocomposite membrane with special surface wettability using amino decorated NH2-SiO2@ SiC nanopowder for production of clean water from oily wastewater," *Process Safety and Environmental Protection,* vol. 171, pp. 694-704, 2023.

[127] A. Ahmad, M. Majid, and B. Ooi, "Functionalized PSf/SiO2 nanocomposite membrane for oil-in-water emulsion separation," *Desalination,* vol. 268, no. 1-3, pp. 266-269, 2011.

[128] B. Sasikumar, S. G. Krishnan, M. Afnas, G. Arthanareeswaran, P. Goh, and A. Ismail, "A comprehensive performance comparison on the impact of MOF-71, HNT, SiO2, and activated carbon nanomaterials in polyetherimide membranes for treating oil-in-water contaminants," *Journal of Environmental Chemical Engineering,* vol. 11, no. 1, p. 109010, 2023.

[129] E. Bonyadi, F. Z. Ashtiani, S. Ghorabi, and A. S. Niknejad, "Bio-inspired hybrid coating of microporous polyethersulfone membranes by one-step deposition of polydopamine embedded with amino-functionalized SiO2 for high-efficiency oily wastewater treatment," *Journal of Environmental Chemical Engineering,* vol. 10, no. 1, p. 107121, 2022.

[130] M. Shakak *et al.*, "Synthesis and characterization of nanocomposite ultrafiltration membrane (PSF/PVP/SiO2) and performance evaluation for the removal of amoxicillin from aqueous solutions," *Environmental Technology & Innovation,* vol. 17, p. 100529, 2020.

[131] S. Amani, H. Nourizadeh Kazerouni, S. Mikaeili Khiavi, A. Akbari, and R. Yegani, "Improving the Antibiofouling and Operational Properties of PVDF Membranes Using Synthesized Cu‐SiO2 Nanoparticles in a Submerged Membrane Bioreactor," *Chemical Engineering & Technology,* vol. 46, no. 10, pp. 2200-2207, 2023.

[132] Z. Yu, X. Min, F. Li, and Q. Chen, "Synthesis of Ag–SiO2–APTES Nanocomposites by blending poly (Vinylidene Fluoride) Membrane with potential applications on dye wastewater treatment," *Nano,* vol. 13, no. 04, p. 1850034, 2018.

[133] M. B. Alkindy, V. Naddeo, F. Banat, and S. W. Hasan, "Synthesis of polyethersulfone (PES)/GO-SiO2 mixed matrix membranes for oily wastewater treatment," *Water Science and Technology,* vol. 81, no. 7, pp. 1354-1364, 2020.

[134] A. H. Sadek *et al.*, "Polysulfone-based mixed matrix membranes loaded with a multifunctional hierarchical porous Ag-Cu dendrites@ SiO2 core-shell nanostructure for wastewater treatment," *Process Safety and Environmental Protection,* vol. 175, pp. 677-691, 2023.

[135] J. Cui *et al.*, "Bio-inspired fabrication of superhydrophilic nanocomposite membrane based on surface modification of SiO2 anchored by polydopamine towards effective oil-water emulsions separation," *Separation and Purification Technology,* vol. 209, pp. 434-442, 2019.

[136] Y.-c. Du *et al.*, "Preparation of graphene oxide/silica hybrid composite membranes and performance studies in water treatment," *Journal of Materials Science,* vol. 55, pp. 11188-11202, 2020.

[137] R. Karimi, M. Homayoonfal, and F. Davar, "Where is the best site for loading nanoparticles in a membrane? To achieve a high flux and cephalexin separation simultaneously," *Journal of Water Process Engineering,* vol. 38, p. 101578, 2020.

[138] M. Mirzaei, T. Mohammadi, N. Kasiri, and M. A. Tofighy, "Fabrication of magnetic field induced mixed matrix membranes containing GO/Fe3O4 nanohybrids with enhanced antifouling properties for wastewater treatment applications," *Journal of Environmental Chemical Engineering,* vol. 9, no. 4, p. 105675, 2021.

[139] N. Kong, C. Chen, Q. Zeng, B. Li, L. Shen, and H. Lin, "Enriching Fe3O4@ MoS2 composites in surface layer to fabricate polyethersulfone (PES) composite membrane: the improved performance and mechanisms," *Separation and Purification Technology,* vol. 302, p. 122178, 2022.

[140] I. Koyuncu *et al.*, "Modification of PVDF membranes by incorporation Fe3O4@ Xanthan gum to improve anti-fouling, anti-bacterial, and separation performance," *Journal of Environmental Chemical Engineering,* vol. 10, no. 3, p. 107784, 2022.

[141] X. Liu, Y. Chen, Z. Deng, and Y. Yang, "High-performance nanofiltration membrane for dyes removal: Blending Fe3O4-HNTs nanocomposites into poly (vinylidene fluoride) matrix," *Journal of Dispersion Science and Technology,* vol. 42, no. 1, pp. 93-102, 2020.

[142] Y. Cui, J. Zheng, Z. Wang, B. Li, Y. Yan, and M. Meng, "Magnetic induced fabrication of core-shell structure Fe3O4@ TiO2 photocatalytic membrane: Enhancing photocatalytic degradation of tetracycline and antifouling performance," *Journal of Environmental Chemical Engineering,* vol. 9, no. 6, p. 106666, 2021.

[143] H. Koulivand, A. Shahbazi, and V. Vatanpour, "Fabrication and characterization of a high-flux and antifouling polyethersulfone membrane for dye removal by embedding Fe3O4-MDA nanoparticles," *Chemical Engineering Research and Design,* vol. 145, pp. 64-75, 2019.

[144] S. Jahankhah, M. M. Sabzehmeidani, M. Ghaedi, K. Dashtian, and H. Abbasi-Asl, "Fabrication polyvinyl chloride mixed matrix membrane via embedding Fe3O4/polydopamine/Ag nanocomposite for water treatment," *Materials Science and Engineering: B,* vol. 285, p. 115935, 2022.

[145] P. Chai, J. Law, E. Mahmoudi, and A. Mohammad, "Development of iron oxide decorated graphene oxide (Fe3O4/GO) PSf mixed-matrix membrane for enhanced antifouling behavior," *Journal of Water Process Engineering,* vol. 38, p. 101673, 2020.

[146] S. Hosseini *et al.*, "Mixed matrix PES-based nanofiltration membrane decorated by (Fe3O4–polyvinylpyrrolidone) composite nanoparticles with intensified antifouling and separation characteristics," *Chemical Engineering Research and Design,* vol. 147, pp. 390-398, 2019.

[147] X. Yang *et al.*, "Construction of Fe3O4@ MXene composite nanofiltration membrane for heavy metal ions removal from wastewater," *Polymers for Advanced Technologies,* vol. 32, no. 3, pp. 1000-1010, 2021.

[148] J. Zhu *et al.*, "PVDF mixed matrix ultrafiltration membrane incorporated with deformed rebar-like Fe3O4–palygorskite nanocomposites to enhance strength and antifouling properties," *Journal of membrane science,* vol. 612, p. 118467, 2020.

[149] A. Noormohamadi, M. Homayoonfal, M. R. Mehrnia, and F. Davar, "Synergistic effect of concurrent presence of zirconium oxide and iron oxide in the form of core-shell nanoparticles on the performance of Fe3O4@ZrO2 /PAN nanocomposite membrane," *Ceramics International,* vol. 43, no. 18, pp. 17174-17185, 2017/12/15/ 2017, doi: <https://doi.org/10.1016/j.ceramint.2017.09.142>.

[150] P. Moradihamedani, K. Kalantari, A. H. Abdullah, and N. A. Morad, "High efficient removal of lead (II) and nickel (II) from aqueous solution by novel polysulfone/Fe3O4–talc nanocomposite mixed matrix membrane," *Desalination and Water Treatment,* vol. 57, no. 59, pp. 28900-28909, 2016.

[151] S. Ansari, A. Moghadassi, and S. M. Hosseini, "A new approach to tailoring the separation characteristics of polyethersulfone nanofiltration membranes by 8-hydroxyquinoline functionalized Fe3O4 nanoparticles," *Korean Journal of Chemical Engineering,* vol. 37, no. 11, pp. 2011-2019, 2020.

[152] H. Liu, C. Wang, Y. Qin, Y. Huang, and C. Xiao, "Oriented structure design and evaluation of Fe3O4/o-MWCNTs/PVC composite membrane assisted by magnetic field," *Journal of the Taiwan Institute of Chemical Engineers,* vol. 120, pp. 278-290, 2021.

[153] Z.-H. Huang *et al.*, "Fe3O4/PVDF catalytic membrane treatment organic wastewater with simultaneously improved permeability, catalytic property and anti-fouling," *Environmental Research,* vol. 187, p. 109617, 2020.

[154] J. Sun, S. Li, Z. Ran, and Y. Xiang, "Preparation of Fe3O4@ TiO2 blended PVDF membrane by magnetic coagulation bath and its permeability and pollution resistance," *Journal of Materials Research and Technology,* vol. 9, no. 3, pp. 4951-4967, 2020.

[155] V. Vatanpour, S. Shahsavarifar, S. Khorshidi, and M. Masteri‐Farahani, "A novel antifouling ultrafiltration membranes prepared from percarboxylic acid functionalized SiO2 bound Fe3O4 nanoparticle (SCMNP‐COOOH)/polyethersulfone nanocomposite for BSA separation and dye removal," *Journal of Chemical Technology & Biotechnology,* vol. 94, no. 4, pp. 1341-1353, 2019.

[156] N. Ghaemi *et al.*, "Polyethersulfone membrane enhanced with iron oxide nanoparticles for copper removal from water: Application of new functionalized Fe3O4 nanoparticles," *Chemical Engineering Journal,* vol. 263, pp. 101-112, 2015.

[157] L. Dong, M. Li, S. Zhang, X. Si, Y. Bai, and C. Zhang, "NH2-Fe3O4-regulated graphene oxide membranes with well-defined laminar nanochannels for desalination of dye solutions," *Desalination,* vol. 476, p. 114227, 2020.

[158] N. S. M. Nawi, W. J. Lau, N. Yusof, N. Said, and A. F. Ismail, "Enhancing water flux and antifouling properties of PES hollow fiber membranes via incorporation of surface‐functionalized Fe3O4 nanoparticles," *Journal of Chemical Technology & Biotechnology,* vol. 97, no. 4, pp. 1006-1020, 2022.

[159] A. Abdel-Karim, S. H. Ismail, A. M. Bayoumy, M. Ibrahim, and G. G. Mohamed, "Antifouling PES/Cu@ Fe3O4 mixed matrix membranes: Quantitative structure–activity relationship (QSAR) modeling and wastewater treatment potentiality," *Chemical Engineering Journal,* vol. 407, p. 126501, 2021.

[160] J. Rowley and N. H. Abu-Zahra, "Synthesis and characterization of polyethersulfone membranes impregnated with (3-aminopropyltriethoxysilane) APTES-Fe3O4 nanoparticles for As (V) removal from water," *Journal of Environmental Chemical Engineering,* vol. 7, no. 1, p. 102875, 2019.

[161] A. Noormohamadi, M. Homayoonfal, M. R. Mehrnia, and F. J. E. t. Davar, "Employing magnetism of Fe3O4 and hydrophilicity of ZrO2 to mitigate biofouling in magnetic MBR by Fe3O4-coated ZrO2/PAN nanocomposite membrane," vol. 41, no. 20, pp. 2683-2704, 2020.

[162] V. Vatanpour *et al.*, "Fe3O4@ Gum Arabic modified polyvinyl chloride membranes to improve antifouling performance and separation efficiency of organic pollutants," *Chemosphere,* vol. 328, p. 138586, 2023.

[163] B. Li *et al.*, "Changing conventional blending photocatalytic membranes (BPMs): Focus on improving photocatalytic performance of Fe3O4/g-C3N4/PVDF membranes through magnetically induced freezing casting method," *Chemical Engineering Journal,* vol. 365, pp. 405-414, 2019.

[164] Z. Rahimi, A. A. Zinatizadeh, and S. Zinadini, "Milk processing wastewater treatment in a bioreactor followed by an antifouling O-carboxymethyl chitosan modified Fe3O4/PVDF ultrafiltration membrane," *Journal of Industrial and Engineering Chemistry,* vol. 38, pp. 103-112, 2016/06/25/ 2016, doi: <https://doi.org/10.1016/j.jiec.2016.04.011>.

[165] E. Gandomkar and A. Fazlali, "Small but Mighty: Incorporating Fe3O4 Nanoparticles into PES Membranes for Enhanced Water Treatment Efficiency," *Iran. J. Chem. Chem. Eng. Research Article Vol,* vol. 42, no. 10, 2023.

[166] S. Koushkbaghi, A. Zakialamdari, M. Pishnamazi, H. F. Ramandi, M. Aliabadi, and M. Irani, "Aminated-Fe3O4 nanoparticles filled chitosan/PVA/PES dual layers nanofibrous membrane for the removal of Cr (VI) and Pb (II) ions from aqueous solutions in adsorption and membrane processes," *Chemical Engineering Journal,* vol. 337, pp. 169-182, 2018.

[167] Z. Rahimi, A. Zinatizadeh, and S. J. E. t. Zinadini, "Membrane bioreactors troubleshooting through the preparation of a high antifouling PVDF ultrafiltration mixed-matrix membrane blended with O-carboxymethyl chitosan-Fe3O4 nanoparticles," 2018.

[168] M. Nasir *et al.*, "The GO-Fe3O4/Psf Membrane Prepared by Phase Inversion for Filtration: Dyes and NaCl in Water," *Journal of Water and Environmental Nanotechnology,* vol. 8, no. 3, pp. 241-253, 2023.

[169] S. Ansari, E. Bagheripour, A. Moghadassi, and S. M. Hosseini, "Fabrication of mixed matrix poly (phenylene ether-ether sulfone)-based nanofiltration membrane modified by Fe3O4 nanoparticles for water desalination," *Journal of Polymer Engineering,* vol. 37, no. 1, pp. 61-67, 2017.

[170] S. Kamari and A. Shahbazi, "Biocompatible Fe3O4@ SiO2-NH2 nanocomposite as a green nanofiller embedded in PES–nanofiltration membrane matrix for salts, heavy metal ion and dye removal: Long–term operation and reusability tests," *Chemosphere,* vol. 243, p. 125282, 2020.

[171] K. E. Mokubung, L. N. Ndlovu, W. J. Lau, E. N. Nxumalo, and N. N. Gumbi, "Enhanced adsorptive removal of As (V) ions in aqueous solution using polyethersulfone ultrafiltration mixed matrix membranes impregnated with 3‐aminopropyltriethoxysilane modified magnetite Fe3O4 nanoparticles," *Journal of Applied Polymer Science,* vol. 140, no. 24, p. e53944, 2023.

[172] E. Bagheripour, A. Moghadassi, and S. M. Hosseini, "Incorporated poly acrylic acid-co-fe3o4 nanoparticles mixed matrix polyethersulfone based nanofiltration membrane in desalination process," *International Journal Of Engineering,* vol. 30, no. 6, pp. 821-829, 2017.

[173] P. Daraei, S. S. Madaeni, N. Ghaemi, M. A. Khadivi, B. Astinchap, and R. Moradian, "Fouling resistant mixed matrix polyethersulfone membranes blended with magnetic nanoparticles: Study of magnetic field induced casting," *Separation and Purification Technology,* vol. 109, pp. 111-121, 2013.

[174] M. Ouda *et al.*, "Surface tuned polyethersulfone membrane using an iron oxide functionalized halloysite nanocomposite for enhanced humic acid removal," *Environmental Research,* vol. 204, p. 112113, 2022.

[175] P. Chai, E. Mahmoudi, Y. Teow, and A. Mohammad, "Preparation of novel polysulfone-Fe3O4/GO mixed-matrix membrane for humic acid rejection," *Journal of Water Process Engineering,* vol. 15, pp. 83-88, 2017.

[176] T. A. Agbaje, S. Al-Gharabli, M. O. Mavukkandy, J. Kujawa, and H. A. Arafat, "PVDF/magnetite blend membranes for enhanced flux and salt rejection in membrane distillation," *Desalination,* vol. 436, pp. 69-80, 2018.

[177] R. R. Darabi, M. Jahanshahi, and M. Peyravi, "A support assisted by photocatalytic Fe3O4/ZnO nanocomposite for thin-film forward osmosis membrane," *Chemical Engineering Research and Design,* vol. 133, pp. 11-25, 2018.

[178] H. M. Mezher, H. Adeli, and Q. F. Alsalhy, "Novel ZnO-Modified Polyethersulfone Nanocomposite Membranes for Nanofiltration of Concentrated Textile Wastewater," *Water, Air, & Soil Pollution,* vol. 235, no. 2, p. 138, 2024.

[179] N. Nasrollahi, S. Aber, V. Vatanpour, and N. M. Mahmoodi, "The effect of amine functionalization of CuO and ZnO nanoparticles used as additives on the morphology and the permeation properties of polyethersulfone ultrafiltration nanocomposite membranes," *Composites Part B: Engineering,* vol. 154, pp. 388-409, 2018.

[180] S. Liang, K. Xiao, Y. Mo, and X. Huang, "A novel ZnO nanoparticle blended polyvinylidene fluoride membrane for anti-irreversible fouling," *Journal of membrane science,* vol. 394, pp. 184-192, 2012.

[181] T. D. Kusworo and D. P. Utomo, "Performance evaluation of double stage process using nano hybrid PES/SiO2-PES membrane and PES/ZnO-PES membranes for oily waste water treatment to clean water," *Journal of environmental chemical engineering,* vol. 5, no. 6, pp. 6077-6086, 2017.

[182] O. T. Mahlangu, G. Mamba, and B. B. Mamba, "A facile synthesis approach for GO-ZnO/PES ultrafiltration mixed matrix photocatalytic membranes for dye removal in water: Leveraging the synergy between photocatalysis and membrane filtration," *Journal of Environmental Chemical Engineering,* vol. 11, no. 3, p. 110065, 2023.

[183] N. Nasrollahi, V. Vatanpour, S. Aber, and N. M. Mahmoodi, "Preparation and characterization of a novel polyethersulfone (PES) ultrafiltration membrane modified with a CuO/ZnO nanocomposite to improve permeability and antifouling properties," *Separation and Purification Technology,* vol. 192, pp. 369-382, 2018/02/09/ 2018, doi: <https://doi.org/10.1016/j.seppur.2017.10.034>.

[184] T. A. Otitoju, M. Ahmadipour, S. Li, N. F. Shoparwe, L. X. Jie, and A. L. Owolabi, "Influence of nanoparticle type on the performance of nanocomposite membranes for wastewater treatment," *Journal of Water Process Engineering,* vol. 36, p. 101356, 2020.

[185] M. Zou *et al.*, "Exploring the potential of flexible CdS/ZnO/Polyurethane nanocomposite membrane for wastewater remediation," *Journal of Environmental Chemical Engineering,* vol. 11, no. 3, p. 110135, 2023.

[186] S. Zinadini, S. Rostami, V. Vatanpour, and E. J. J. o. M. S. Jalilian, "Preparation of antibiofouling polyethersulfone mixed matrix NF membrane using photocatalytic activity of ZnO/MWCNTs nanocomposite," vol. 529, pp. 133-141, 2017.

[187] X. Chen, C. Huang, R. Feng, P. Zhang, Y. Wu, and W. Huang, "Multifunctional PVDF Membrane Coated with ZnO-Ag Nanocomposites for Wastewater Treatment and Fouling Mitigation: Factorial and Mechanism Analyses," *Journal of Environmental Informatics,* vol. 41, no. 1, 2023.

[188] H. Etemadi, H. Khezraqa, and M. Hermani, "Incorporation of amino‐functionalized ZnO nanoparticles into polycarbonate/polyvinyl alcohol thin‐film membrane for enhanced water treatment," *Polymer Engineering & Science,* vol. 62, no. 9, pp. 2891-2899, 2022.

[189] R. B. Soria, J. Zhu, I. Gonza, B. Van der Bruggen, and P. Luis, "Effect of (TiO2: ZnO) ratio on the anti-fouling properties of bio-inspired nanofiltration membranes," *Separation and Purification Technology,* vol. 251, p. 117280, 2020.

[190] T. D. Kusworo, A. C. Kumoro, N. Aryanti, T. A. Kurniawan, F. Dalanta, and N. H. Alias, "Photocatalytic polysulfone membrane incorporated by ZnO-MnO2@ SiO2 composite under UV light irradiation for the reliable treatment of natural rubber-laden wastewater," *Chemical Engineering Journal,* vol. 451, p. 138593, 2023.

[191] M. El-Noss, H. Isawi, H. A. Shawky, M. Gomaa, and M. Abdel-Mottaleb, "Improvement of cellulose acetate forward osmosis membrane performance using zinc oxide nanoparticles," *Desalination Water Treat,* vol. 193, pp. 19-33, 2020.

[192] R. R. Darabi, M. Peyravi, and M. Jahanshahi, "Modified forward osmosis membranes by two amino-functionalized ZnO nanoparticles: a comparative study," *Chemical Engineering Research and Design,* vol. 145, pp. 85-98, 2019.

[193] F. Wahid *et al.*, "Fabrication of bacterial cellulose with TiO2-ZnO nanocomposites as a multifunctional membrane for water remediation," *Journal of Colloid and Interface Science,* vol. 620, pp. 1-13, 2022.

[194] F. Sokhandan, M. Homayoonfal, and F. Davar, "Application of zinc oxide and sodium alginate for biofouling mitigation in a membrane bioreactor treating urban wastewater," *Biofouling,* vol. 36, no. 6, pp. 660-678, 2020.

[195] A. Mollahosseini and A. Rahimpour, "A new concept in polymeric thin-film composite nanofiltration membranes with antibacterial properties," *Biofouling,* vol. 29, no. 5, pp. 537-548, 2013.

[196] M. Ahsani, H. Hazrati, M. Javadi, M. Ulbricht, and R. Yegani, "Preparation of antibiofouling nanocomposite PVDF/Ag-SiO2 membrane and long-term performance evaluation in the MBR system fed by real pharmaceutical wastewater," *Separation and Purification Technology,* vol. 249, p. 116938, 2020.

[197] E.-S. Kim, G. Hwang, M. G. El-Din, and Y. Liu, "Development of nanosilver and multi-walled carbon nanotubes thin-film nanocomposite membrane for enhanced water treatment," *Journal of membrane science,* vol. 394, pp. 37-48, 2012.

[198] H. Abadikhah, E. Naderi Kalali, S. Khodi, X. Xu, and S. Agathopoulos, "Multifunctional thin-film nanofiltration membrane incorporated with reduced graphene oxide@ TiO2@ Ag nanocomposites for high desalination performance, dye retention, and antibacterial properties," *ACS applied materials & interfaces,* vol. 11, no. 26, pp. 23535-23545, 2019.

[199] A. Behboudi, T. Mohammadi, and M. Ulbricht, "High performance antibiofouling hollow fiber polyethersulfone nanocomposite membranes incorporated with novel surface-modified silver nanoparticles suitable for membrane bioreactor application," *Journal of Industrial and Engineering Chemistry,* vol. 119, pp. 298-314, 2023.

[200] Y. Lee, T. Kim, B. Kim, S. Choi, and K. Kim, "Synthesis of TiO2/MoSx/Ag nanocomposites via photodeposition for enhanced photocatalysis and membrane fouling mitigation," *Journal of Environmental Chemical Engineering,* vol. 11, no. 2, p. 109266, 2023.

[201] H. Chen *et al.*, "Enhancing rejection performance of tetracycline resistance genes by a TiO2/AgNPs-modified nanofiber forward osmosis membrane," *Chemical Engineering Journal,* vol. 382, p. 123052, 2020.

[202] V. Vatanpour *et al.*, "Investigation of boron nitride/silver/graphene oxide nanocomposite on separation and antibacterial improvement of polyethersulfone membranes in wastewater treatment," *Journal of Environmental Chemical Engineering,* vol. 10, no. 1, p. 107035, 2022.

[203] J. R. Mishra, S. K. Samal, S. Mohanty, and S. K. Nayak, "Polyvinylidene fluoride (PVDF)/Ag@ TiO2 nanocomposite membrane with enhanced fouling resistance and antibacterial performance," *Materials Chemistry and Physics,* vol. 268, p. 124723, 2021.

[204] J. Huang, H. Wang, and K. Zhang, "Modification of PES membrane with Ag–SiO2: Reduction of biofouling and improvement of filtration performance," *Desalination,* vol. 336, pp. 8-17, 2014.

[205] H. Abadikhah *et al.*, "High flux thin film nanocomposite membrane incorporated with functionalized TiO2@ reduced graphene oxide nanohybrids for organic solvent nanofiltration," *Chemical Engineering Science,* vol. 204, pp. 99-109, 2019.

[206] K. Kotlhao, V. E. Pakade, F. M. Mtunzi, R. M. Moutloali, and M. J. Klink, "Preparation and Characterization of Ag–TiO 2 Modified Polyethersulfone (PES) Membranes for Potential Applications in Water Treatment," in *Chemistry for a Clean and Healthy Planet*, 2019: Springer, pp. 331-349.

[207] J. Li, S. Yuan, J. Zhu, and B. Van der Bruggen, "High-flux, antibacterial composite membranes via polydopamine-assisted PEI-TiO2/Ag modification for dye removal," *Chemical Engineering Journal,* vol. 373, pp. 275-284, 2019.

[208] K. Monsef, M. Homayoonfal, and F. Davar, "Engineering arrangement of nanoparticles within nanocomposite membranes matrix: a suggested way to enhance water flux," *Polymer-Plastics Technology and Materials,* vol. 59, no. 7, pp. 733-752, 2020.

[209] N. Maximous, G. Nakhla, W. Wan, and K. Wong, "Performance of a novel ZrO2/PES membrane for wastewater filtration," *Journal of Membrane Science,* vol. 352, no. 1-2, pp. 222-230, 2010.

[210] S. Zarghami, T. Mohammadi, M. Sadrzadeh, and B. Van der Bruggen, "Bio-inspired anchoring of amino-functionalized multi-wall carbon nanotubes (N-MWCNTs) onto PES membrane using polydopamine for oily wastewater treatment," *Science of the total environment,* vol. 711, p. 134951, 2020.

[211] L.-x. Dong, X.-c. Huang, Z. Wang, Z. Yang, X.-m. Wang, and C. Y. Tang, "A thin-film nanocomposite nanofiltration membrane prepared on a support with in situ embedded zeolite nanoparticles," *Separation and Purification Technology,* vol. 166, pp. 230-239, 2016.

[212] K. Monsef, M. Homayoonfal, and F. Davar, "Coating carboxylic and sulfate functional groups on ZrO2 nanoparticles: Antifouling enhancement of nanocomposite membranes during water treatment," *Reactive and Functional Polymers,* vol. 131, pp. 299-314, 2018.

[213] M. Fathizadeh, A. Aroujalian, and A. Raisi, "Effect of added NaX nano-zeolite into polyamide as a top thin layer of membrane on water flux and salt rejection in a reverse osmosis process," *Journal of membrane science,* vol. 375, no. 1-2, pp. 88-95, 2011.

[214] H.-R. Chae, C.-H. Lee, P.-K. Park, I.-C. Kim, and J.-H. Kim, "Synergetic effect of graphene oxide nanosheets embedded in the active and support layers on the performance of thin-film composite membranes," *Journal of Membrane Science,* vol. 525, pp. 99-106, 2017.

[215] V. Santosh, P. V. Babu, J. Gopinath, N. N. M. Rao, A. V. S. Sainath, and A. Reddy, "Development of hydroxyl and carboxylic acid functionalized CNTs–polysulphone nanocomposite fouling-resistant ultrafiltration membranes for oil–water separation," *Bulletin of Materials Science,* vol. 43, pp. 1-12, 2020.

[216] X. Guo, D. Liu, T. Han, H. Huang, Q. Yang, and C. Zhong, "Preparation of thin film nanocomposite membranes with surface modified MOF for high flux organic solvent nanofiltration," *AIChE Journal,* vol. 63, no. 4, pp. 1303-1312, 2017.

[217] J. Bose, J. Dasgupta, U. Adhikari, and J. Sikder, "Tuning permeation characteristics of cellulose acetate membrane embedded with raw and amine-functionalized silicon carbide nanoparticle for oil-water separation," *Journal of Water Process Engineering,* vol. 41, p. 102019, 2021.

[218] F. M. Bojnourd and M. Pakizeh, "Preparation and characterization of a nanoclay/PVA/PSf nanocomposite membrane for removal of pharmaceuticals from water," *Applied Clay Science,* vol. 162, pp. 326-338, 2018.

[219] S. S. H. Zahed, S. Khanlari, and T. Mohammadi, "Hydrous metal oxide incorporated polyacrylonitrile-based nanocomposite membranes for Cu (II) ions removal," *Separation and Purification Technology,* vol. 213, pp. 151-161, 2019.

[220] M. M. Pendergast, A. K. Ghosh, and E. Hoek, "Separation performance and interfacial properties of nanocomposite reverse osmosis membranes," *Desalination,* vol. 308, pp. 180-185, 2013.

[221] S. Basu and M. Balakrishnan, "Polyamide thin film composite membranes containing ZIF-8 for the separation of pharmaceutical compounds from aqueous streams," *Separation and Purification Technology,* vol. 179, pp. 118-125, 2017.

[222] M. A. Halakarni *et al.*, "Design of selective and self-cleaning iron aminoclay thin film nanocomposite membranes," *Chemical Engineering Journal,* vol. 456, p. 140941, 2023.

[223] M. Homayoonfal, M. R. Mehrnia, S. Rahmani, and Y. M. Mojtahedi, "Fabrication of alumina/polysulfone nanocomposite membranes with biofouling mitigation approach in membrane bioreactors," *Journal of Industrial and Engineering Chemistry,* vol. 22, pp. 357-367, 2015.

[224] S. Samsami, M.-H. Sarrafzadeh, and A. Ahmadi, "Surface modification of thin-film nanocomposite forward osmosis membrane with super-hydrophilic MIL-53 (Al) for doxycycline removal as an emerging contaminant and membrane antifouling property enhancement," *Chemical Engineering Journal,* vol. 431, p. 133469, 2022.

[225] T. Subrahmanya *et al.*, "Effect of functionalized nanodiamonds and surfactants mediation on the nanofiltration performance of polyamide thin-film nanocomposite membranes," *Desalination,* vol. 555, p. 116540, 2023.

[226] T. Hashemi, M. R. Mehrnia, and S. Ghezelgheshlaghi, "Influence of alumina nanoparticles on the performance of polyacrylonitrile membranes in MBR," *Journal of Environmental Health Science and Engineering,* vol. 20, no. 1, pp. 375-384, 2022.

[227] L. Zhang, M. Zhang, J. Lu, A. Tang, and L. Zhu, "Highly permeable thin-film nanocomposite membranes embedded with PDA/PEG nanocapsules as water transport channels," *Journal of Membrane Science,* vol. 586, pp. 115-121, 2019.

[228] J. Sun *et al.*, "Amino-embedded carbon quantum dots incorporated thin-film nanocomposite membrane for desalination by pervaporation," *Desalination,* vol. 533, p. 115742, 2022.

[229] R. Krishnamoorthy and V. Sagadevan, "Polyethylene glycol and iron oxide nanoparticles blended polyethersulfone ultrafiltration membrane for enhanced performance in dye removal studies," *e-Polymers,* vol. 15, no. 3, pp. 151-159, 2015.

[230] G. Moradi, S. Zinadini, and L. Rajabi, "Development of high flux nanofiltration membrane using para-amino benzoate ferroxane nanoparticle for enhanced antifouling behavior and dye removal," *Process Safety and Environmental Protection,* vol. 144, pp. 65-78, 2020.

[231] X. Li, C. Zhao, M. Yang, B. Yang, D. Hou, and T. Wang, "Reduced graphene oxide-NH2 modified low pressure nanofiltration composite hollow fiber membranes with improved water flux and antifouling capabilities," *Applied Surface Science,* vol. 419, pp. 418-428, 2017.

[232] T. Tavangar, M. Karimi, M. Rezakazemi, K. R. Reddy, and T. M. Aminabhavi, "Textile waste, dyes/inorganic salts separation of cerium oxide-loaded loose nanofiltration polyethersulfone membranes," *Chemical Engineering Journal,* vol. 385, p. 123787, 2020.

[233] M. L. Lind, D. Eumine Suk, T.-V. Nguyen, and E. M. Hoek, "Tailoring the structure of thin film nanocomposite membranes to achieve seawater RO membrane performance," *Environmental science & technology,* vol. 44, no. 21, pp. 8230-8235, 2010.

[234] L. Xu, T. Yang, M. Li, J. Chang, and J. Xu, "Thin-film nanocomposite membrane doped with carboxylated covalent organic frameworks for efficient forward osmosis desalination," *Journal of membrane science,* vol. 610, p. 118111, 2020.

[235] H. Koulivand, A. Shahbazi, V. Vatanpour, and M. Rahmandoust, "Development of carbon dot-modified polyethersulfone membranes for enhancement of nanofiltration, permeation and antifouling performance," *Separation and Purification Technology,* vol. 230, p. 115895, 2020.

[236] N. Ahmad, A. Samavati, N. A. H. M. Nordin, J. Jaafar, A. F. Ismail, and N. A. N. N. Malek, "Enhanced performance and antibacterial properties of amine-functionalized ZIF-8-decorated GO for ultrafiltration membrane," *Separation and Purification Technology,* vol. 239, p. 116554, 2020.

[237] X. Wang, Y. Liu, K. Fan, P. Cheng, H. Xia, and S. Xia, "Utilization of carboxyl group-grafted molybdenum disulfide for enhancing the performance of thin-film nanocomposite nanofiltration membranes," *Desalination,* vol. 548, p. 116283, 2023.

[238] Z. Zabihi and M. Homayoonfal, "Strategies to modify the structure of thin‐film composite membranes for advanced separation of metronidazole antibiotic from wastewater," *Polymers for Advanced Technologies,* vol. 32, no. 12, pp. 4765-4786, 2021.

[239] J. Bose, L. Marchio, U. Adhikari, D. Datta, and J. Sikder, "Synthesis and characterization of polyvinylidene fluoride/functionalized silicon carbide nanocomposite membrane for water treatment," *Journal of Polymer Research,* vol. 30, no. 6, p. 246, 2023.

[240] H. Etemadi, R. Yegani, and V. Babaeipour, "Performance evaluation and antifouling analyses of cellulose acetate/nanodiamond nanocomposite membranes in water treatment," *Journal of applied polymer science,* vol. 134, no. 21, 2017.

[241] M. Khajouei, M. Jahanshahi, M. Peyravi, H. Hoseinpour, and A. S. Rad, "Anti-bacterial assay of doped membrane by zero valent Fe nanoparticle via in-situ and ex-situ aspect," *Chemical Engineering Research and Design,* vol. 117, pp. 287-300, 2017.

[242] A. Yousefi, H. Etemadi, M. Hermani, F. Aftabi, and G. Hosseinzadeh, "Preparation and performance evaluation of PVC/PDA-modified Al2O3 nanocomposite membranes in oily wastewater treatment," *Journal of Inorganic and Organometallic Polymers and Materials,* vol. 33, no. 4, pp. 1066-1079, 2023.

[243] S. R. Lakhotia, M. Mukhopadhyay, and P. Kumari, "Iron oxide (FeO) nanoparticles embedded thin-film nanocomposite nanofiltration (NF) membrane for water treatment," *Separation and Purification Technology,* vol. 211, pp. 98-107, 2019.

[244] A. Tizchang, Y. Jafarzadeh, R. Yegani, and S. Khakpour, "The effects of pristine and silanized nanodiamond on the performance of polysulfone membranes for wastewater treatment by MBR system," *Journal of Environmental Chemical Engineering,* vol. 7, no. 6, p. 103447, 2019.

[245] S. Gholami, J. L. Llacuna, V. Vatanpour, A. Dehqan, S. Paziresh, and J. L. Cortina, "Impact of a new functionalization of multiwalled carbon nanotubes on antifouling and permeability of PVDF nanocomposite membranes for dye wastewater treatment," *Chemosphere,* vol. 294, p. 133699, 2022.

[246] S. Mishra, A. K. Singh, and J. K. Singh, "Ferrous sulfide and carboxyl-functionalized ferroferric oxide incorporated PVDF-based nanocomposite membranes for simultaneous removal of highly toxic heavy-metal ions from industrial ground water," *Journal of membrane science,* vol. 593, p. 117422, 2020.

[247] H. Etemadi, R. Yegani, and M. Seyfollahi, "The effect of amino functionalized and polyethylene glycol grafted nanodiamond on anti-biofouling properties of cellulose acetate membrane in membrane bioreactor systems," *Separation and Purification Technology,* vol. 177, pp. 350-362, 2017/04/28/ 2017, doi: <https://doi.org/10.1016/j.seppur.2017.01.013>.

[248] R. Moeinzadeh, A. G. J. Ghadam, W. J. Lau, and D. Emadzadeh, "Synthesis of nanocomposite membrane incorporated with amino-functionalized nanocrystalline cellulose for refinery wastewater treatment," *Carbohydrate polymers,* vol. 225, p. 115212, 2019.

[249] M. S. S. A. Saraswathi, D. Rana, K. Divya, S. Gowrishankar, and A. Nagendran, "Versatility of hydrophilic and antifouling PVDF ultrafiltration membranes tailored with polyhexanide coated copper oxide nanoparticles," *Polymer Testing,* vol. 84, p. 106367, 2020.

[250] P. Kallem, I. Othman, M. Ouda, S. W. Hasan, I. AlNashef, and F. Banat, "Polyethersulfone hybrid ultrafiltration membranes fabricated with polydopamine modified ZnFe2O4 nanocomposites: Applications in humic acid removal and oil/water emulsion separation," *Process Safety and Environmental Protection,* vol. 148, pp. 813-824, 2021.

[251] H. Zeng *et al.*, "A novel strategy for enhancing the performance of membranes for dyes separation: Embedding PAA@ UiO-66-NH2 between graphene oxide sheets," *Chemical Engineering Journal,* vol. 403, p. 126281, 2021.

[252] Y. Wang *et al.*, "Improving permeability and anti-fouling performance in reverse osmosis application of polyamide thin film nanocomposite membrane modified with functionalized carbon nanospheres," *Separation and Purification Technology,* vol. 270, p. 118828, 2021.

[253] N. H. Z. Abidin, S. N. A. Shafie, H. Suhaimi, N. S. Sambudi, and N. A. H. S. M. Nordin, "Incorporation of carboxyl and amino functionalized carbon quantum dots in thin film membrane for nanofiltration," *Polymer Testing,* vol. 100, p. 107270, 2021.

[254] Y. Wang, J. Ma, J. Zhu, N. Ye, X. Zhang, and H. Huang, "Multi-walled carbon nanotubes with selected properties for dynamic filtration of pharmaceuticals and personal care products," *Water research,* vol. 92, pp. 104-112, 2016.

[255] L. Paseta, D. Antoran, J. Coronas, and C. Tellez, "110th anniversary: polyamide/metal–organic framework bilayered thin film composite membranes for the removal of pharmaceutical compounds from water," *Industrial & Engineering Chemistry Research,* vol. 58, no. 10, pp. 4222-4230, 2019.

[256] E. Abdollahi, A. Heidari, T. Mohammadi, A. A. Asadi, and M. A. Tofighy, "Application of Mg-Al LDH nanoparticles to enhance flux, hydrophilicity and antifouling properties of PVDF ultrafiltration membrane: Experimental and modeling studies," *Separation and Purification Technology,* vol. 257, p. 117931, 2021.
